# Intergenerational environmental effects: functional signals in offspring transcriptomes and metabolomes after parental jasmonic acid treatment in apomictic dandelion

**DOI:** 10.1111/nph.14835

**Published:** 2017-10-16

**Authors:** Koen J. F. Verhoeven, Eline H. Verbon, Thomas P. van Gurp, Carla Oplaat, Julie Ferreira de Carvalho, Alison M. Morse, Mark Stahl, Mirka Macel, Lauren M. McIntyre

**Affiliations:** ^1^ Terrestrial Ecology Netherlands Institute of Ecology (NIOO‐KNAW) Droevendaalsesteeg 10 Wageningen the Netherlands; ^2^ Plant–Microbe Interactions Utrecht University Padualaan 6 Utrecht the Netherlands; ^3^ Molecular Genetics and Microbiology, and the Genetics Institute University of Florida 2033 Mowry Road Gainesville FL 32610 USA; ^4^ Center for Plant Molecular Biology Tübingen University Auf der Morgenstelle 32 Tübingen D‐72076 Germany; ^5^ Molecular Interaction Ecology Department of Plant Science Radboud University Nijmegen PO Box 9010 Nijmegen 6500 NL the Netherlands

**Keywords:** induced defenses, jasmonic acid (JA), LC‐MS, metabolomics, RNA‐seq, *Taraxacum officinale* (common dandelion), transcriptomics, transgenerational effects

## Abstract

- Parental environments can influence offspring traits. However, the magnitude of the impact of parental environments on offspring molecular phenotypes is poorly understood. Here, we test the direct effects and intergenerational effects of jasmonic acid (JA) treatment, which is involved in herbivory‐induced defense signaling, on transcriptomes and metabolomes in apomictic common dandelion (*Taraxacum officinale*).
- In a full factorial crossed design with parental and offspring JA and control treatments, we performed leaf RNA‐seq gene expression analysis, LC‐MS metabolomics and total phenolics assays in offspring plants.
- Expression analysis, leveraged by a *de novo* assembled transcriptome, revealed an induced response to JA exposure that is consistent with known JA effects. The intergenerational effect of treatment was considerable: 307 of 858 detected JA‐responsive transcripts were affected by parental JA treatment. In terms of the numbers of metabolites affected, the magnitude of the chemical response to parental JA exposure was *c*. 10% of the direct JA treatment response. Transcriptome and metabolome analyses both identified the phosphatidylinositol signaling pathway as a target of intergenerational JA effects.
- Our results highlight that parental environments can have substantial effects in offspring generations. Transcriptome and metabolome assays provide a basis for zooming in on the potential mechanisms of inherited JA effects.

## Introduction

The phenotype of a plant can be affected by the environmental experiences of its direct ancestors through effects on the parents that are transmitted to the offspring. Although parental (or intergenerational) effects can be non‐adaptive, they sometimes ‘prepare’ offspring for enhanced performance when the offspring experiences similar environmental stresses to the parents (Galloway & Etterson, 2007; Holeski *et al*., 2012). In such cases, intergenerational effects may be evolved adaptive responses to environmental stresses, extending adaptive phenotypic plasticity across generations (Herman *et al*., 2014).

One area in which parental effects are thought to be particularly relevant is in plant–insect and plant–pathogen interactions. Within a single generation, priming of systemic tissue for enhanced defense has been well documented and can be induced by pathogen attack or other cues (Fu & Dong, 2013; Pieterse *et al*., 2014). Priming of the defense response does not constitutively activate defense responses, but results in a more rapid activation of the defense response on subsequent pathogen attack. Such effects that are induced by pathogens or herbivores can persist to offspring (Holeski, 2007) and, in some cases, effects are sustained for multiple generations (Luna *et al*., 2012; Rasmann *et al*., 2012). The underlying mechanisms are not completely understood, but mounting evidence for durable epigenetic changes in response to environmental cues (Feil & Fraga, 2012) indicates at least one possible mechanism.

Although parental environmental effects on offspring phenotypes have been shown repeatedly, there is little knowledge of the extent to which gene expression is affected by parental environment, and the limited available data to date show mixed results. Examples of transgenerational effects in plants are associated with histone modifications at defense gene promoters (Luna *et al*., 2012) and with small interfering RNA (siRNA) (Rasmann *et al*., 2012), implicating epigenetic gene regulation in offspring after parental exposure. Exposure of *Arabidopsis thaliana* to the bacterial elicitor flagellin has been reported to increase homologous recombination frequencies in this plant and several subsequent generations; however, whole‐transcriptome microarray analysis revealed no effects on offspring gene expression (Molinier *et al*., 2006). In sharp contrast, artificial leaf herbivory in *Mimulus guttatus* triggers gene expression changes at nearly 1000 genes in untreated offspring (Colicchio *et al*., 2015).

In addition to up‐ or downregulation of specific genes in offspring, *variability* in offspring gene expression may also be affected. Increased variability can arise as a result of variable penetrance amongst offspring individuals or of epigenetic mutations that are triggered stochastically in germline tissue in response to stress. In either scenario, the result is hypothesized to be a bet‐hedging strategy to increase levels of phenotypic variation amongst offspring, which may be adaptive when environments are variable (Levy *et al*., 2012; Herman *et al*., 2014).

Better insight into the consequences of parental environmental effects on offspring gene expression is important to understand the ecological role and evolution of phenotypic plasticity. From a practical perspective, it is also relevant to determine whether parental environments should be taken into account in the set‐up of transcriptomic studies in general, which do not always control for pre‐experiment variation. Here, we use RNA‐seq expression profiling and LC‐MS metabolomics in the apomictic common dandelion (*Taraxacum officinale*) to evaluate how leaf gene expression and metabolites are affected in offspring as a result of jasmonic acid (JA) treatment in the parental generation. JA is a plant signaling hormone involved in various processes, including the regulation of growth and responses to biotic and abiotic stresses (Wasternack & Hause, 2013), and plays a major role in the induction of plant chemical defenses in response to herbivory. The application of JA solutions to plants generally elicits an induction of chemical defenses that is systemic (e.g. Schenk *et al*., 2000; van Dam *et al*., 2004; De Vos *et al*., 2005; Tytgat *et al*., 2013).

*Taraxacum officinale* is a convenient natural model system for such studies because of its apomictic reproduction through clonal seeds (van Dijk, 2003), which permits an evaluation of transgenerational effects in the absence of genetic differences between experimental plants. In *T. officinale*, effects of parental JA treatment on offspring epigenetic profiles (Verhoeven *et al*., 2010) and on offspring resistance to caterpillar feeding (Verhoeven & van Gurp, 2012) have been reported previously, showing a potential role for epigenetically mediated parental effects on herbivore resistance in this species. In this study, we specifically aimed to: evaluate the intergenerational gene expression response, in terms of effects on gene expression means and variances, after parental JA treatment; and determine whether a parental effect of JA is associated with modified offspring leaf (secondary) chemistry including defense compounds.

## Materials and Methods

### Plant material and experimental design

The common dandelion, *T. officinale* (F.H. Wigg.), is a widespread perennial plant species which has diploid sexual and polyploid (mostly triploid) obligate apomictic variants (van Dijk, 2003). Apomixis in dandelion is through meiotic diplospory followed by parthenogenetic embryo development from unreduced egg cells and autonomous endosperm development (Koltunow, 1993), which is thought to result in seeds that are clonal copies of the heterozygous mother plant. For this study, we used a single triploid apomictic genotype (A68), an accession collected near Heteren (the Netherlands) which had been propagated for multiple generations under common glasshouse conditions before the experiment. This genotype has been studied previously in the context of parental effects (Verhoeven & van Gurp, 2012). Some genomic resources are available for *T. officinale*, including an expressed sequence tag (EST) database (Compositae Genome Project, compgenomics.ucdavis.edu) and a *de novo* assembled transcriptome based on RNA‐seq data of a different apomictic genotype than used for the current study (Ferreira de Carvalho *et al*., 2016), but currently no annotated reference genome has been published. For this study, we generated a new *de novo* assembled reference transcriptome specific for the A68 apomictic genotype (see below).

#### Parental generation

Eight ‘control’ and eight ‘JA’ parental treatment lineages were derived from a single A68 founder individual by subjecting plants for two subsequent generations to either JA or control treatments under common climate chamber conditions (14 h : 10 h, light : dark at 20°C : 15°C, fully randomized pots) using single‐seed descent between the generations. Exposing two subsequent generations to the same environmental stress can enhance parental effects compared with single‐generation parental exposure (Wibowo *et al*., 2016). Based on previous experience in dandelion (Verhoeven & van Gurp, 2012), JA treatment was applied as a 10 mM JA solution (Sigma J‐2500, dissolved in ethanol and diluted to the desired concentration with a 0.1% Triton X‐100 surfactant solution) to the upper surface of three to four fully expanded leaves. In generation 1, 0.75 ml JA solution per plant was applied to 8‐wk‐old plants; in generation 2, a total amount of 0.75 ml was applied to each plant distributed over two application treatments when plants were 5 and 7 wk old. In both generations, JA was applied during vegetative growth, *c*. 1 month before first flowering.

#### Experimental generation

**Figure 1:**
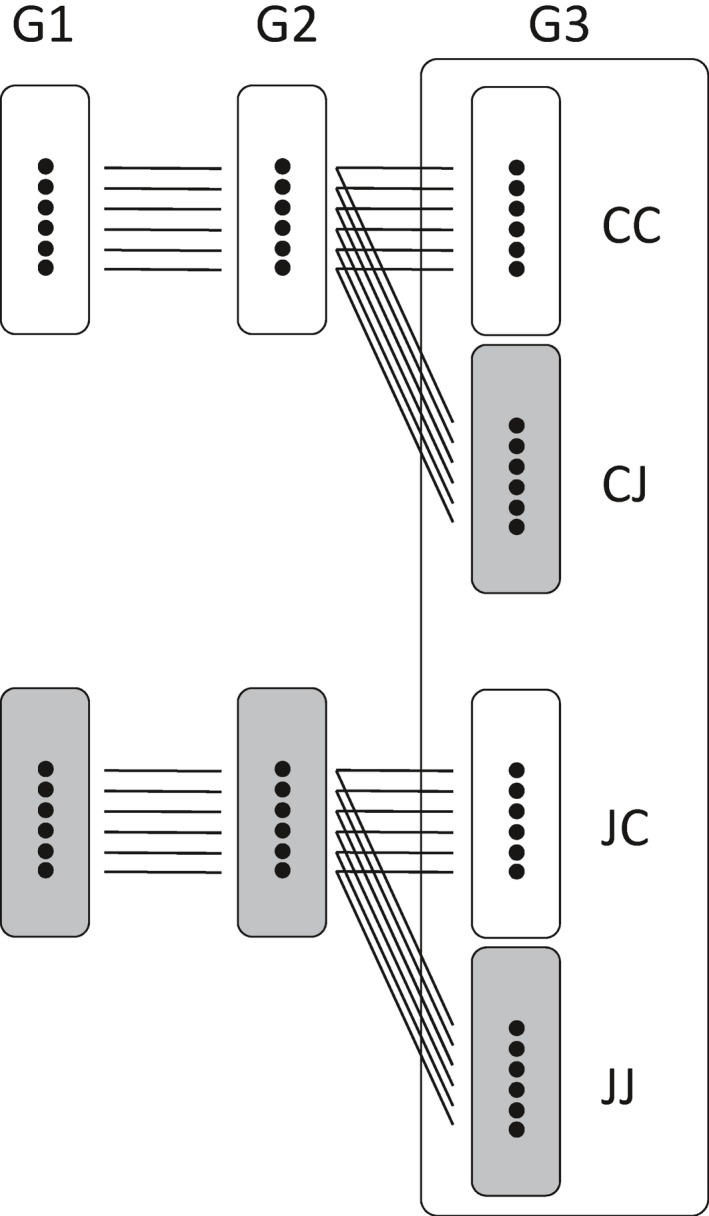
Experimental design. Common dandelion plants were exposed to jasmonic acid (JA) treatment (gray boxes) or control (white boxes). G1 and G2 indicate the ‘parental’ treatment. Transcriptome and metabolome analyses were performed in G3 plants, assessing the effects of both experimental (direct) JA treatment and parental JA treatment. The four experimental G3 groups are coded as CC, CJ, JC and JJ, where the first letter denotes the parental treatment (J = JA, C = control) and the second letter denotes the treatment of the G3 experimental plants. For chemical analyses, eight replicates per group were used but, for RNA‐seq analysis, only six replicates per group were used.

For each of the G2 ‘control’ and ‘JA’ parental treatment lineages, seeds from a single seed head were weighed individually, surface sterilized (0.5% sodium hypochlorite wash) and germinated on 0.8% agar plates. After 10 d, seedlings were transplanted to individual pots and grown under climate chamber conditions as described above in fully randomized blocks. Each block contained two G3 plants from each of the eight JA (J) and control (C) parental lineages; one of these two plants received a JA treatment (JJ or CJ, depending on parental lineage) and the other a mock treatment (JC and CC) (two parental treatments × two experimental treatments × eight independent replicates = 32 plants per block; see Fig. 1 for an overview of the experimental design). JA was applied to 8‐wk‐old plants by distributing 0.25 ml of a 10 mM JA solution (see above) over the surface of two standardized leaves. Mock‐treated plants received 0.25 ml of a similar ethanol/Triton‐X solution without JA. In one block of plants, 3 h after treatment, two standardized leaves (younger than the JA‐treated leaves) were collected, discarding the latex‐rich mid‐vein, flash frozen in liquid nitrogen and stored at −80°C for subsequent RNA analysis. In a second block of 32 plants, leaf tissue was sampled in a similar way (but including the mid‐vein) 24 h after treatment for subsequent leaf chemical analysis; these samples were flash frozen in liquid nitrogen, freeze dried and stored at −80°C. Three additional blocks of 32 plants were grown for time‐series reverse transcription‐quantitative polymerase chain reaction (RT‐qPCR) gene expression analysis of a known JA early response gene (*LOX2*) to validate the induced JA response (see Supporting Information Notes S1). It should be noted that, for chemical and RT‐qPCR expression analysis, all available replicate plants were used but, for RNA‐seq expression analysis, only six replicates per group were used (see below).

### RNA‐seq expression analysis

#### RNA isolation

Total RNA was isolated from liquid nitrogen‐ground tissue using Trizol (Ambion, Life Technologies, Carlsbad, CA, USA) according to the manufacturer's protocol, with an additional chloroform phase separation. Quality and concentration were checked on agarose gels and on a NanoDrop 2000 spectrophotometer (Thermo Scientific, Waltham, MA, USA). For each sample, 10 μg of total RNA was DNase treated using the Turbo DNA‐free kit (Ambion, Life Technologies). Quality and concentration were checked again on agarose gels and the NanoDrop spectrophotometer, and samples were stored at −80°C until further use.

#### RNA‐seq library preparation

Based on RNA quality, six samples from each of the four experimental groups (CC, CJ, JC, JJ; see Fig. 1) were prepared and barcoded individually using the TruSeq RNA Sample Prep kit v.2 with 24 available barcodes from index sets A and B (Illumina, San Diego, CA, USA; cat. nos. RS‐122‐2001 and RS‐122‐2002). Before sample preparation, we added 92 synthetic ERCC RNA spike‐in control sequences (Jiang *et al*., 2011) (Ambion, Life Technologies, catalog number 4456739) at 50% of the manufacturer's recommended concentration. Samples were quantified (Kapa Biosystems, Wilmington, MA, USA; cat. no. KK4824) and tested using PCR primers of Ambion ERCC controls (Life Technologies, cat. no. 4456739). Two ERCC controls of low concentration (ERCC 85 and ERCC 28) and two ERCC controls of high concentration (ERCC 130 and ERCC 4) were amplified and the cycle numbers were compared. All samples showed a qualitative difference between low and high spikes. Samples were pooled and run as a single multiplexed library on the Illumina Hiseq with one lane at Florida State University (HiSeq 2000, single end 101 bp) and two lanes at Wageningen University (HiSeq 2000, paired end 101 bp). After demultiplexing, it appeared that one sample (of the CJ group) was represented by very few reads, and this sample was excluded from further analysis. The remaining samples were examined for quality using the ERCC controls. Plots of the expected concentration vs the read count for each sample (Fig. S1) and Bland–Altman (BA) plots (Bland & Altman, 1986) between samples (Fig. S2) showed high‐quality libraries, supporting quantitative interpretation of sequence read output.

#### *De novo* transcriptome assembly

Raw Fastq files were de‐multiplexed and adapters were trimmed using Fastq‐mcf (v.488 with default settings) from Ea‐utils (Aronesty, 2011), which trims adapters and filters reads based on a minimum phred score of 20. In addition, the first 10 nucleotides of all reads (both forward and reverse) were trimmed using Seqtk (https://github.com/lh3/seqtk), because this has been shown to improve the assembly of full‐length transcripts (van Gurp *et al*., 2013). Overlapping paired‐end reads were merged using Fastq‐join from Ea‐utils (Aronesty, 2011). *De novo* transcriptome assembly was performed using Trinity v.trinityrnaseq‐r2013‐02‐16 (Haas *et al*., 2013) using default settings. The final assembly (Dryad Digital Repository: https://doi.org/10.5061/dryad.b15tr) contained 192 951 contigs (unique Trinity comp_c_seq combinations) with minimum, median, mean and maximum lengths of 200, 809, 1107 and 17 258 bp, respectively. The contigs clustered into 77 530 putative genes (unique Trinity comp_c combinations). All 192 951 contigs were mapped to the reference proteome of eudicots (NCBI RefSeq) consisting of 1 312 075 reference proteins using uBlastx in Usearch v.6.0.307 (minimum *E*‐value of 1e‐5); this algorithm has similar sensitivity to NCBI Blastx, but is much faster (Edgar, 2010). Command line output parameters were set to default, except for the output format, which was set as ‘‐userfields query + target + thi + bits + raw + evalue + qlo + qhi + tlo + thi + qframe + tframe +ids + gaps + alnlen + qrow + trow + pv + ql’, in order to obtain a tabular file that was subsequently converted to an xml input file as required by Blast2go (Conesa *et al*., 2005). Blast2go was used to perform annotations. A maximum of 20 top Blast hits (with *E*‐value < 1e‐5) was retained per contig with associated gene ontology (GO) terms as determined by Blast2go. Within Blast2go, Interproscan was run, for which results were obtained for a subset of 110 016 contigs. GO terms were derived in Blast2go based on both the Blast hits as well as the Interpro results. We observed that different contigs (comp_c_seq combinations) that belonged to the same putative gene (comp_c combination) did not always produce matching annotations, which indicates that pooling contigs for an analysis at the putative gene level would introduce an unknown amount of error because of imperfect assignment of contigs to genes. Rather than working with this unknown level of uncertainty, we decided to analyze at the contig level. Although it probably carries a multiple testing penalty, this allows for more certain interpretation of the significant results.

#### Differential gene expression analysis

As mapping algorithms are greedy, all contigs were used as the reference for alignments. Samples were aligned using Bowtie (Langmead *et al*., 2009) with the following settings: –best, –tryhard, –strata, ‐a, ‐v 3 and Last (Kielbasa *et al*., 2011) with the ‐l 25 setting. Several normalization strategies were evaluated (Dillies *et al*., 2013) using BA plots of the ERCC controls. The log(RPKM) (reads per kilobase per million mapped reads) was selected as its related BA plots were the most consistent amongst all replicates (Fig. S2). Contigs were retained for quantitative analysis if they were expressed at an average of at least 10 reads per nucleotide in all four experimental groups (CC, CJ, JC, JJ) and were at least 500 nucleotides long (*n *=* *65 827). Across all samples, this set of analyzed contigs had an average read coverage per nucleotide of 84.1 per individual sample (median 25.3). Application of the 10× coverage criterion to each of the experimental groups enables robust statistical analysis using linear models and discarded 49 658 contigs that had low expression in *all* of the experimental groups. This approach also excluded 11 766 contigs that showed no or low expression (< 10×) in some treatments but not in all treatments. Although we do not provide statistical evidence for treatment effects in these 11 766 contigs, this set may include contigs that are downregulated in response to treatment in one or more of the experimental groups (see Table S1 for the list of 11 766 contigs).

Normalized expression estimates were modeled using the following model: *Y*_*ij*_ = μ + *t*_*i*_ **+ ε_*ij*_. where *i* = (CC, CJ, JC, JJ) and *j* = (1,…, 6). *ε*_*i*_ were assumed as ~*N*(0,σ^2^_*i*_) (Law *et al*., 2014). Initial model fits with a common variance assumption did not satisfy model assumptions of residuals. The *F* test of the null hypothesis of homoscedastic error was rejected for 43% of the contigs at a false discovery rate (FDR) = 0.05 and 62% of the contigs at FDR = 0.20. In addition, the bayesian information criterion (BIC) for the homoscedastic model was worse than BIC for the heteroscedastic model 100% of the time. Thus, we fitted heteroscedastic models, for each contig separately, that allowed for different error variances for each experimental group. Individual contrasts were conducted to test the effect of parental JA treatment whilst controlling for the current (experimental) JA treatment (H_o_: μ_CC_ − μ_JC_ = 0; H_o_: μ_CJ_ − μ_JJ_ = 0) and to test the effect of the current JA treatment controlling for the parent treatment (H_o_: μ_CC_ − μ_CJ_ = 0; H_o_: μ_JJ_ − μ_JC_ = 0). Additional contrasts for the interaction between the parental and current JA treatment (H_o_: μ_CC_ − μ_CJ_ = μ_JJ_ − μ_JC_) and the effect of parent treatment on current JA response (H_o_: μ_CC_ − μ_JJ_ = 0; H_o_: μ_JC_ − μ_CJ_ = 0) were conducted. All 65 827 contrasts were simultaneously corrected for false discovery (Storey & Tibshirani, 2003). Unless otherwise specified, we consider an FDR of 0.10 to be significant. The results were qualitatively similar at FDR = 0.05 and FDR = 0.20. We selected FDR = 0.10 as a balance between type I and type II errors (Verhoeven *et al*., 2005). Results were merged with annotation, and enrichment tests were performed using annotation from Blast2go (Conesa *et al*., 2005). Because the number of differentially expressed genes was relatively low at the FDR = 0.10 significance threshold, we performed GO enrichment tests based on a significance threshold for individual transcripts at FDR = 0.20. This relaxed significance threshold results in a larger set of significant genes whilst maintaining the expected proportion of false positive results < 20%, potentially allowing for more robust enrichment analysis. Fisher's exact enrichment tests were carried out at the putative gene level, pooling for each putative gene all unique GO annotations associated with its underlying contigs and comparing the list of significant putative genes with the list of all genes analyzed.

### Untargeted metabolomic profiling

#### LC‐MS analysis

Twenty milligrams of the freeze‐dried and finely ground leaf material were extracted with 200 μl of methanol, followed by a second extraction with 200 μl of 20% methanol containing 0.1% formic acid. Both supernatants were combined and dried in a vacuum concentrator. Pellets were re‐dissolved in 60 μl of 20% methanol with 0.1% formic acid, 5 μl of which were injected onto the analytical column. For LC‐MS analysis, a Synapt G2 mass spectrometer equipped with an Acquity UPLC (Waters, Milford, MA, USA) was used. Chromatography was performed with a flow rate of 200 μl min^–1^ on a Waters Acquity C18 HSS T3 column, 2.1 × 100 mm, 1.8 μm. A 10‐min gradient from 99% water to 100% methanol (both solvents with 0.1% formic acid) was used to separate the different compounds. For ionization, positive and negative electrospray ionization modes were used. The mass spectrometer was operated in MS and MS^E^ modes in parallel with a scan range from *m/z* 50–2000. Extraction and alignment of the raw data were carried out using Waters MarkerLynx software.

#### Data analysis

Peak intensities were normalized to a total intensity of 10 000 per sample and filtered to include only mass signals present in five or more samples. To analyze differences between treatment groups, principal component analysis (PCA) and partial least squares‐discriminant analysis (PLS‐DA) on normalized data were performed in SIMCA 13.0.3. PLS‐DA models were cross‐validated with permutation tests (999 permutations). To select *m/z* values for further identification, we followed a two‐step approach. First, we performed orthogonal partial least squares‐discriminant analyses (OPLS‐DA) to obtain S‐plots and visually selected mass signals that showed the clearest association with JA treatment (either parental or direct JA treatment). All the (O)PLS‐DA models showed evidence of overfitting: *R* ^2^ and *Q* ^2^ of permuted data were not different from *R* ^2^ and *Q* ^2^ of real data (full model negative mode *Q* ^2^ = 0.16, CV‐ANOVA *P *=* *0.12; positive mode *Q* ^2^ = 0.19, CV‐ANOVA *P *=* *0.15). We therefore considered evidence from S‐plots as suggestive, but not as conclusive, for the detection of associations between mass signals and treatment.

Second, each mass signal was modeled using an ANOVA, testing the effects of direct JA treatment, parental JA treatment and the direct JA × parental JA interaction on mass signal scores. Normalized mass signals were ln‐transformed before this analysis. *P* values were subjected to FDR correction for multiple testing (across all *P* values from all model factors simultaneously) and were considered to be significant at FDR = 0.1. Only mass signals were considered for which at least three samples were present in the filtered dataset (normalized signal > 0) in each of the four experimental groups, and for which analysis of model residuals showed that residuals did not deviate significantly from a normal distribution (Shapiro–Wilk test *P *>* *0.05).

We combined evidence from the visual OPLS‐DA S‐plots and ANOVA statistical testing approaches. We report as the subset of signals with high‐confidence treatment effects those mass signals that were identified in both approaches. The putative identification of these relevant metabolites was based on mass spectra and molecular formula.

### Total phenolics assay

Total phenolics concentration was quantified using a Folin–Denis‐based protocol as described elsewhere (Engelkes *et al*., 2008). Briefly, freeze‐dried samples were ground to a fine powder and phenolics were extracted in 50% aqueous methanol at 90°C for 2 h. The total phenolic concentration was determined by exposing the samples to Folin–Denis reagent, and subsequently quantified spectrophotometrically at 750 nm by comparing the absorbance with a tannic acid calibration curve. Concentrations were expressed as tannic acid equivalent per gram of dried sample. All leaf samples were quantified in two independent replicates, whose phenolic content estimates were averaged for subsequent ANOVA to test for effects of JA treatment in the parental and experimental generations. The initial seed weight of experimental plants was included in the model as a covariate to correct for effects of initial size on the concentration of phenolics.

### Data deposition

Dandelion leaf chemical data (LC‐MS peak intensity signals and total phenolics) and the *de novo* assembled dandelion transcriptome are deposited in the Dryad digital repository (https://doi.org/10.5061/dryad.b15tr).

Dandelion RNA‐seq reads are deposited in the NCBI Sequence Read Archive (BioProject accession no. PRJNA316842; samples SRS2047454–SRS2047475).

## Results

### Effects of direct JA exposure on gene expression

**Figure 2:**
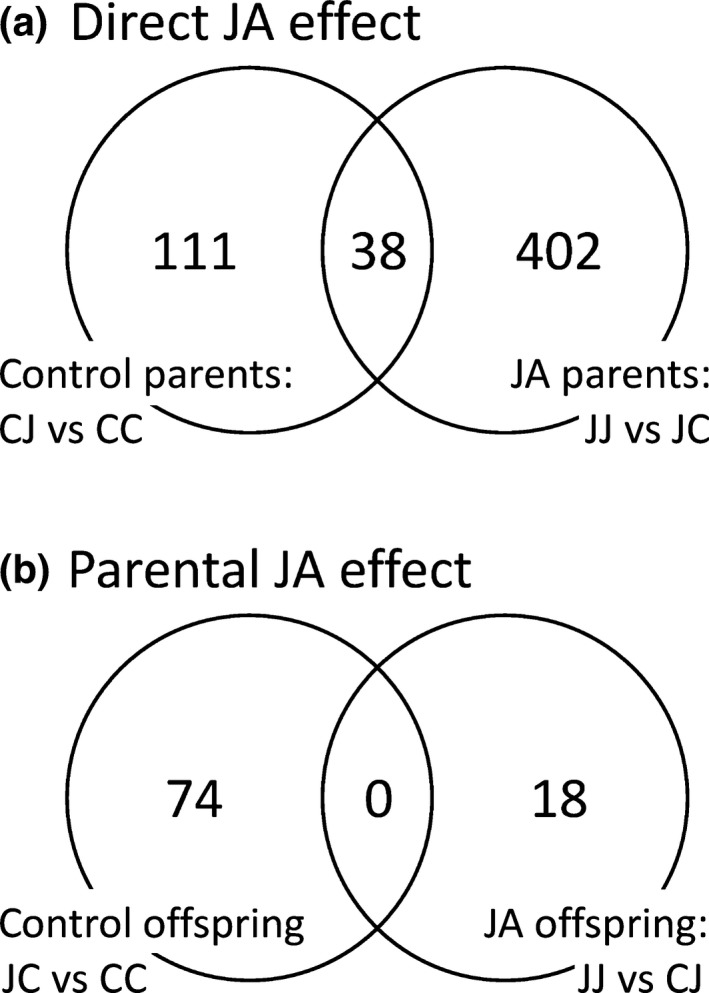
Number of differentially expressed contigs in offspring plants as a result of jasmonic acid (JA) treatment in either the offspring generation (a; direct JA effect) or parental generation (b; parental JA effect) in common dandelion. CC, CJ, JC and JJ denote the four experimental groups, in which the first letter is the parental treatment (J = JA, C = control) and the second letter is the treatment of the experimental (offspring) plants. Differential expression was tested in a priori contrasts that control for parental treatment when testing the direct JA effect, or for offspring treatment when testing the parental JA effect (significance threshold FDR = 0.1).

RT‐qPCR expression analysis of the JA early response gene *LOX2* confirmed that the experimental JA treatment elicited a systemic response that was detectable in the tissue of leaves that had not themselves been exposed to JA (see Notes S1). In offspring of control parents, RNA‐seq analysis detected 149 contigs that were differentially expressed as a result of direct JA exposure; in offspring of JA‐treated parents, 440 contigs were differentially expressed as a result of direct JA exposure (Fig. 2a; see Table S2 for all RNA‐seq test results). Thirty‐eight contigs responded to direct JA exposure irrespective of parental treatment and, accounting for this overlap, a total of 551 unique contigs showed a direct JA effect when controlling for parental treatment. Most expression differences observed were upregulations caused by JA treatment (*c*. 70% of affected contigs, Table S2). The 551 contigs clustered into 244 putative genes. At a more relaxed significance threshold of FDR = 0.20, we detected a total of 1519 significant contigs, clustering into 664 putative genes, and enrichment analysis revealed that this set of 664 differentially expressed genes was significantly enriched for GO terms associated with JA responses (Table 1). Considering only the most specific GO terms, 24 terms were significantly enriched, including biological processes related to JA biosynthesis: ‘Response to wounding’, ‘Response to other organisms’, ‘Response to host immune responses’, ‘Pathogen‐associated induction of host innate immune response’ and ‘Response to JA’ (Table 1). This confirms that our RNA‐seq approach was successful in capturing the elicited JA effect.

**Table 1:** Gene ontology (GO) terms enriched amongst the jasmonic acid (JA)‐responsive genes in common dandelion (most specific GO terms only)

GO ID	GO term	Type^a^	Number of genes		*P* value^d^
			Sign.^b^	Ref.^c^	
1. Direct JA treatment: significant JJ–JC and/or CJ–CC contrasts					
GO:0009695	Jasmonic acid biosynthetic process	P	14	23	2.0^−10^
GO:0009611	Response to wounding	P	44	379	9.2^−8^
GO:0042401	Cellular biogenic amine biosynthetic process	P	17	78	7.0^−7^
GO:0006571	Tyrosine biosynthetic process	P	14	52	8.0^−7^
GO:0009094	l‐Phenylalanine biosynthetic process	P	14	52	8.0^−7^
GO:0000162	Tryptophan biosynthetic process	P	15	63	1.2^−6^
GO:0072329	Monocarboxylic acid catabolic process	P	12	44	4.4^−6^
GO:0009423	Chorismate biosynthetic process	P	4	1	1.8^−5^
GO:0006635	Fatty acid beta‐oxidation	P	9	27	1.9^−5^
GO:0051707	Response to other organism	P	92	1291	3.6^−5^
GO:0050660	Flavin adenine dinucleotide binding	F	9	31	4.6^−5^
GO:0009821	Alkaloid biosynthetic process	P	6	11	5.7^−5^
GO:0005783	Endoplasmic reticulum	C	51	609	7.9^−5^
GO:0080167	Response to karrikin	P	29	294	2.6^−4^
GO:0004190	Aspartic‐type endopeptidase activity	F	6	16	2.9^−4^
GO:0008970	Phosphatidylcholine 1‐acylhydrolase activity	F	3	1	3.3^−4^
GO:0052572	Response to host immune response	P	6	17	3.7^−4^
GO:0034976	Response to endoplasmic reticulum stress	P	6	17	3.7^−4^
GO:0052033	Pathogen‐associated molecular pattern dependent induction by symbiont of host innate immune response	P	6	17	3.7^−4^
GO:0030433	ER‐associated ubiquitin‐dependent protein catabolic process	P	4	5	3.9^−4^
GO:0005777	Peroxisome	C	29	304	5.5^−4^
GO:0009415	Response to water	P	40	482	6.2^−4^
GO:0001676	Long‐chain fatty acid metabolic process	P	4	6	6.3^−4^
GO:0009753	Response to jasmonic acid	P	31	336	6.3^−4^
2. Parental JA treatment: significant JJ–CJ and/or JC–CC contrasts					
No significant enrichment of GO terms detected					

For this GO enrichment analysis, genes were considered to be differentially expressed at an FDR = 0.20 significance threshold.

a C, cellular component; F, molecular function; P, biological process.

b Significant gene set for direct JA effect: 1519 significant contigs at FDR 0.20 = 664 putative genes, 526 of which with GO annotation were included in the enrichment analysis. Significant gene set for parental JA effect: 451 significant contigs at FDR 0.20 = 173 putative genes, 133 of which with GO annotation were included in the enrichment analysis.

c Reference gene set consists of 11 961 analyzed and GO‐annotated putative genes.

d Two‐sided Fisher's exact test; all *P*‐values are significant after FDR control at 0.05.

### Effects of parental JA exposure on gene expression

When controlling for experimental treatment in offspring plants, 18 and 74 contigs were differentially expressed as a result of parental JA exposure in offspring groups that received JA or control treatment, respectively (Fig. 2b). Two additional contrasts, CC vs JJ and CJ vs JC, captured joint effects of direct JA treatment and parental JA treatment and were significant for 190 and 237 contigs (Table S2). In total, across the entire experimental design, 858 different contigs were differentially expressed between groups, 551 of which were detected as direct JA effects and the remaining 307 were detected only when also taking the parental treatment into account.

**Figure 3:**
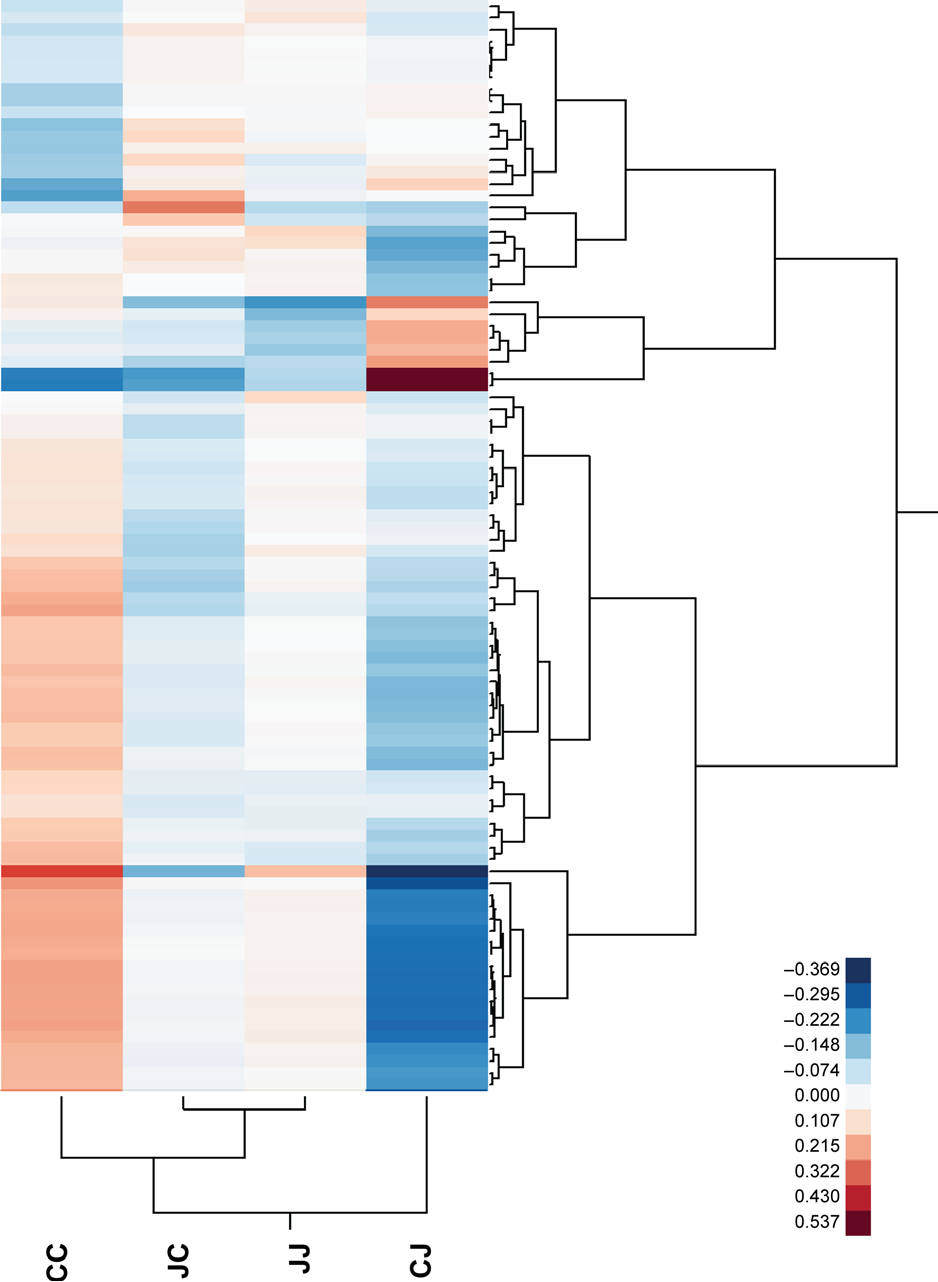
Hierarchical clustering of experimental groups and contigs based on expression scores of contigs that are differentially expressed in offspring as a result of parental jasmonic acid (JA) treatment in common dandelion. CC, CJ, JC and JJ denote the four experimental groups, in which the first letter is the parental treatment (J = JA, C = control) and the second letter is the treatment of the experimental (offspring) plants. The analysis includes 92 contigs with significant JJ–CJ contrast test and/or JC–CC contrast test (FDR = 0.1). Expression scores are log(RPKM) values averaged across replicate plants within experimental groups.

Hierarchical clustering of the 92 contigs that were differentially expressed in the same offspring environment as a result of different parental treatments indicates that the expression of these genes in JC plants is more similar to that in JJ plants than to that in CC plants (Fig. 3). This is consistent with direct JA effects that are sustained into the offspring generation. However, only five contigs (which clustered into three putative genes) overlapped between the genes that were significantly affected as a result of direct JA treatment and parental JA treatment (Table S2).

At a more relaxed significance threshold of FDR = 0.20, 451 differentially expressed contigs were detected after parental JA treatment, clustering into 173 putative genes. No significant enrichment of GO terms was observed amongst this set of 173 genes. Lack of significant GO term enrichment may be caused by the low number of significant genes. However, the list of top Blast hits for the contigs that showed a significant parental JA effect (Table S2) indicated several genes that are consistent with reported JA responses or plant defense function, such as genes associated with phosphoinositide signaling (two different inositol phosphate kinases; Sheard *et al*., 2010; Laxalt & Munnik, 2002), defense‐associated fatty acid epoxidation (CYP77A; Sauveplane *et al*., 2009), receptor‐like serine threonine kinases (often involved in pathogen recognition and defense signaling; Afzal *et al*., 2008), an ethylene‐responsive transcription factor (involved in pathogen defenses and the integration of hormonal signaling under stress; Müller & Munné‐Bosch, 2015) and respiratory burst oxidases (Torres & Dangl, 2005).

### Effects of JA treatment on between‐replicate variation in expression

**Figure 4:**
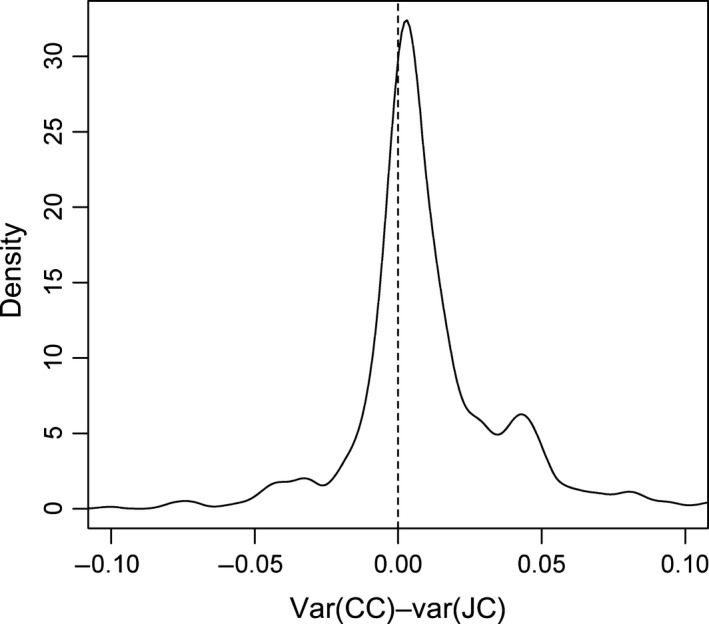
Between‐plant variance in gene expression is reduced, not increased, in common dandelion plants whose parents received jasmonic acid (JA) treatment. Density distribution of the difference in variance between CC and JC groups, based on the subset of contigs (43.8% of the total) with significant heteroscedasticity amongst the four experimental groups. CC and JC denote the experimental groups in which CC received control treatment in the parental as well as in the experimental (offspring) generation, and JC received parental JA treatment and offspring control treatment.

Forty‐four per cent of all analyzed contigs showed significant differences in variances between the four experimental groups (CC, CJ, JC, JJ), as indicated by a significant difference in the estimated variances (Folded *F*, FDR = 0.05), and all models showed an improved fit based on the BIC when the group‐specific error variances were included compared with models that assumed a common error. In these contigs with significant heteroscedasticity, almost always the CJ group had the highest variance (96.3% of contigs, see Table S2). Although there is a difference in sample size (*n *=* *5 in the CJ group and *n *=* *6 in the other groups), this suggests that JA treatment leads to large between‐replicate variation in gene expression 3 h after treatment. However, no trend was observed indicating that JA treatment of parents leads to increased variance in offspring gene expression. Indeed, the estimated variance of expression for the CC group was larger than the estimated variance for the JC group in the large majority of contigs with significant heteroscedasticity (Fig. 4).

### Effects of direct and parental JA exposure on leaf chemical composition

**Figure 5:**
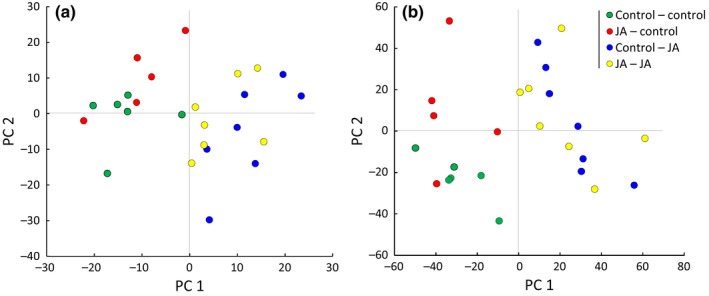
LC‐MS metabolomics analysis of leaf tissue from common dandelion plants sampled 24 h after jasmonic acid (JA) treatment (blue and yellow dots) or 24 h after control treatment (green and red dots), and whose parents had received either JA treatment (yellow and red dots) or control treatment (blue and green dots). (a) Principal component analysis (PCA) clustering based on mass signals of the LC‐MS negative ion mode; axes 1 and 2 explain 14% and 11% of the variation, respectively. (b) PCA clustering based on LC‐MS positive mode; axes 1 and 2 explain 13% and 9% of the variation, respectively.

Untargeted LC‐MS metabolomics profiling detected, on average, 968 mass signals per sample (in total 1210 across all samples) in the negative ionization mode and an average of 5151 mass signals per sample (in total 7728 across all samples) in the positive ionization mode (data accessible at Dryad Digital Repository: https://doi.org/10.5061/dryad.b15tr). The two ion modes show a slightly different selectivity based on the propensity of a molecule to gain or lose a proton. For example, phenolic compounds are detected well in the negative mode, whereas N‐based metabolites, such as alkaloids, are generally better detected in the positive mode. In both modes, PCAs clearly separated samples based on the direct JA treatment 24 h before tissue sampling (CJ and JJ vs CC and JC, Fig. 5). An effect caused by parental JA treatment was visible: CC and JC samples clustered with only limited overlap (red vs green dots, Fig. 5). Such separation based on parental treatment was not observed in plants that received JA treatment 24 h before sampling (i.e. CJ and JJ, Fig. 5). ANOVA also indicated a strong induction of the leaf metabolome by JA treatment, where *c*. 16% of the tested mass signals showed a significant effect of direct JA treatment (Table 2). In addition, parental JA treatment had a significant effect (either as a main effect or in interaction with experimental treatment, Table 2) in 1.6% of mass signals.

**Table 2:** Number of LC‐MS mass signals affected by direct and parental jasmonic acid (JA) treatment in common dandelion (ANOVA, significance threshold FDR = 0.1)

	No. mass signals
Total	8938
ANOVA tested^a^	2821
Significant direct JA effect	463 (16.4%)
Significant parental JA effect	31 (1.10%)
Significant direct × parental JA interaction	15 (0.53%)

a At least three non‐zero observations in each of the four experimental groups, and normally distributed residuals.

Based on a visual inspection of S‐plots from OPLS‐DA, we selected 33 mass signals as potentially associated with direct JA treatment and/or with parental JA treatment (Fig. S3). Of these, 16 were also significant in the ANOVA tests and six of these overlapping results could be putatively assigned to known compounds (Table S3). Based on these putative assignments, the experimental JA treatment response involved changes in linolenic acid, caftaric acid, phosphatidylglycerol and phosphatidylinositol. A response to parental JA treatment was detected in phosphatidylinositol and glycosylated malonic acid (Table S3). Mass signals that were selected in the OPLS‐DA S‐plots that showed an effect of JA, but not significant in the ANOVA test, included putative assignments to caftaric acid, chicoric acid, phosphatidylcholine and a glycosylated flavone.

**Figure 6:**
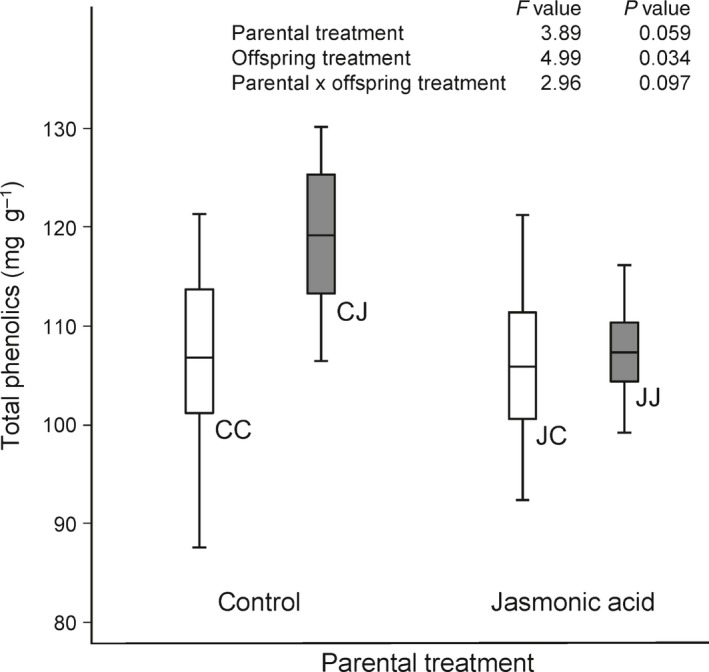
Leaf total phenolics concentration in offspring of control plants and offspring of jasmonic acid (JA)‐treated plants, 24 h after exposure to JA (dark gray box plots) or mock treatment (white box plots) in common dandelion. CC, CJ, JC and JJ denote the four experimental groups in which the first letter is the parental treatment (J = JA, C = control) and the second letter is the treatment of the experimental (offspring) plants. Boxes and whiskers denote the 25^th^–75^th^ percentile and minimum–maximum observations, respectively; group mean values are indicated by the horizontal line. The inset table shows ANOVA test results from a model that also accounted for possible plant size effects as a result of differences in initial seed weight.

When looking at a specific class of compounds with known anti‐herbivore and anti‐microbial responses, total phenolics, an effect of parental JA treatment was observed (Fig. 6). In offspring of control parents, JA treatment increased the concentration of leaf phenolics within 24 h. However, the phenolic concentration did not reach the same level on JA treatment in offspring of JA‐treated parents (Fig. 6). Although the interaction between parental and offspring JA treatment was not significant (0.05 < *P *<* *0.1, see Fig. 6), this suggests an inhibition of JA inducibility of phenolics in offspring after parental JA treatment.

## Discussion

Although parental environmental effects are well documented in plants, the extent to which molecular phenotypes are affected by environmental exposures in previous generations is largely unknown. Very few transcriptome‐wide evaluations have been performed and results have been ambiguous, ranging from complete absence (Molinier *et al*., 2006) to very widespread effects (Colicchio *et al*., 2015) after parental exposure to cues associated with pathogen or herbivore attack. Our study provides evidence that the inherited effect of parental exposure on molecular phenotypes can be substantial, and supports a previously noted trend that transgenerational effects of herbivore and pathogen attack may be a widespread phenomenon in plants (Holeski *et al*., 2012). The effect that we observed on offspring after parental JA treatment is consistent with functions related to the treatment, with both transcriptomics and metabolomics analysis converging on phosphatidylinositol signaling as a transgenerationally affected pathway. More generally, our results suggest that the interpretation of gene expression and other molecular studies needs to be mindful of effects on the seed source.

Gene expression analysis revealed a clear functional response to direct JA treatment which is consistent with known JA‐induced processes (Table 1; Notes S1). Because we putatively identified only a modest number of JA‐affected chemical compounds, a detailed pathway analysis based on the chemical JA response was not possible. However, qualitative evaluation of the compounds that were putatively identified indicated that the functional signal identified in the gene expression data was mirrored in the metabolomics data. Compounds that were JA induced included the precursor of JA biosynthesis (linolenic acid; Wasternack & Hause, 2013; consistent with the detected JA treatment effect on the JA biosynthesis pathway). Also identified were the hydrocinnamate phenolics caftaric acid and chicoric acid. These are major phenolic compounds in species from the Compositae family (Cheminat *et al*., 1988; Oh *et al*., 2009), including dandelion (Schutz *et al*., 2005), that are thought to function in plant defenses against pathogens and herbivores (Lee & Scagel, 2013). In dandelion, phenolic inositol esters, triterpene acetates and a sesquiterpene lactone taraxinic acid ester are important secondary metabolites in latex (Huber *et al*., 2015). The JA response of the hydrocinnamate phenolics in our experiment, which matches the pattern observed in total phenolics (Fig. 6), suggests that these phenolics are inducible secondary metabolites involved in herbivore defenses in dandelion.

An important result of our study is that there is also a functional signal in the *inherited* JA response. Several of the 40 putative genes that showed a significant parental JA effect have known functions in JA‐ or defense‐related processes. Strikingly, the gene expression and metabolomics analyses converged on one pathway that was affected by parental JA treatment: the phosphatidylinositol signaling pathway. Two of the identified genes with parental JA effect were phosphatidylinositol phosphate kinases, and one of only two parental JA effect metabolites that could be putatively assigned was phosphatidylinositol. Thus, JA treatment had a durable effect on phosphatidylinositol signaling. Phosphatidylinositol phosphate kinases are enzymes that phosphorylate the precursor inositol in a pathway that produces various phosphatidylinositol phosphates and inositol phosphates, which subsequently act as intracellular second messengers on perceiving an extracellular signal (Munnik & Testerink, 2009). Enzymes in this pathway are inducible by stress and plant hormone treatments (Lin *et al*., 2004), and play an important role in defense response signaling on herbivore and pathogen attack (Laxalt & Munnik, 2002; Mosblech *et al*., 2008; Hung *et al*., 2014). Phospholipid signaling is involved in various aspects of biotic defense signaling, including JA biosynthesis by affecting linoleic acid production from plasma membranes, potentiation of the COI1–JAZ complex for jasmonate recognition via a specific inositol phosphate cofactor and intracellular signaling to activate and later downregulate defense gene expression after pathogen elicitor recognition (Laxalt & Munnik, 2002; Sheard *et al*., 2010; Zhang & Xiao, 2015). Although the exact role of this pathway after JA treatment in dandelion remains to be determined, our congruent RNA‐seq and LC‐MS results provide a clear starting point for future work to pinpoint the (epigenetic) mechanisms that enable a JA response to persist across generations.

Genes that showed a significant effect of parental JA treatment were largely different from genes that showed an expression response on JA treatment in the experimental generation. This is counter to the idea that gene expression in offspring is limited to a sustained activity pattern in a subset of JA‐responsive genes (Bruce *et al*., 2007). However, this is perhaps an overly simplistic view when the timing of the JA‐induced expression response is considered. On JA application, a rapid succession of transcriptional regulatory programs unfolds with different genes being involved in the immediate, intermediate and long‐term responses (Acosta & Farmer, 2010; Wasternack & Hause, 2013). In our experiment, we tested the early expression response 3 h after JA treatment, which involves mostly different genes, and is poorly correlated with the expression response that is observed at later stages (Tytgat *et al*., 2013). A subset of the later stage genes may correspond to the genes that are still affected in offspring. An alternative explanation for the lack of overlap of differentially expressed genes after JA treatment vs after parental JA treatment could be related to the low statistical power to detect differentially expressed genes. If only a modest subset of genes that are affected by the treatments are recognized as statistically significant, then limited overlap between the two sets of detected genes is expected, even when many of the genes are in fact affected by both treatments.

It has been proposed that exposure to stressful environments can trigger enhanced variability amongst offspring individuals, rather than a mean shift in trait values or gene expression levels (Rapp & Wendel, 2005; Verhoeven *et al*., 2010; Herman *et al*., 2014). Enhanced variability, which is potentially mediated by an increased rate of transgenerationally stable epigenetic mutations, might reflect a bet‐hedging strategy that increases the probability of at least some progeny surviving or maintaining high fitness. In our experiment, JA treatment increased variability in gene expression 3 h after induction, which may reflect subtle timing differences in the early JA response between replicated plants. However, increased variance was not sustained into the offspring generation. By contrast, gene expression amongst offspring of JA‐treated plants showed reduced variance more often than increased variance compared with the offspring of control plants. This is perhaps a result of a conditioning of the response and a reduction in stochastic expression. Such apparent canalization of gene expression after parental JA treatment is interesting and suggests that there is some constraint on expression changes that does not exist in the control plants. Reduced variability in gene expression amongst offspring individuals might also be related to crosstalk between plant defense pathways. For instance, transgenerational priming of salicylic acid (SA)‐related plant defenses after parental treatment with SA or with other hormones or inducing agents (Luna *et al*., 2012; Slaughter *et al*., 2012) can enhance offspring expression of SA‐related genes, but, at the same time, because of crosstalk between JA and SA pathways, can suppress the activity of JA‐related defense responses (Luna *et al*., 2012). Thus, transgenerational activation of one pathway may result in transgenerational suppression of another pathway, and such suppression may be reflected as reduced offspring expression variability in a subset of genes.

This study revealed effects of parental treatment on the offspring transcriptome and metabolome, but the underlying mechanism of the transmission of the environmental effect between the generations remains to be elucidated. Parental environmental effects can be mediated by various mechanisms. In our experiment, parental JA treatment occurred well before flowering; thus no direct induction of the germline occurred. Possible mechanisms therefore include maternal modification of the embryonic hormone balance or inherited epigenetic effects (Boyko *et al*., 2007; Luna *et al*., 2012; Rasmann *et al*., 2012; Bond & Baulcombe, 2014), which is consistent with previous observations of JA‐induced heritable modification of DNA methylation patterns in dandelion (Verhoeven *et al*., 2010).

In conclusion, our findings demonstrate that parental environmental conditions can have long‐lasting, functional effects that are visible in the transcriptome and metabolome of offspring individuals. Our results imply that, in any gene expression study, environmental conditions should be controlled not only in the experimental generation, but also in the previous parental generation. The proportion of affected genes may be considerable, as evidenced in our study, where a single JA application in parental plants during vegetative growth, well before the induction of flowering, affected the expression of approximately one‐third of the JA‐responsive genes in offspring plants. This observation provides insight into the scope of parental environmental effects. Our results also provides a starting point for further unraveling of the underlying mechanisms that mediate transgenerational effects in plant interactions with herbivores, pathogens or parasites, where such inherited effects may be particularly common (Poulin & Thomas, 2008; Holeski *et al*., 2012).

## Author contributions

K.J.F.V. conceived and designed the study. E.H.V. performed the experiment and the phenolics analysis. M.S. and M.M. performed the metabolomics analysis, and M.M. and K.J.F.V. performed the statistical analysis of the metabolomics data. C.O. performed RNA isolations and RT‐qPCRs. C.O. and A.M.M. carried out the RNA‐seq library preparation. T.P.v.G. and J.F.d.C. performed the *de novo* transcriptome assembly and annotation. A.M.M., K.J.F.V. and L.M.M. performed the analysis of differential gene expression. K.J.F.V. and L.M.M. wrote the manuscript with input from all co‐authors. All authors read and approved the final manuscript.

## Supporting information

Please note: Wiley Blackwell are not responsible for the content or functionality of any Supporting Information supplied by the authors. Any queries (other than missing material) should be directed to the *New Phytologist* Central Office.

**Fig. S1** Observed read counts for synthetic ERCC RNA spike‐in control sequences.
**Fig. S3** Orthogonal partial least squares‐discriminant analyses (OPLS‐DA) selection of jasmonic acid (JA)‐responsive mass signals.
**Table S3** Putative identification of LC‐MS mass signals
**Notes S1** Reverse transcription‐quantitative polymerase chain reaction (RT‐qPCR) expression validation of the early jasmonic acid (JA) response candidate gene *LOX2*.

**Fig. S2** Bland–Altman plots for within‐group pairwise comparisons based on ERCC controls.

**Table S1** Contigs excluded from statistical analysis, but meeting the coverage threshold in at least one of the experimental groups

**Table S2** RNA‐seq test results for differential expression analysis
