# Supplementary material for: Intergenerational environmental effects: functional signals in offspring transcriptomes and metabolomes after parental jasmonic acid treatment in apomictic dandelion
Source: New Phytol. 2017 Oct 16;217(2):871–82. doi: 10.1111/nph.14835 (PMC5741498; doi:10.1111/nph.14835)
Supplement: Supplementary file 1 — Fig. S1 Observed read counts for synthetic ERCC RNA spike‐in control sequences. Fig. S3 Orthogonal partial least squares‐discriminant analyses (OPLS‐DA) selection of jasmonic acid (JA)‐responsive mass signals. Table S3 Putative identification of LC‐MS mass signals Notes S1 Reverse transcription‐quantitative polymerase chain reaction (RT‐qPCR) expression validation of the early jasmonic acid (JA) response candidate gene LOX2. [file NPH-217-871-s001.pdf]

***New Phytologist* Supporting Information**

Article title: Intergenerational environmental effects: Functional signals in offspring transcriptomes and metabolomes after parental jasmonic acid treatment in apomictic dandelion

Authors: Koen J.F. Verhoeven, Eline H. Verbon, Thomas P. van Gurp, Carla Oplaat, Julie Ferreira de Carvalho, Alison M. Morse, Mark Stahl, Mirka Macel and Lauren M. McIntyre

Article acceptance date: 31 August 2017

The following Supporting Information is available for this article:

Fig. S1 Observed read counts for synthetic ERCC RNA spike-in control sequences

Fig. S2 Bland-Altman plots for within-group pairwise comparisons based on ERCC controls (see separate file)

Fig. S3 OPLS-DA selection of JA-responsive mass signals

Table S1 Contigs excluded from statistical analysis, but meeting the coverage threshold in at least one of the experimental groups (see separate file)

Table S2 RNA-seq test results for differential expression analysis (see separate file)

Table S3 Putative identification of LC-MS mass signals

Notes S1 RT-qPCR expression validation of the early JA-response candidate gene LOX2

Fig. S1 Observed read counts for synthetic ERCC RNA spike-in control sequences. Read count (log(RPKM)) for 92 synthetic ERCC RNA spike-in control sequences are plotted against their expected concentration (log_Mix1_adj_conc) based on known ERCC spike-in Mix concentrations (Ambion, Life technologies, catalog number 4456739). Each panel shows results for a different library. Sample 54 was excluded from analysis. Experimental group CC: samples 2, 20, 35, 87, 103, 126; experimental group CJ: samples 4, 19, 34, 70, 81; experimental group JC: samples 142, 146, 166, 181, 217, 227; experimental group JJ: samples 145, 170, 188, 204, 229, 246.

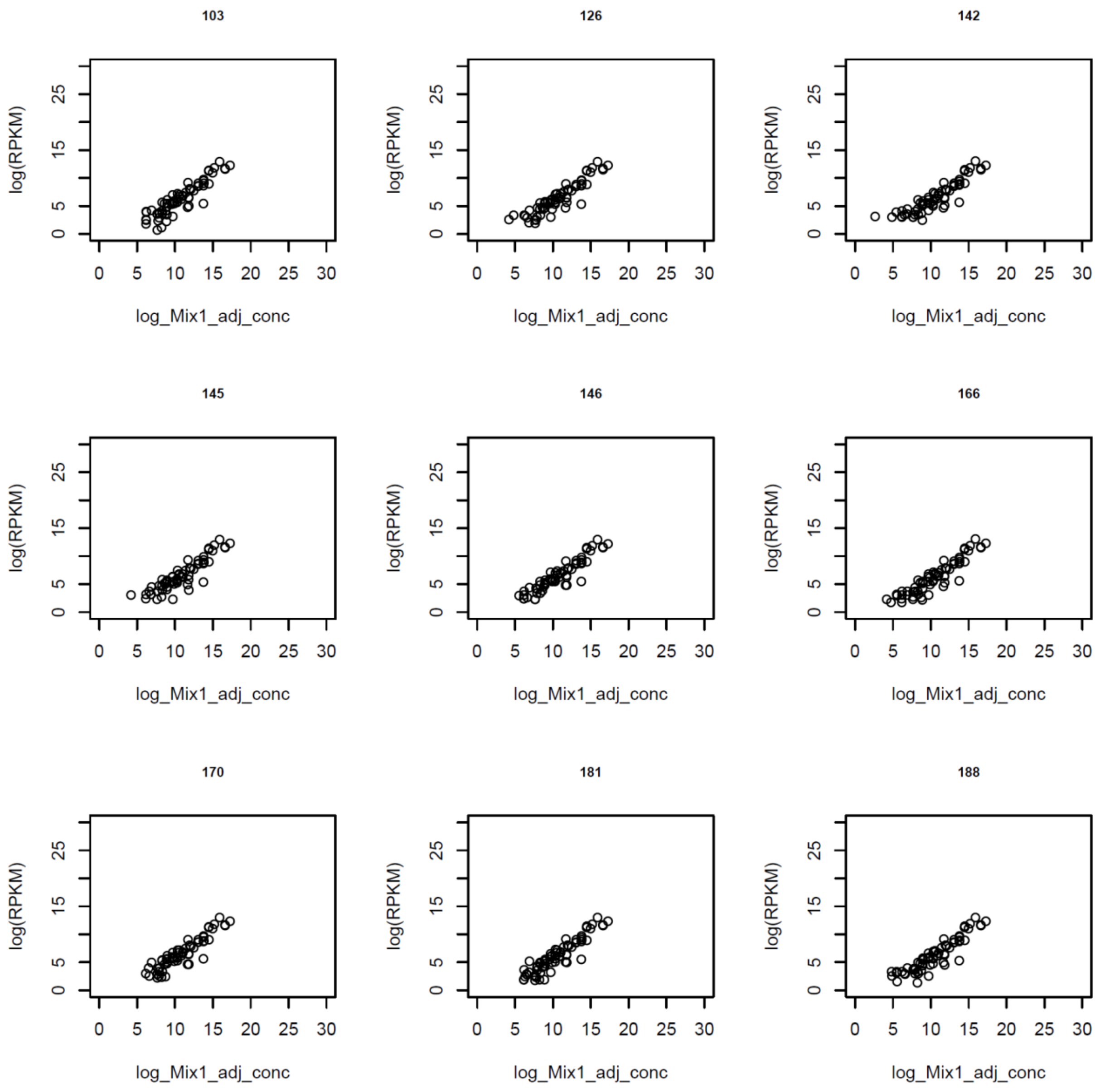

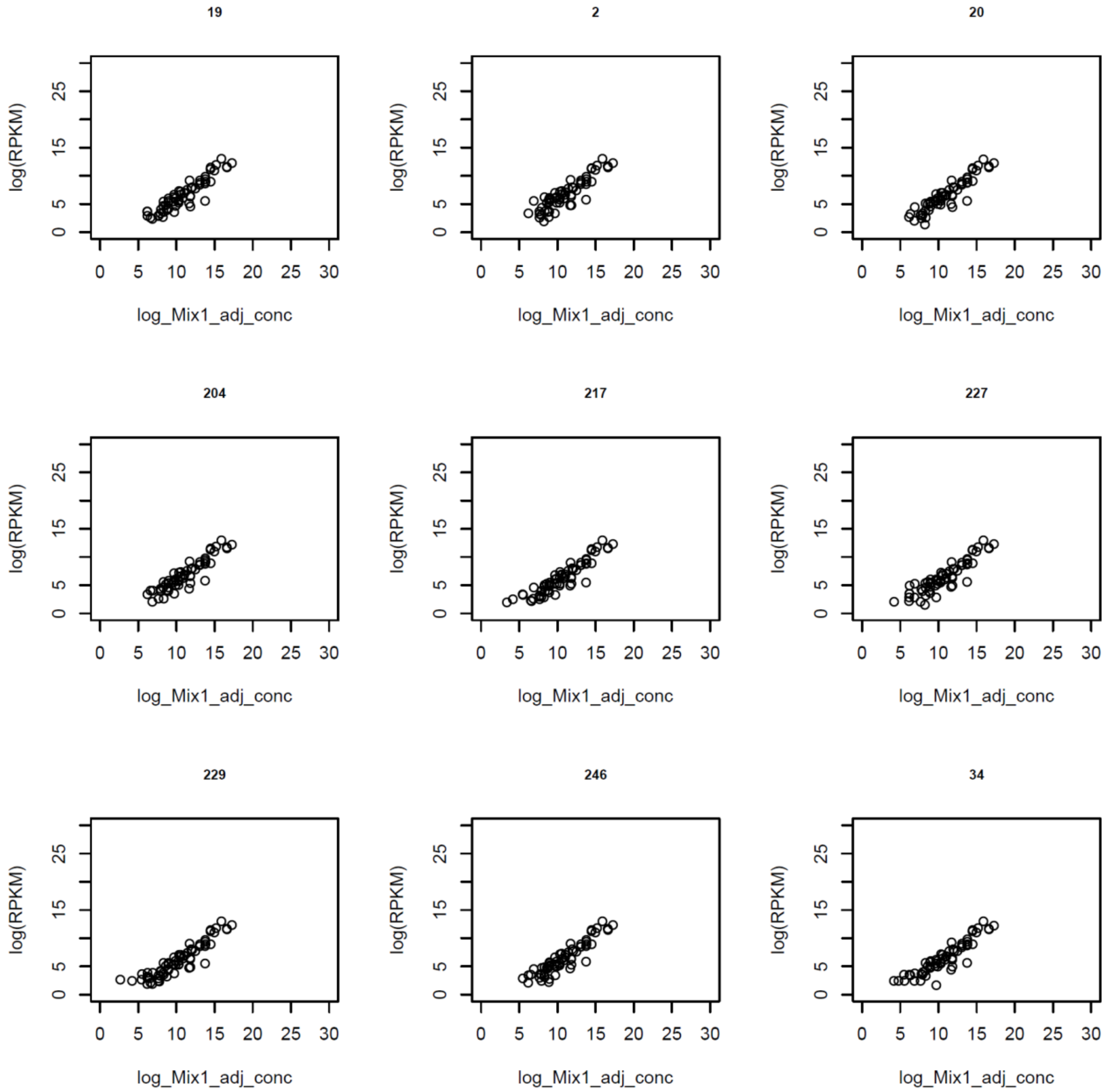

19
2
20
204
217
227
229
246
34
log(RPKM)
log_Mix1_adj_conc
0 5 15 25
0 5 10 15 20 25 30

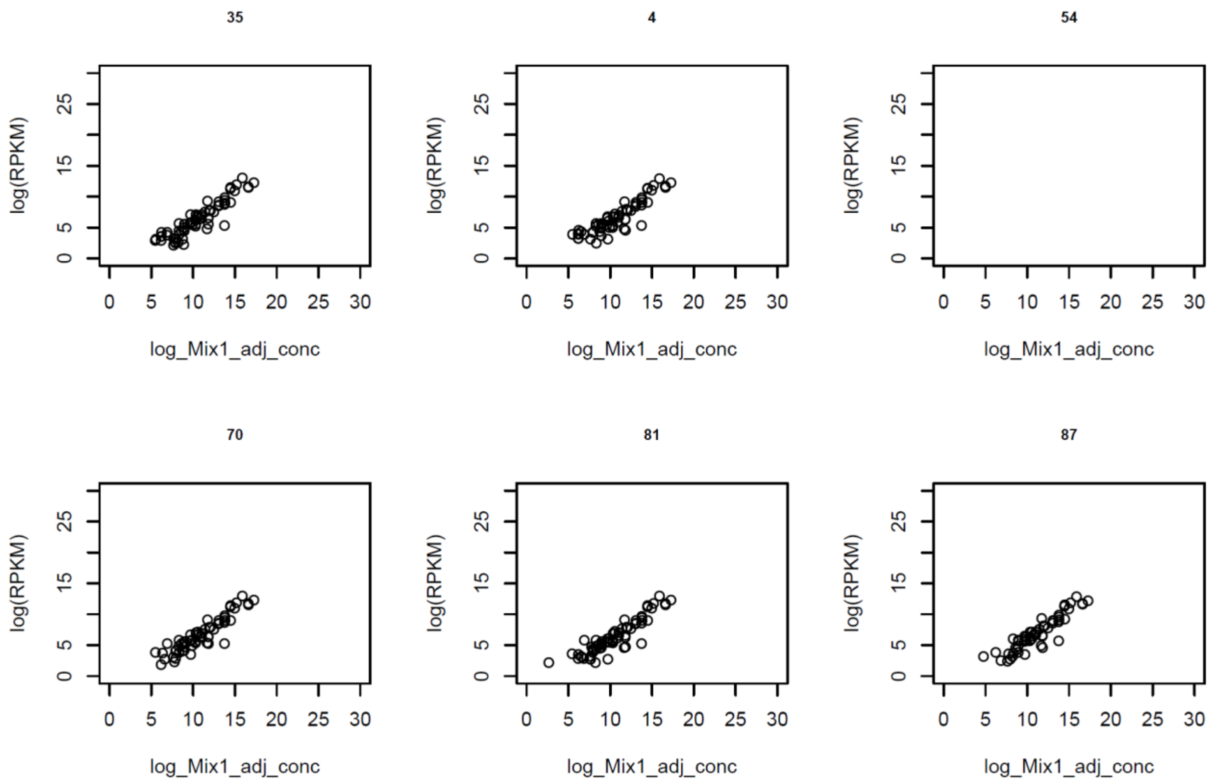
35
4
54
70
81
87
log(RPKM)
log_Mix1_adj_conc
0
5
10
15
20
25
30

Fig. S2 Bland-Altman plots for within-group pairwise comparisons based on ERCC controls. The difference in log(RPKM) values are plotted against average log(RPKM) value) for all pairwise comparisons of samples within experimental groups, based on the ERCC RNA spike-in controls. For sample codes see Figure S1.

PDF file Fig_S2 submitted separately

Fig. S3 OPLS-DA selection of JA-responsive mass signals. S-plots from OPLS-DA for detecting association with direct JA treatment (panels A, D) and with parental JA treatment (panels B, C, E, F, G). Red dots indicate mass signals that were selected visually and that passed an additional quality check based on averages, standard deviations and range of peak intensities of the underlying m/z values. Mass signals selected for association with parental JA effect were evaluated across the entire experimental design (panels B, E) as well as in specific comparisons that controlled for offspring treatments (only control offspring: panels C, F; only JA offspring: panel G, no additional mass signals were selected in the negative model for this comparison). In these specific comparisons only those mass signals were selected that were not already selected in other comparisons. Mass signal numbers correspond to Table S3.

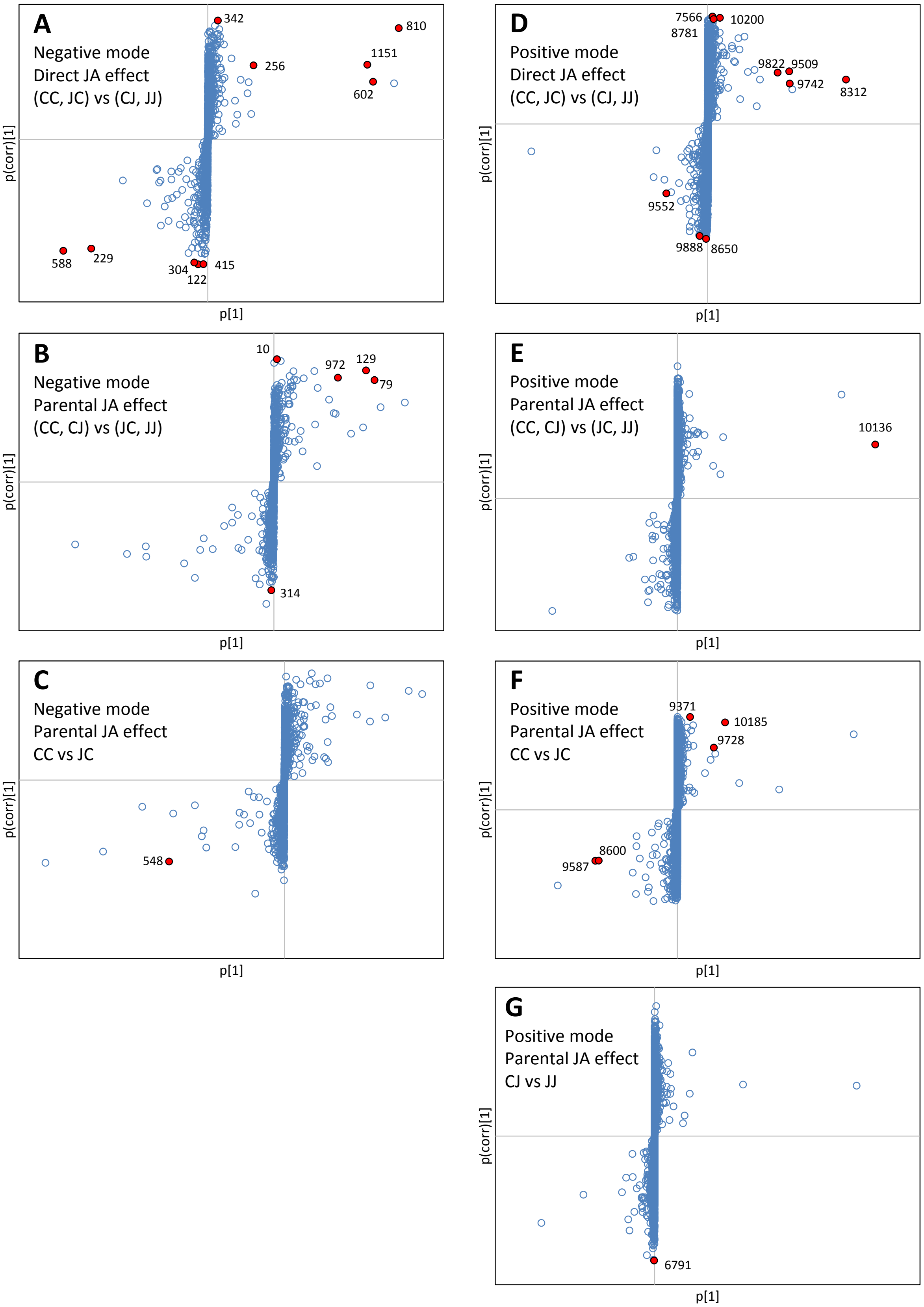
A
Negative mode
Direct JA effect
(CC, JC) vs (CJ, JJ)
342
256
810
1151
602
588
229
304
122
415
p(corr)[1]
p[1]
D
Positive mode
Direct JA effect
(CC, JC) vs (CJ, JJ)
7566
8781
10200
9822
9509
9742
8312
9552
9888
8650
p(corr)[1]
p[1]
B
Negative mode
Parental JA effect
(CC, CJ) vs (JC, JJ)
10
972
129
79
314
p(corr)[1]
p[1]
E
Positive mode
Parental JA effect
(CC, CJ) vs (JC, JJ)
10136
p(corr)[1]
p[1]
C
Negative mode
Parental JA effect
CC vs JC
548
p(corr)[1]
p[1]
F
Positive mode
Parental JA effect
CC vs JC
9371
10185
9728
8600
9587
p(corr)[1]
p[1]
G
Positive mode
Parental JA effect
CJ vs JJ
6791
p(corr)[1]
p[1]

Table S1 Contigs excluded from statistical analysis, but meeting the coverage threshold in at least one of the experimental groups

Data file Table_S1.csv submitted separately

Contigs in this table reach the coverage threshold (average of at least 10 reads per nucleotide) in at least one, but not in all, of the four experimental groups, and are not statistically analysed using linear models. For each of the experimental groups, expression is given as the mean value of log(RPKM) scores.

Table S2 RNA-seq test results for differential expression analysis

Data file Table_S2.csv submitted separately

For each tested contig in the de novo transcriptome assembly the following information is provided:

- Mean expression in each of the four experimental groups (lsmeans_log_RPKM_<group>).
- Results of differential expression tests between experimental groups, both as raw p values (contrast_rawp_<comparison>) and as FDR-corrected p values (contrast_pfdr_<comparison>).
- Flag_pfdr_JAdirect10 indicates contigs with significant effect (FDR=0.10) of direct JA treatment when controlling for parental treatment. Flag_pfdr_JAparent10 indicates contigs with significant effect (FDR=0.10) of parental JA treatment when controlling for offspring treatment.
- Variance estimates for each of the four experimental groups (variance_<group>).
- Results of heteroscedasticity tests between experimental groups, both as raw p values (foldedF_rawp_<comparison>) and as FDR-corrected p values (foldedF_pfdr_<comparison>). Flag_pfdr_heteroscedastic<FDR level> indicate contigs with significant heteroscedasticity between experimental groups at different FDR levels (0.05, 0.1, 0.2).
- uBLASTx top hit information (uBLASTx_<result>): description, accession, E value, score, alignment length, and number of positives, of the uBLASTx top hit.
- BLAST2GO_sequence_description: sequence description as determined by BLAST2GO based on annotation of the list of top uBLASTx hits (maximum 20 hits per contig).

Table S3 Putative identification of LC-MS mass signals. Mass signals in negative and positive ion modes were selected based on S-plots from OPLS-DA (see Fig. S3) and ANOVA tests. Assignments are based on mass spectra and molecular formula. Indicated are ANOVA test results for each mass signal that passed criteria for ANOVA testing. In bold are mass signals that were identified in both the OPLS-DA method and in the ANOVA testing method and that could be putatively assigned.

**LC-MS mass signal**					**ANOVA p value**[1]		**Putative assignment**
Ion mode	Mass nr.	Rt (min)	m/z (M+/-H)	effect[2]	direct JA effect	parental JA effect	
A. OPLS-DA S-plot comparison 'Direct JA effect'							
neg	122	14.4	174.955	down	<0.001	0.408	not assigned
neg	229	9.2	261.113	down	<0.001	0.920	not assigned
neg	256	14.0	277.217	up	0.014	0.867	**Linolenic acid ($C_{18}H_{29}O_2$)**
neg	304	14.4	304.913	down	<0.001	0.522	not assigned
neg	342	2.2	323.097	up	0.003	0.802	not assigned
neg	415	7.4	367.160	down	<0.001	0.767	not assigned
neg	588	9.2	469.171	down	<0.001	0.772	**likely Cinnamic aldehyde derivative ($C_{22}H_{30}O_{11}$)**
neg	602	8.6	473.072	up	0.380	0.918	Chicoric acid (Dicaffeoyltartaric acid, $C_{22}H_{17}O_{12}$)
neg	810	6.6	623.089	up	<0.001	0.858	**Caftaric acid (2M-H adduct)**
neg	1151	8.1	947.153	up	0.043	0.432	not assigned
pos	8312	7.4	163.039	up			not assigned
pos	9888	14.2	230.891	down	<0.001	0.928	not assigned
pos	8650	14.2	246.864	down	<0.001	0.708	not assigned
pos	7566	9.6	355.172	up	<0.001	0.652	not assigned
pos	9509	13.4	537.306	up			likely Rhodexin A ($C_{29}H_{45}O_9$)
pos	9552	2.2	543.132	down	0.084	0.540	not assigned
pos	10200	14.0	660.424	up			likely Phosphatidylserin ($C_{34}H_{36}NO_9P$)
pos	8781	14.5	702.470	up			Phosphatidylcholine ($C_{37}H_{69}NO_9P$)
pos	9742	14.0	780.554	up	0.198	0.927	Phosphatidylcholine ($C_{44}H_{79}NO_8P$)
pos	9822	14.1	797.518	up	0.056	0.972	**Phosphatidylglycerol ($C_{47}H_{73}O_{10}$)** or **Phosphatidylinositol ($C_{40}H_{78}O_{13}P$)**
B. OPLS-DA S-plot comparison 'Parental JA effect'							
neg	10	6.5	87.008	up	0.862	0.323	Caftaric acid fragment
neg	79	6.5	149.008	up	0.976	0.588	Caftaric acid fragment
neg	129	6.6	179.034	up	0.908	0.485	Caftaric acid fragment
neg	314	2.5	309.082	down	0.753	0.074	**likely Glycosylated malonic acid ($C_{11}H_{17}O_{10}$)**

neg	548	7.1	447.129	down	0.721	0.708	Glycosylated flavone
neg	972	13.5	741.470	up	0.067	0.321	likely Phosphatidylglycerol ($C_{40}H_{70}O_{10}P$)[3]
pos	9371	5.1	136.062	up	0.870	0.699	not assigned
pos	9587	14.1	756.554	down	0.303	0.497	Phosphatidylcholine ($C_{42}H_{79}NO_8P$)
pos	9728	7.6	780.156	up	0.451	0.867	not assigned
pos	8600	14.2	782.569	down	0.713	0.880	Phosphatidylcholine ($C_{44}H_{81}NO_8P$)
pos	6791	14.4	822.754	down	0.855	0.289	likely Phosphatidylcholine ($C_{51}H_{100}NO_6$)
pos	10136	14.3	871.574	up			likely Phosphatidylinositol ($C_{47}H_{84}O_{12}P$)
pos	10185	14.3	885.553	up	0.009	0.017	**likely Phosphatidylinositol ($C_{47}H_{82}O_{13}P$)**

[1] P values are FDR-corrected and were considered significant at FDR=0.1. Only p values are shown for mass signals that met the requirements that we posed for significance testing, i.e. all four experimental groups (CC, CJ, JC, JJ) represented by at least three non-zero observations and residuals test of deviation from normality $p > 0.05$.

[2] Direction of JA-effect on metabolite abundance: up- or down-regulated.

[3] Compound identified visually from PLS-DA S-plots based on association with parental JA treatment but with only significant direct (=experimental) JA effect in statistical testing.

Notes S1 RT-qPCR expression validation of the early JA-response candidate gene LOX2.

To independently validate the efficacy of the jasmonic acid (JA) treatment, we grew extra plants for time series expression monitoring of a well-established indicator gene of the early JA response, LOX2 (Bell et al 1993; Rasmann 2012; Wasternack 2013). Following the experimental design and growing conditions as described in the manuscript, three blocks of 32 plants were grown (2 parental treatments [JA, control] x 2 experimental treatments [JA, control] x 8 replicate plants) and leaf tissue was sampled 30 minutes after treatment (block 1), 60 minutes after treatment (block 2) and 180 minutes after treatment (block 3). Application of treatment (JA / mock induction) in the different blocks was timed such that tissue collection of all blocks was done within a 2-h time window between 11:45h – 13:45h. The below Figure A and Table A test results show a significant increase in LOX2 expression after experimental JA treatment. The initial JA response at 30 minutes seems to occur earlier in plants whose parents had also received JA treatment, but this difference was not significant in the overall statistical model (Experimental treatment x Parental treatment interaction: $p = 0.28$; Time x Experimental treatment x Parental treatment: $p = 0.36$; see Table A).

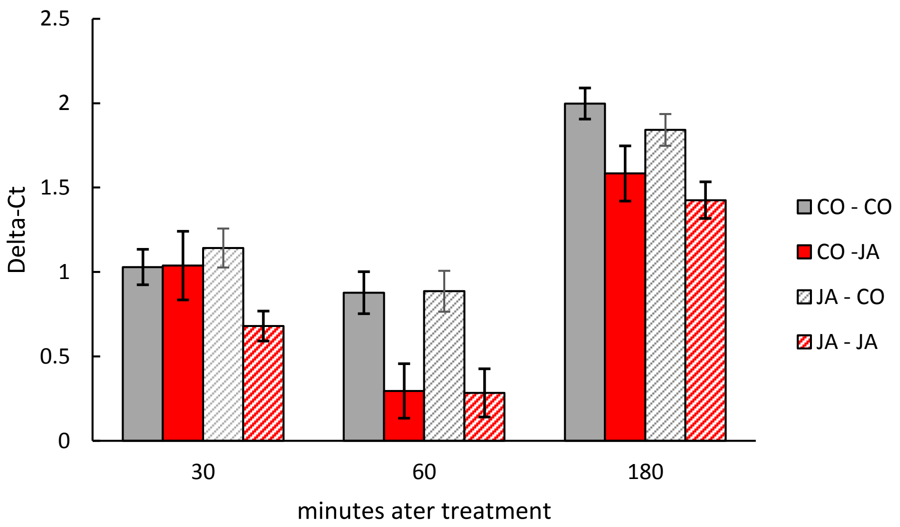

Figure A. Normalized gene expression (Delta-Ct) of LOX2 at 30, 60, and 180 minutes after experimental treatment (JA or Control) in plants whose parents had received JA or Control treatment. Groups are indicated by ‘parental treatment – experimental treatment’ (solid bars: parental control treatment; hatched bars: parental JA treatment). Note that lower Delta-Ct values correspond to higher gene expression.

Table A. Test results from 3-way ANOVA on LOX2 normalized expression values (Delta-Ct).

Model term	d.f.	F value	P value
Time	2	76.4	<.0001
Experimental treatment	1	29.59	<.0001
Parental treatment	1	1.54	0.22
Experimental treatment x Parental treatment	1	1.18	0.28
Time x Experimental treatment	2	1.94	0.15
Time x Parental treatment	2	0.39	0.68
Time x Experimental treatment x Parental treatment	2	1.03	0.36

Primers

LOX2 expression quantification was evaluated using GAPDH and EF1alpha as normalization genes. Primers for LOX2 were designed using the dandelion RNAseq data (comp68049_c0_seq1; see deposited transcriptome) and primers for GAPDH were designed based on sequences from related species and further optimized after sanger sequencing of the amplified product. Primers for dandelion EF1alpha were kindly provided by Janina Post and Prof. Dirk Prüfer (Münster University). For each primer combination we performed a 4-5point dilution series of a pooled cDNA sample to determine PCR efficiency. The final reaction had a PCR efficiency between 85% and 108%. For each primer combination the PCR product was sequenced using the fw and rv primer (on two different samples, sample one with the fw primer and sample 2 with the reverse primer) to show that the PCR product is unique and the sequence corresponds with the used comp.

gene	Fw primer 5'-3'	Reverse primer 5'-3'	PCR program
LOX2	CGTCAACCATTACTACCCAG	GTCTTTCTTGTCTGCGTGTC	2 min 95°C; 40x(5s 95°C, 30s 64°C)
EF1alpha	CGAGAGATTCGAGAAGGAAGC	CTGTGCAGTAGTACTTGGTGG	2 min 95°C; 40x(5s 95°C, 30s 60°C)
GAPDH	CGGTGTGAACGAGAAGGAAT	TCTGTGTAGCGGTGATGGAG	2 min 95°C; 40x(5s 95°C, 30s 60°C)

RT-qPCR analysis

1 µg DNase-treated total RNA was used for cDNA synthesis using the SuperScript III First-Strand Synthesis system for RT-PCR (Invitrogen, Life Technologies, the Netherlands) according to the manufacturer's protocol and using the oloigo(dT)$_{20}$ primer. qPCR reactions were performed on

the RotorGene 6000 (Qiagen, the Netherlands) using manufacturer's software Rotor-Gene Q series (Qiagen, the Netherlands). qPCR reactions were performed in a total volume of 20 ul containing: SensiFAST™ SYBR No-ROX kit (Bioline, GCbiotech, the Netherlands) , 400 nM of each primer, 5 ul 50-fold diluted cDNA template. All reactions were performed in technical duplicate, i.e. two independent dilutions of the same cDNA sample. Samples were processed in fully randomized order. Data was exported using the export for LinReg function in the Rotor-Gene Q software and analysed in LinRegPCR version 2014-7.

Data analysis

CQ values for each reaction were extracted using LINREG. Based on EF1alpha and GAPDH CQ values, a normalization index was determined using BESTKEEPER, and LOX2 normalized expression scores were calculated as the difference between CQ-LOX2 and this BESTKEEPER normalization index (Delta-Ct = CQ-LOX2 – BESTKEEPER INDEX). The BESTKEEPER index, based on CQ values for EF1alpha and GAPDH, ranged between 19.7–23.4 and was typically lower than CQ values for LOX2 which ranged between 21.1-24.7. For each biological sample, normalized expression scores of the two technical duplicates were averaged to yield a single LOX2 expression score. Effects of parental treatment (2 levels: JA, control), experimental treatment (2 levels: JA, control) and time after treatment (3 levels: 30, 60, 180 minutes) and all interactions on normalized expression scores were tested using a 3-way ANOVA (SAS 9.2, SAS Institute, Cary NC).

References

Bell, E., & Mullet, J. E. (1993). Characterization of an Arabidopsis lipoxygenase gene responsive to methyl jasmonate and wounding. *Plant Physiology*, *103*(4), 1133-1137.

Rasmann, S., De Vos, M., Casteel, C. L., Tian, D., Halitschke, R., Sun, J. Y., Agrawal, A. A., Felton, G. W., & Jander, G. (2012). Herbivory in the previous generation primes plants for enhanced insect resistance. *Plant Physiology*, *158*(2), 854-863.

Wasternack, C., & Hause, B. (2013). Jasmonates: biosynthesis, perception, signal transduction and action in plant stress response, growth and development. An update to the 2007 review in Annals of Botany. *Annals of botany*, *111*(6), 1021-1058.
