# Supplementary material for: Intergenerational environmental effects: functional signals in offspring transcriptomes and metabolomes after parental jasmonic acid treatment in apomictic dandelion
Source: New Phytol. 2017 Oct 16;217(2):871–82. doi: 10.1111/nph.14835 (PMC5741498; doi:10.1111/nph.14835)

***New Phytologist* Supporting Information : Fig. S2**

Article title: Intergenerational environmental effects: Functional signals in offspring transcriptomes and metabolomes after parental jasmonic acid treatment in apomictic dandelion

Authors: Koen J.F. Verhoeven, Eline H. Verbon, Thomas P. van Gurp, Carla Oplaat, Julie Ferreira de Carvalho, Alison M. Morse, Mark Stahl, Mirka Macel and Lauren M. McIntyre

Article acceptance date: 31 August 2017

Fig. S2 Bland-Altman plots for within-group pairwise comparisons based on ERCC controls. The difference in log(RPKM) values are plotted against average log(RPKM) value) for all pairwise comparisons of samples within experimental groups, based on the ERCC RNA spike-in controls. For sample codes see Figure S1.

2 vs 20 log_rpkm

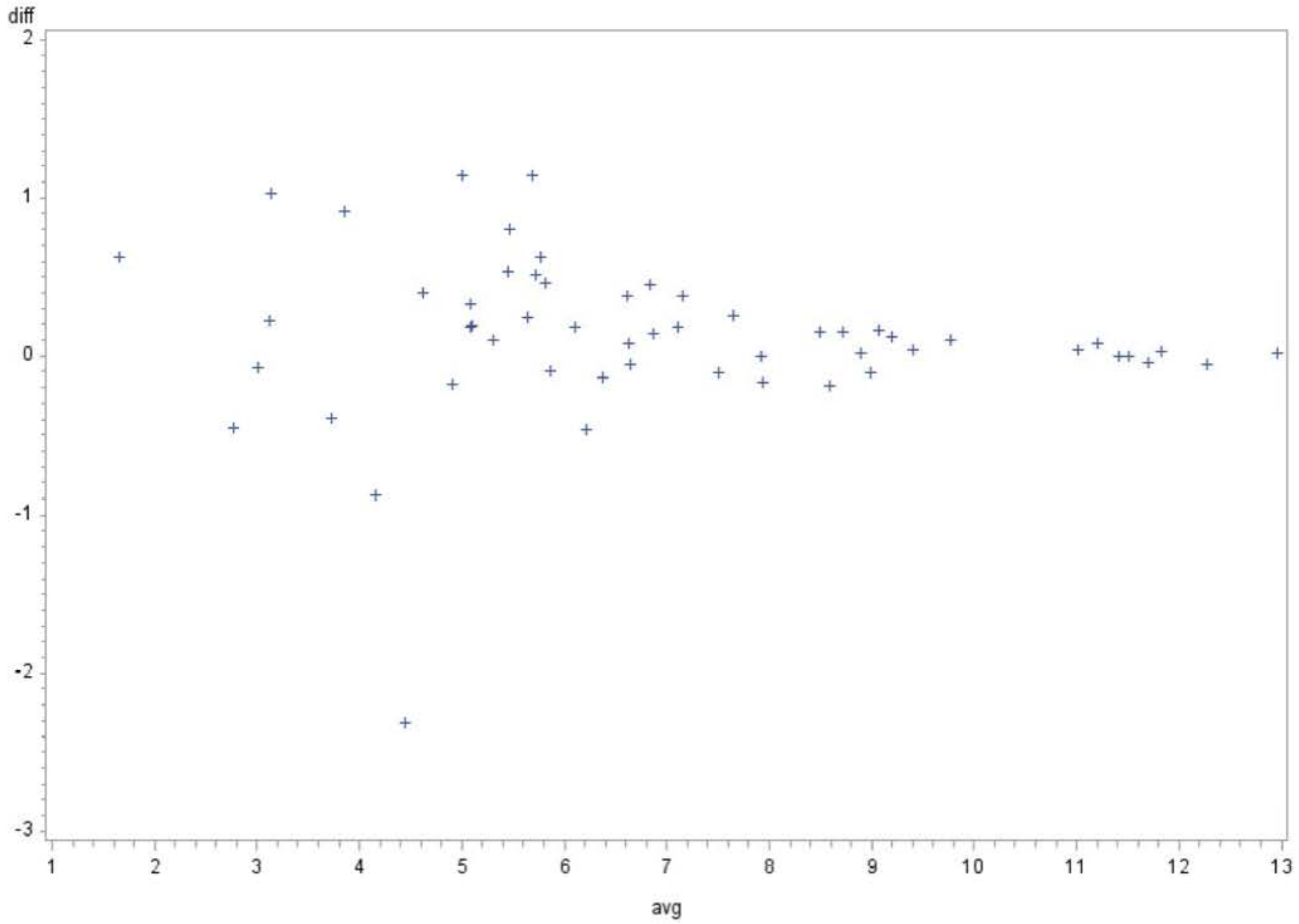

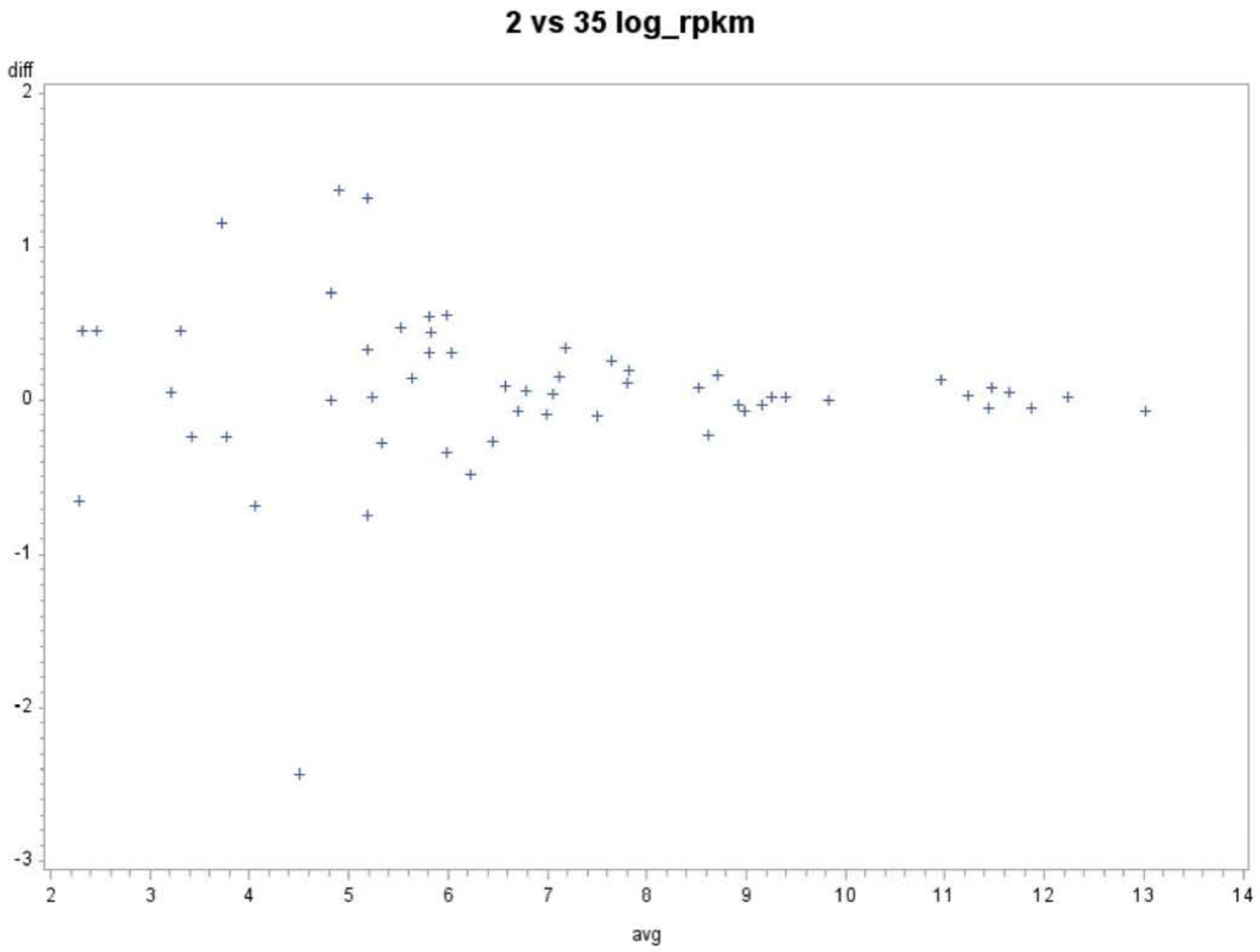
2 vs 35 log_rpkm
diff
2
1
0
-1
-2
-3
2
3
4
5
6
7
8
9
10
11
12
13
14
avg

2 vs 87 log_rpkm

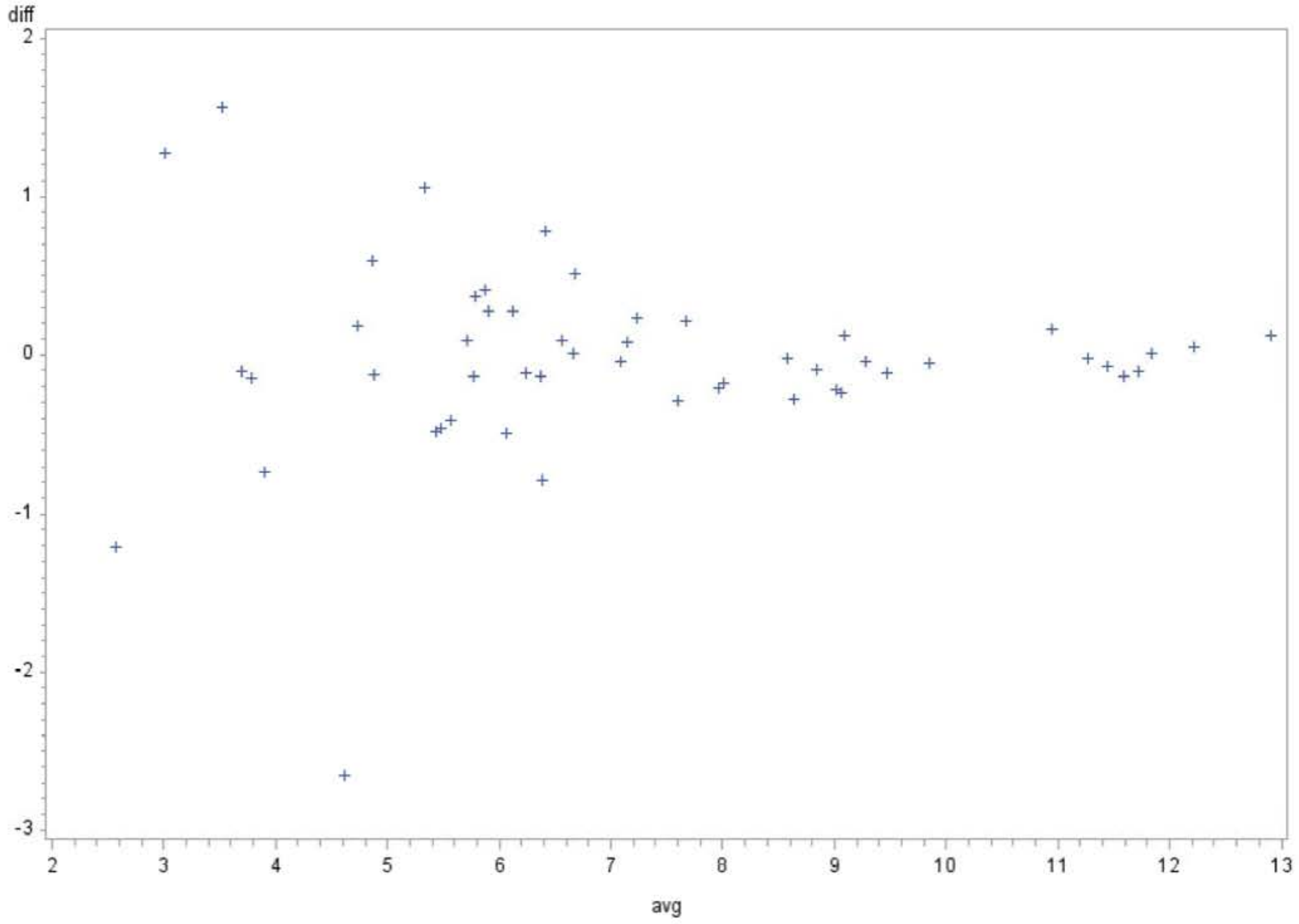

2 vs 103 log_rpkm

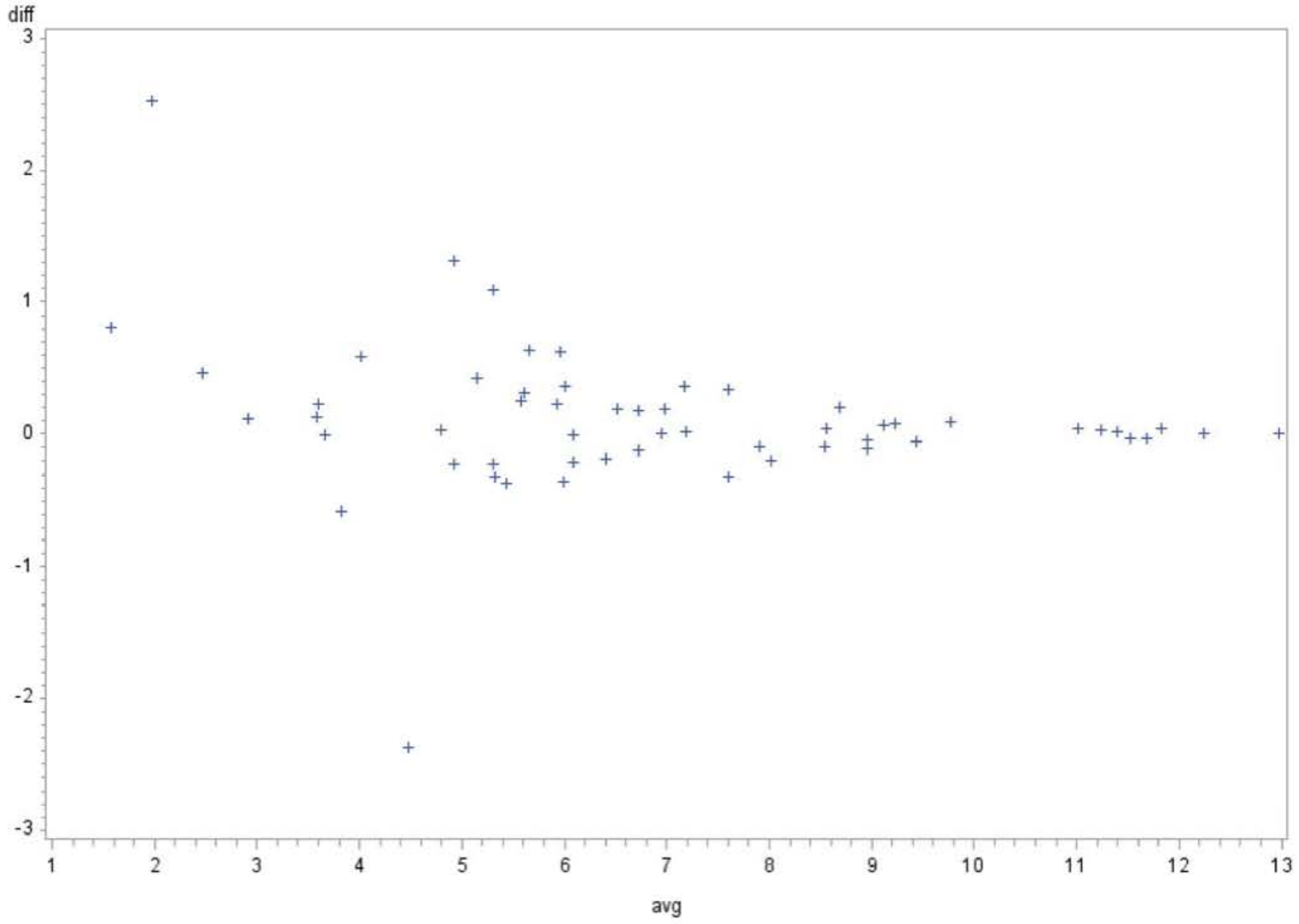

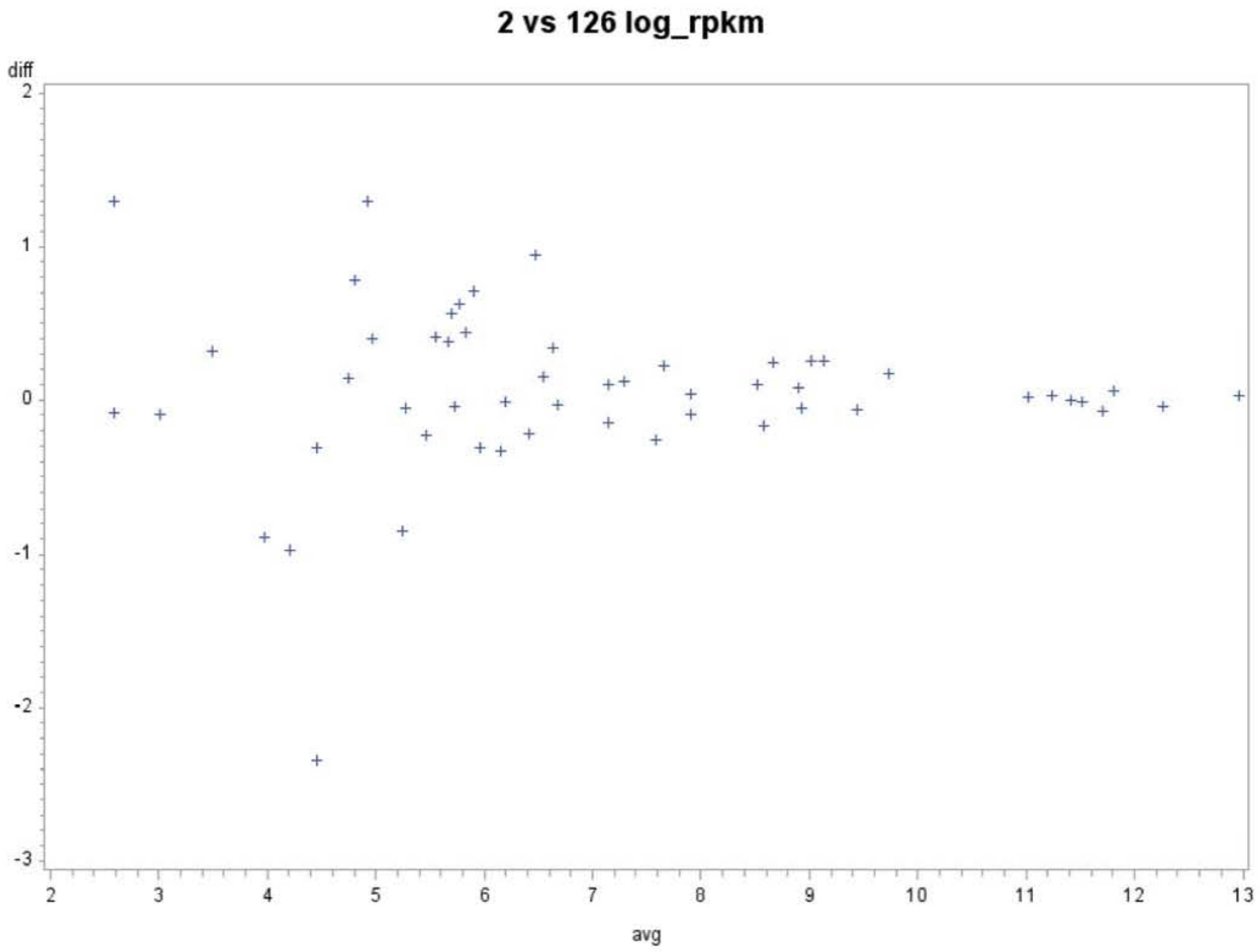
2 vs 126 log_rpkm
diff
2
1
0
-1
-2
-3
2
3
4
5
6
7
8
9
10
11
12
13
avg

20 vs 35 log_rpkm

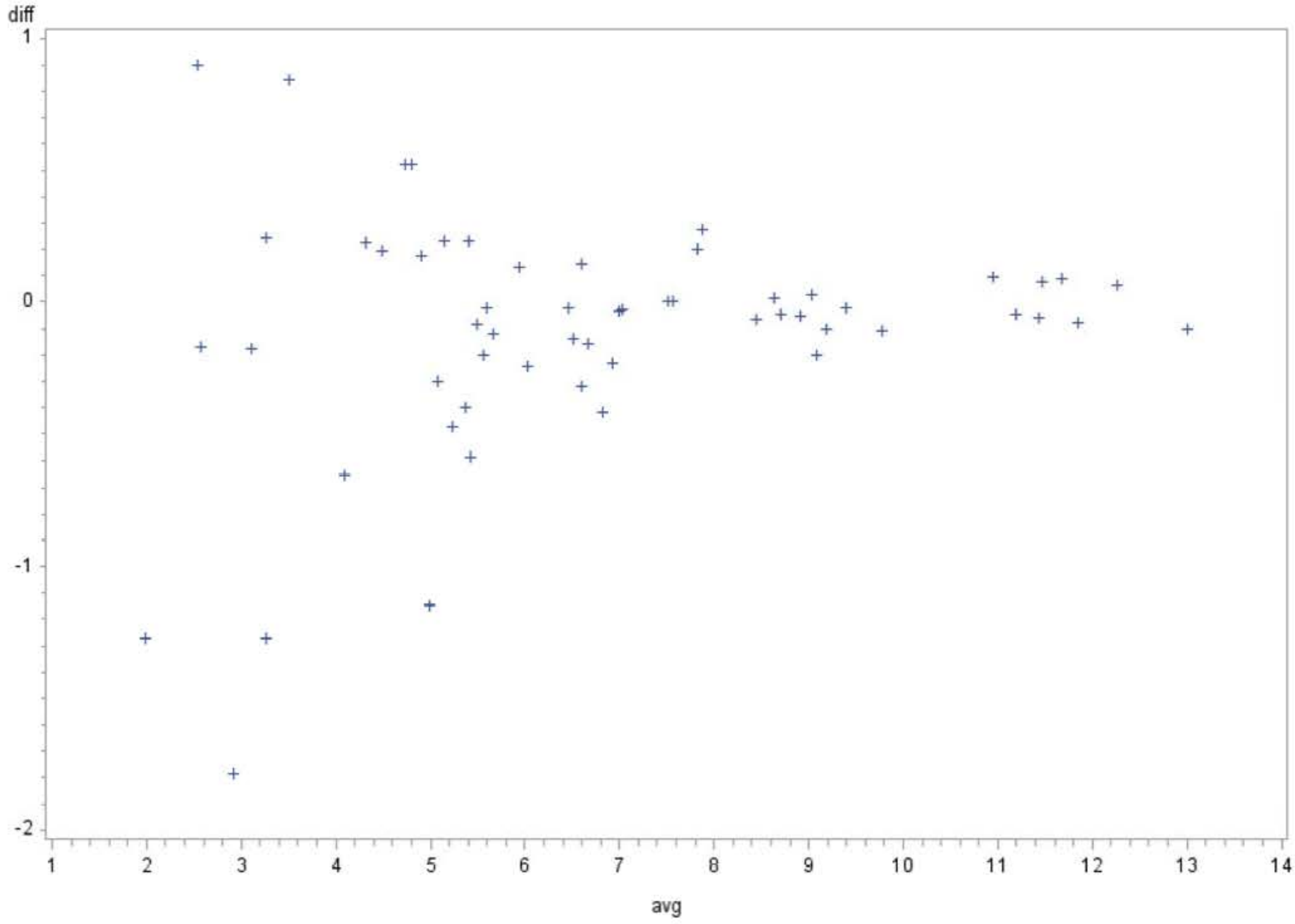

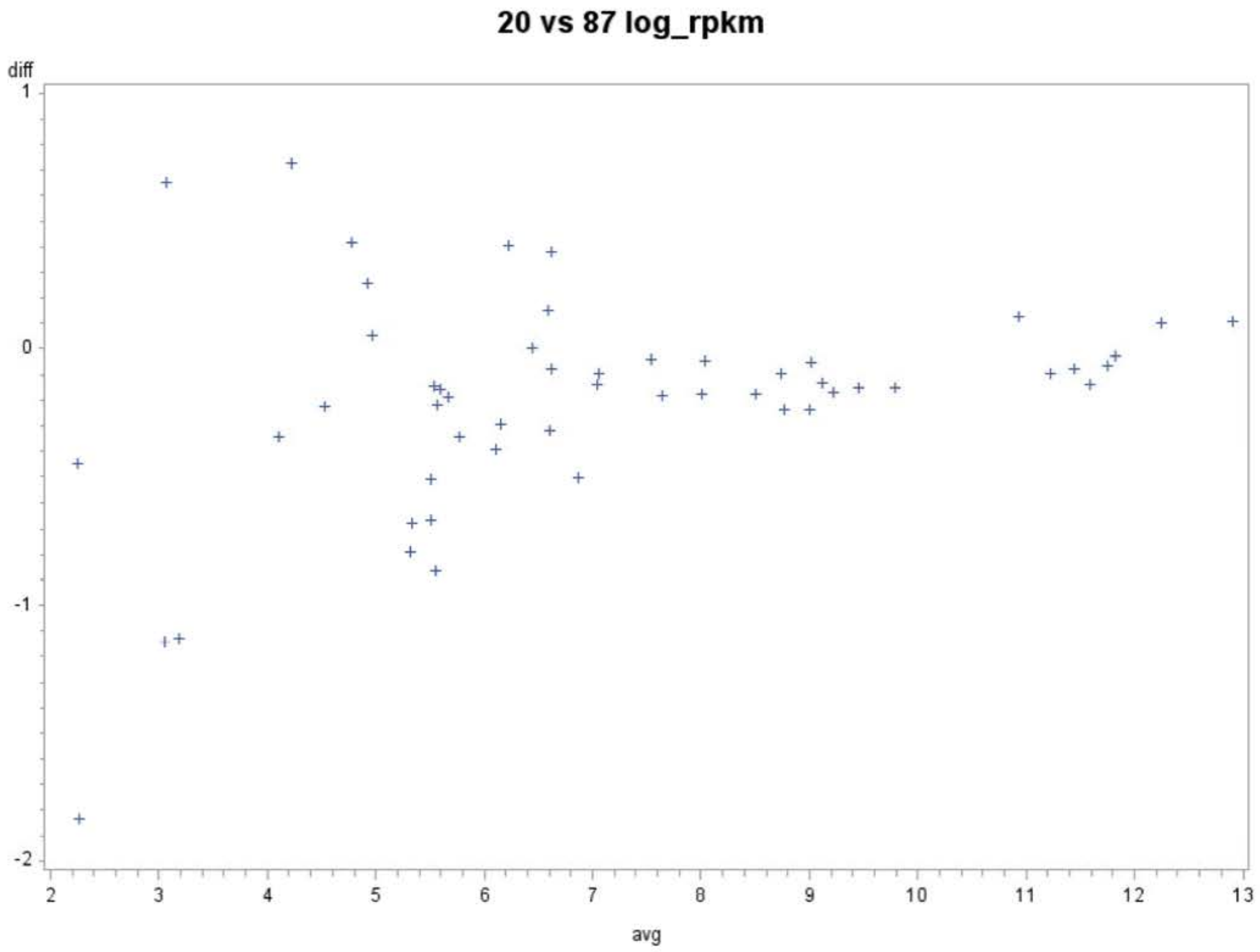

20 vs 87 log_rpkm
diff
1
0
-1
-2
2
3
4
5
6
7
8
9
10
11
12
13
avg

20 vs 103 log_rpkm

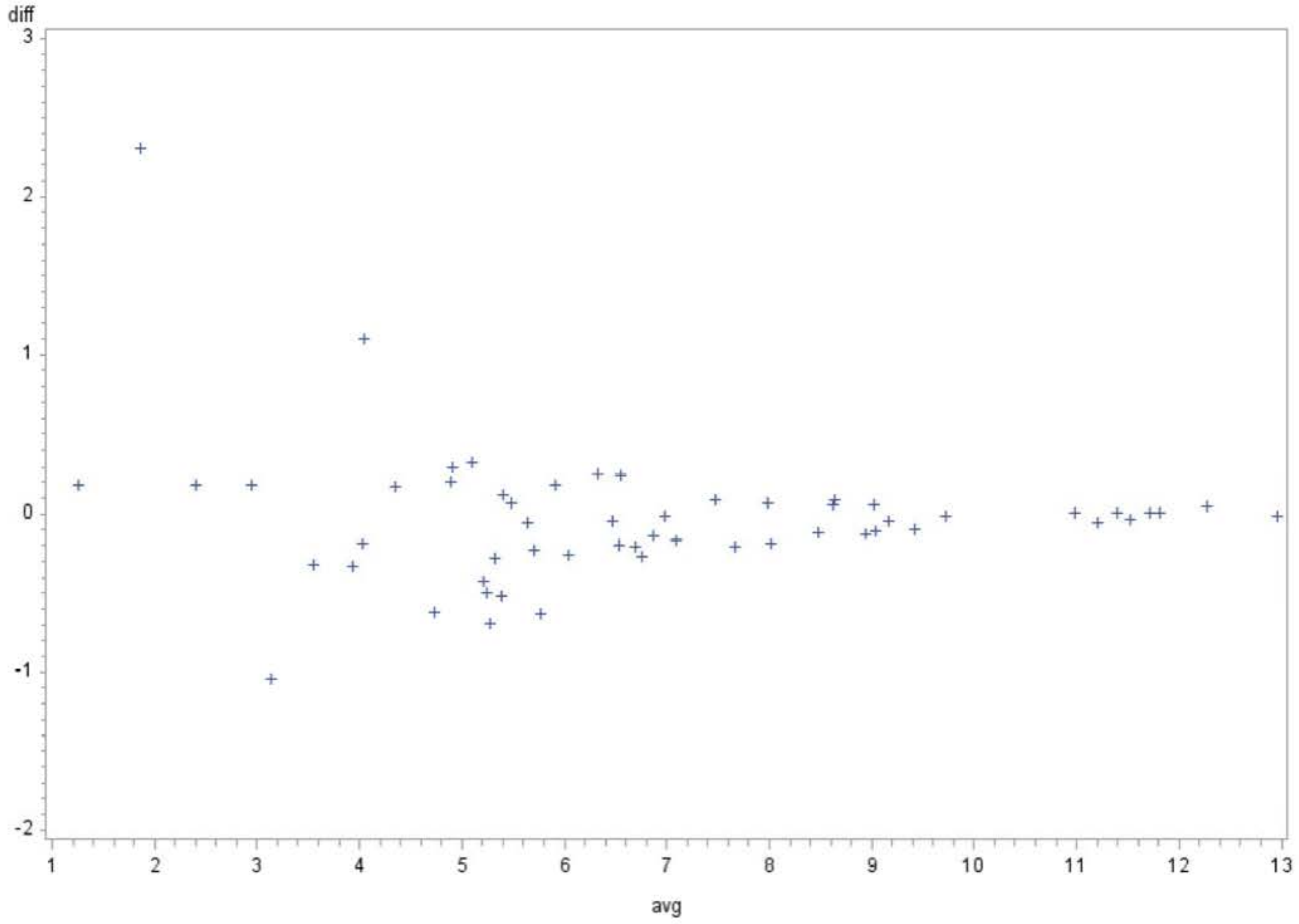

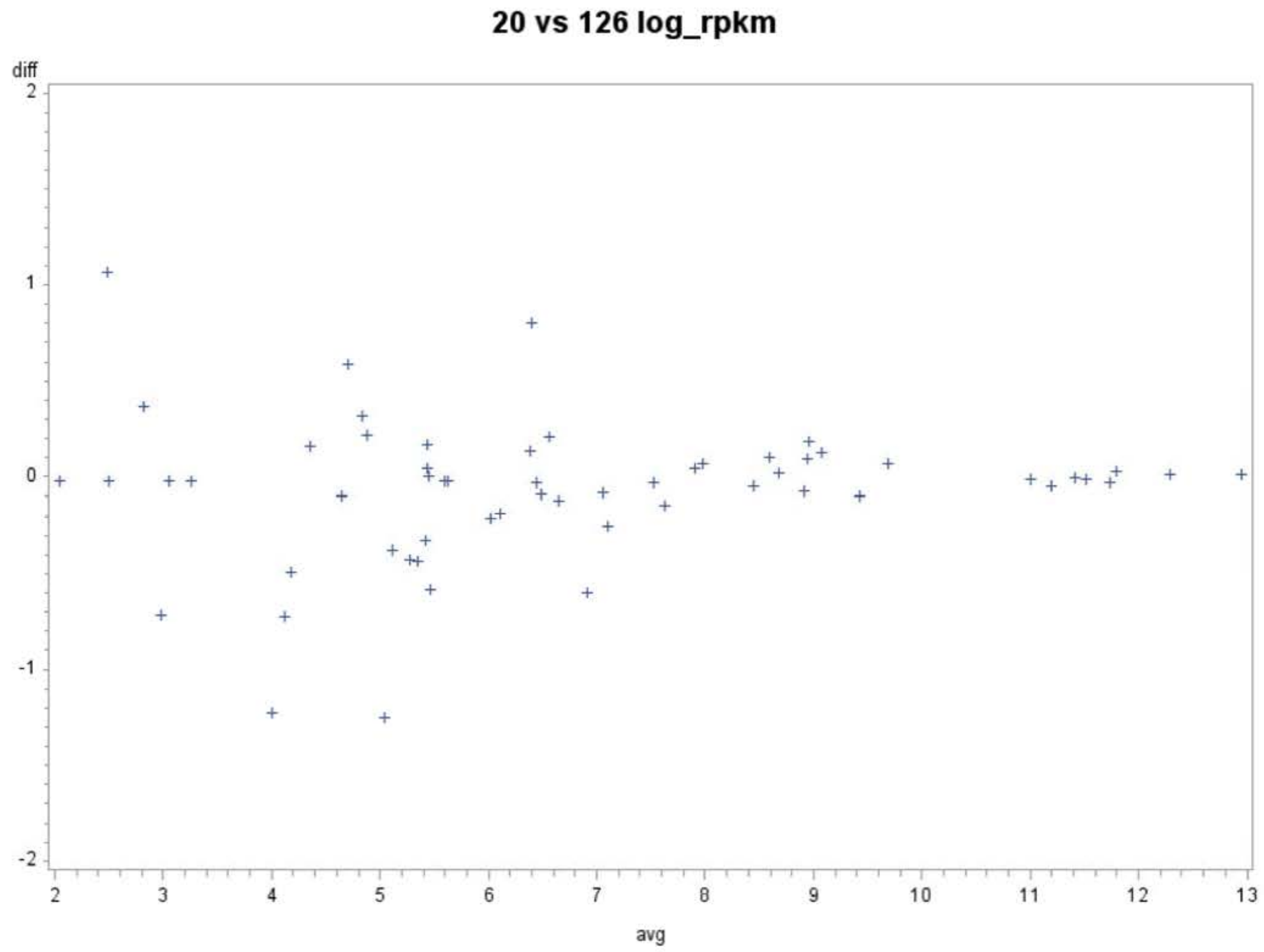

20 vs 126 log_rpkm
diff
2
1
0
-1
-2
2
3
4
5
6
7
8
9
10
11
12
13
avg

35 vs 87 log_rpkm

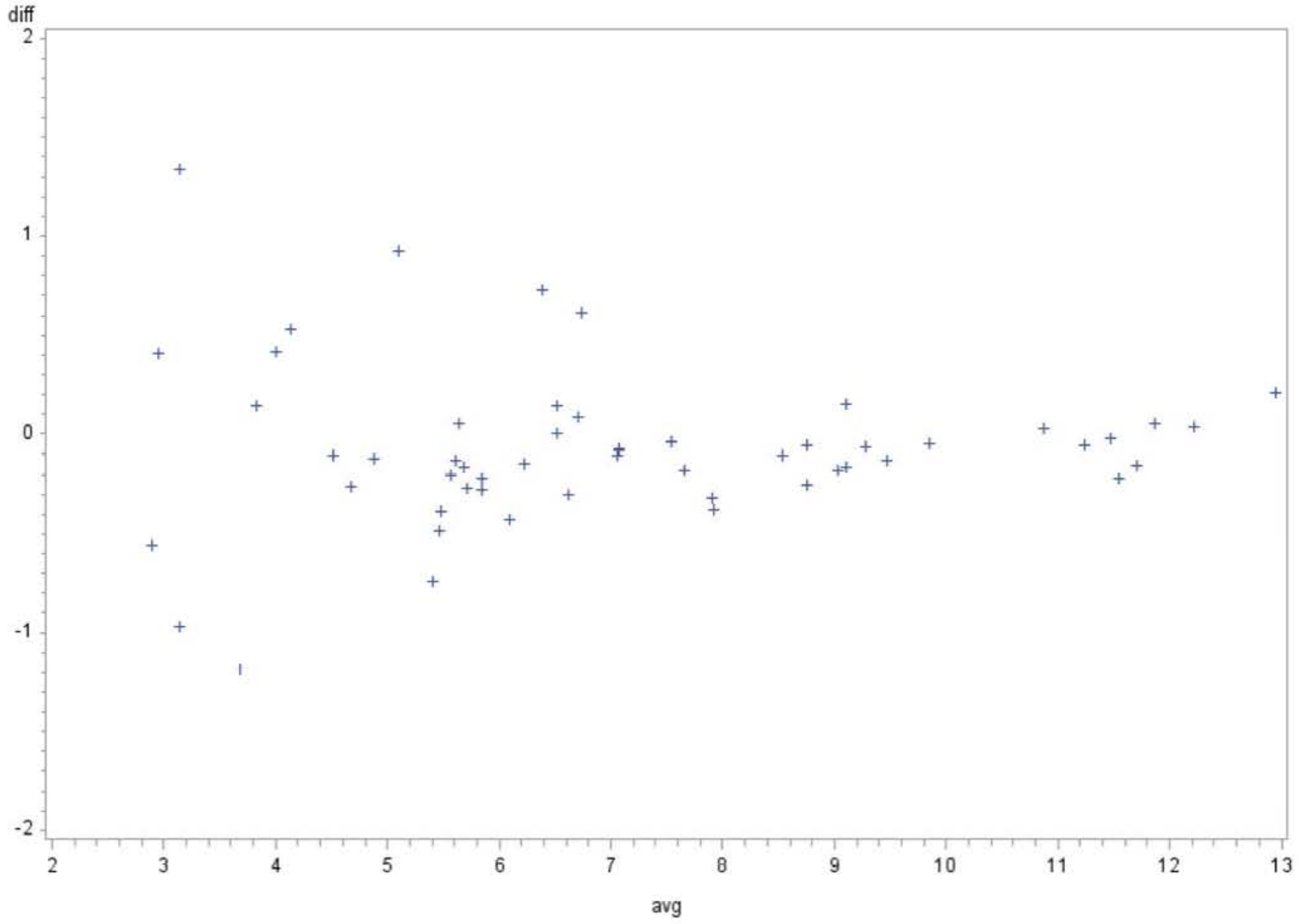

35 vs 103 log_rpkm

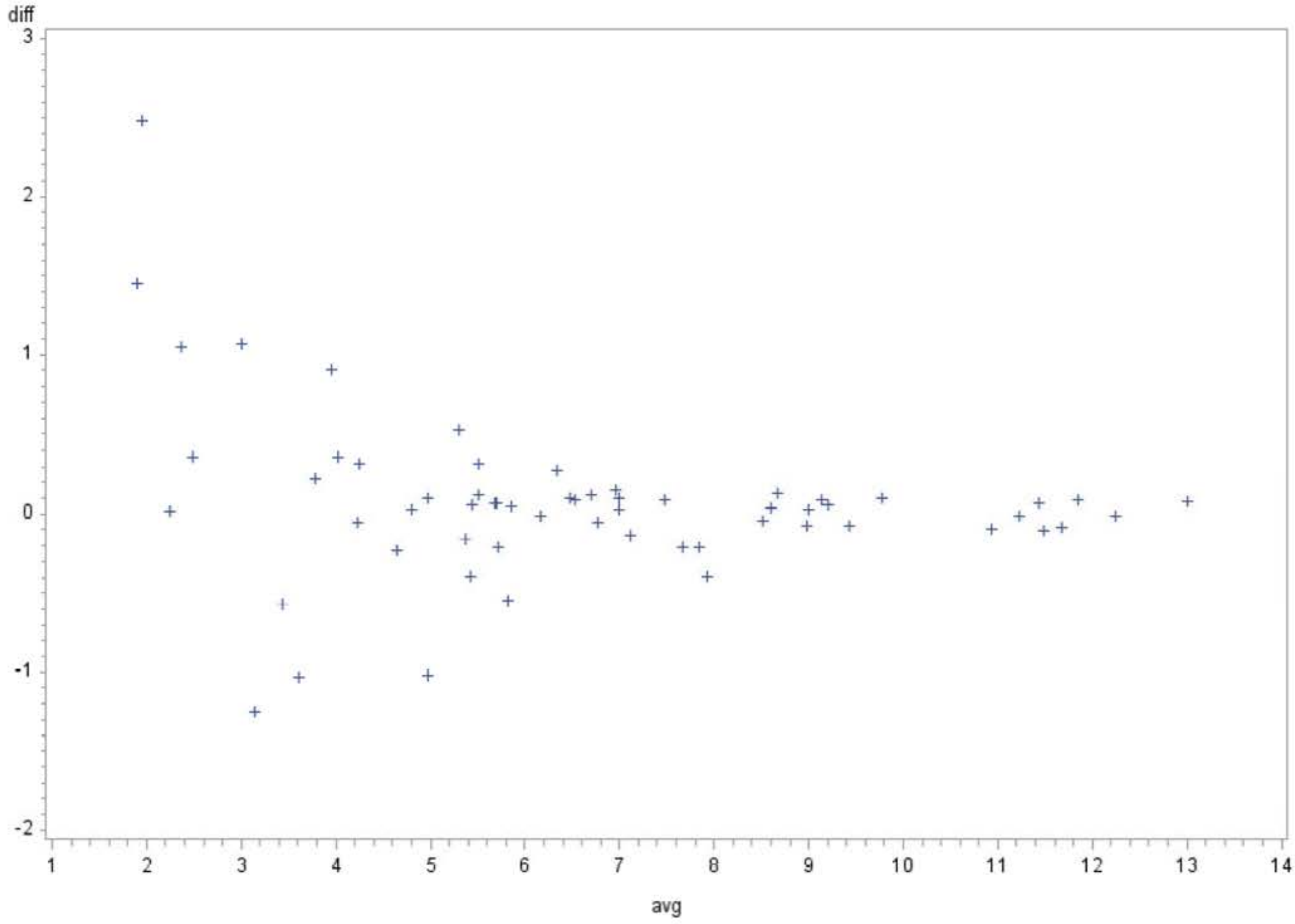

35 vs 126 log_rpkm

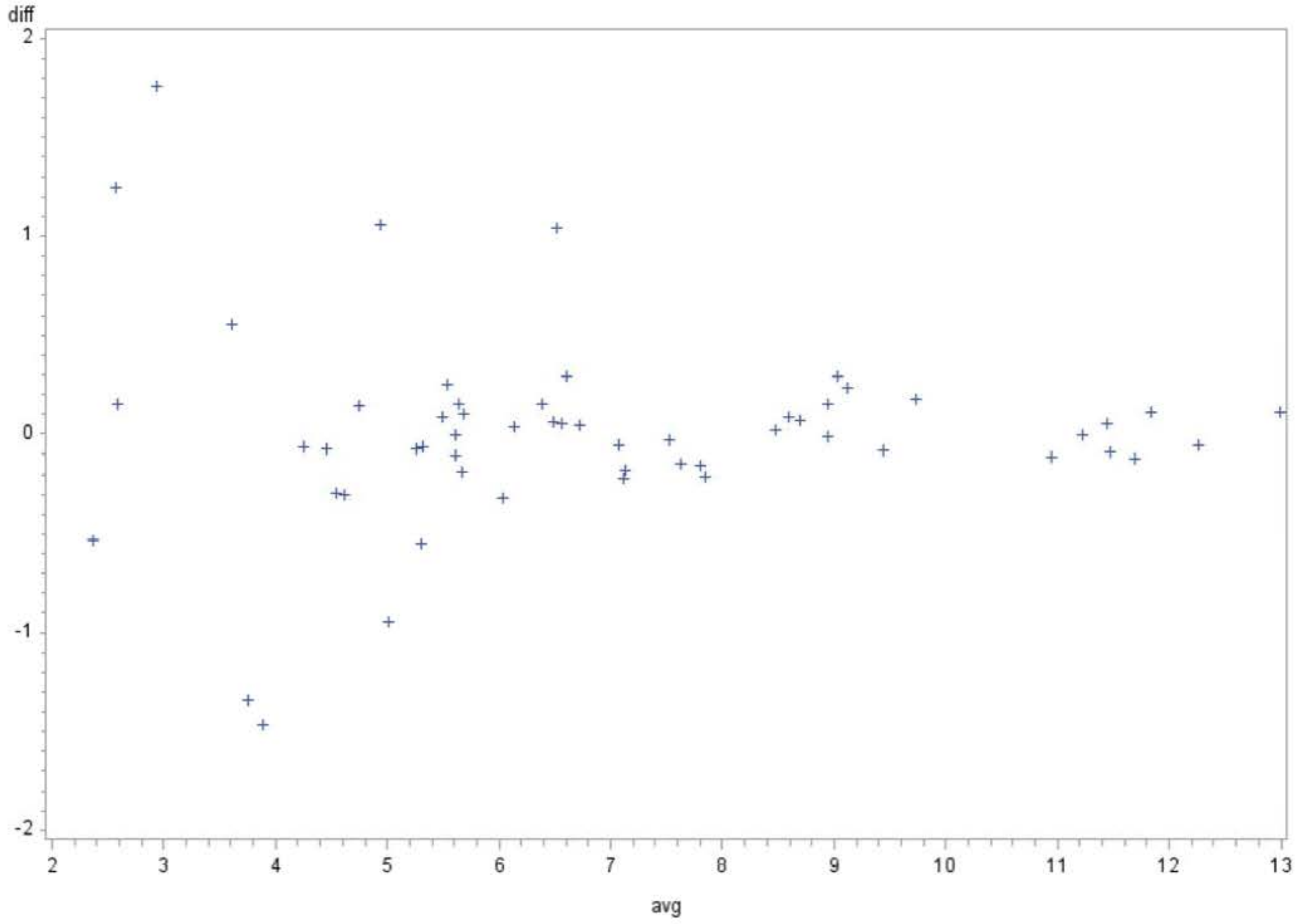

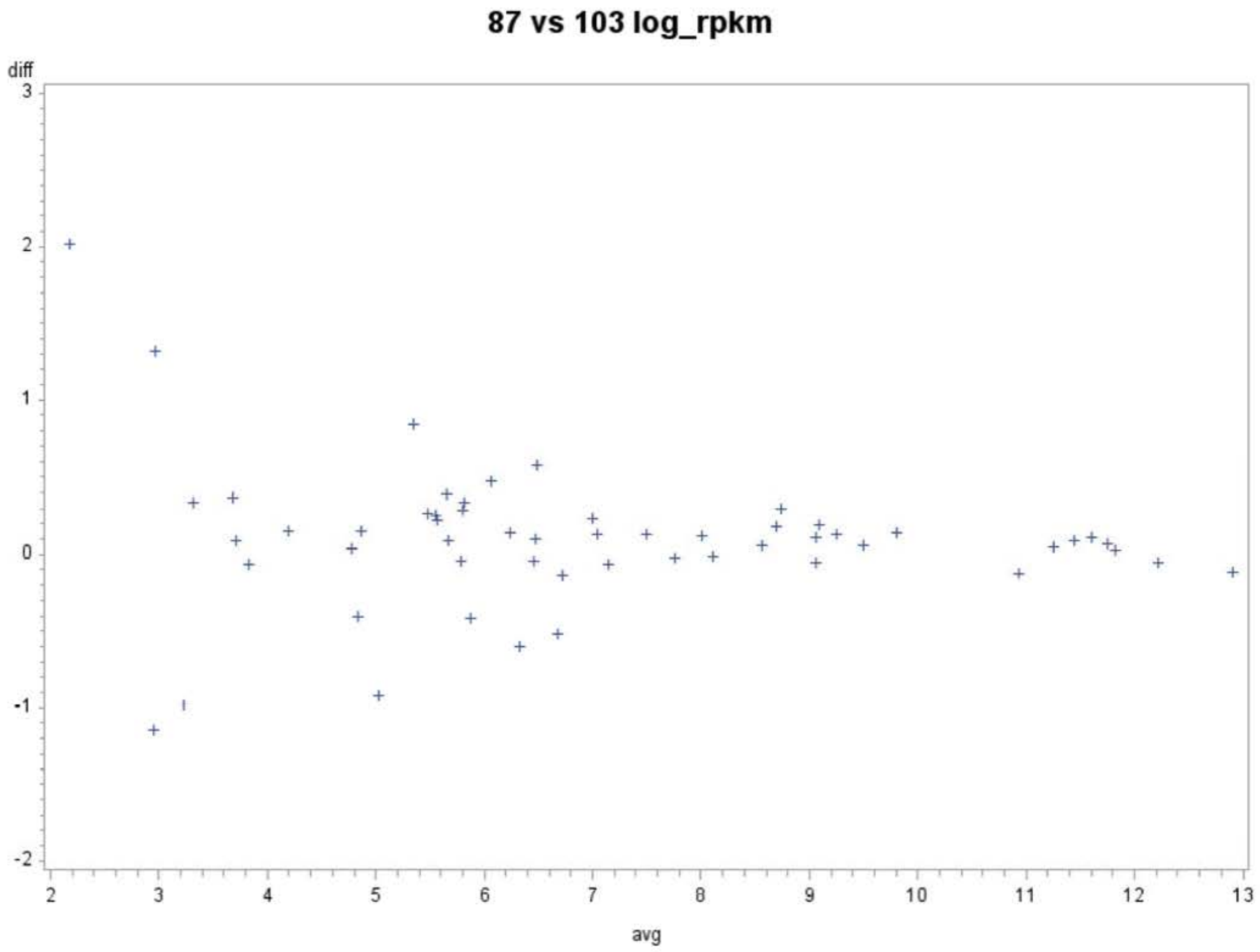
87 vs 103 log_rpkm
diff
3
2
1
0
-1
-2
2
3
4
5
6
7
8
9
10
11
12
13
avg

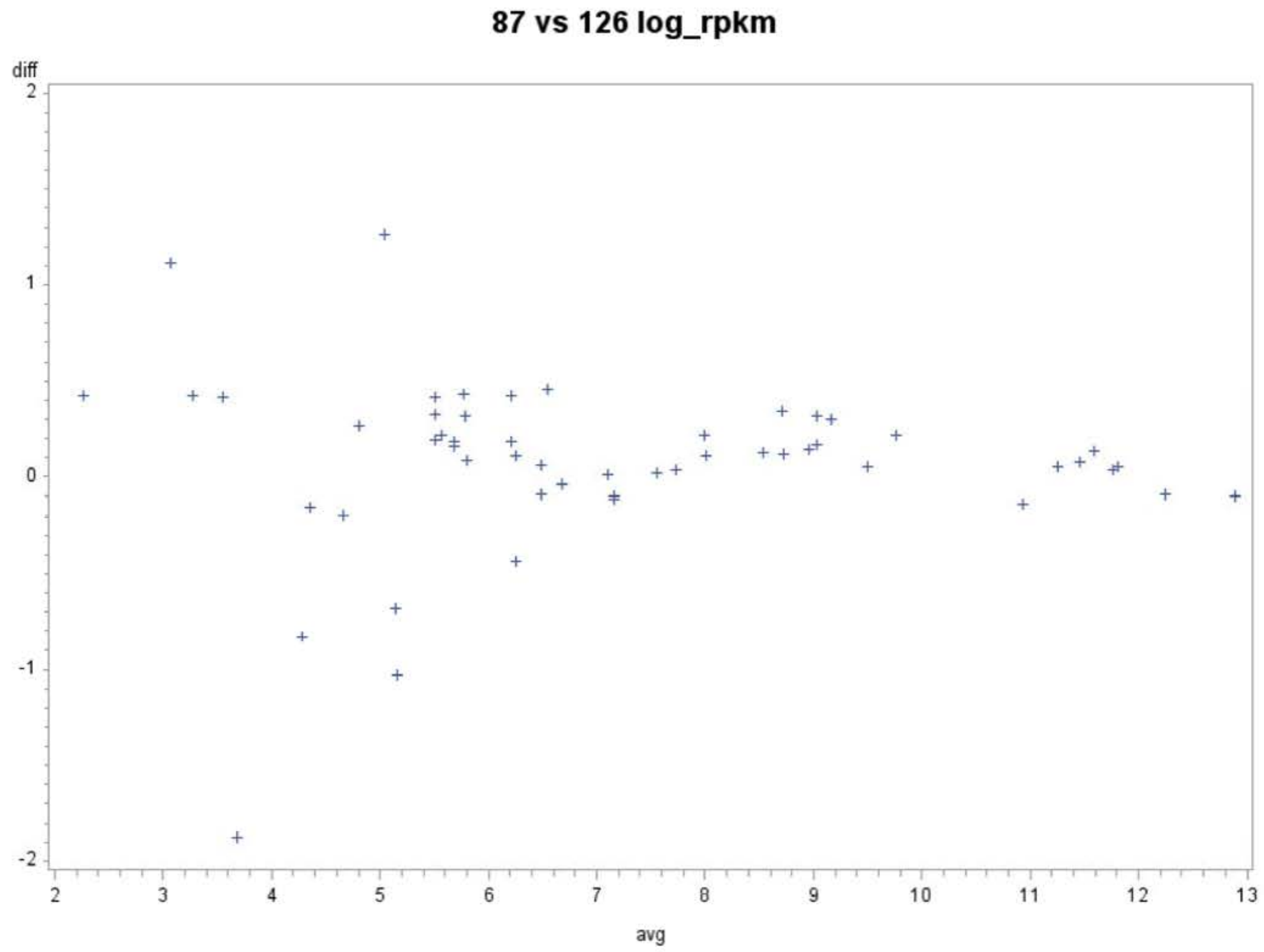
87 vs 126 log_rpkm
diff
2
1
0
-1
-2
2
3
4
5
6
7
8
9
10
11
12
13
avg

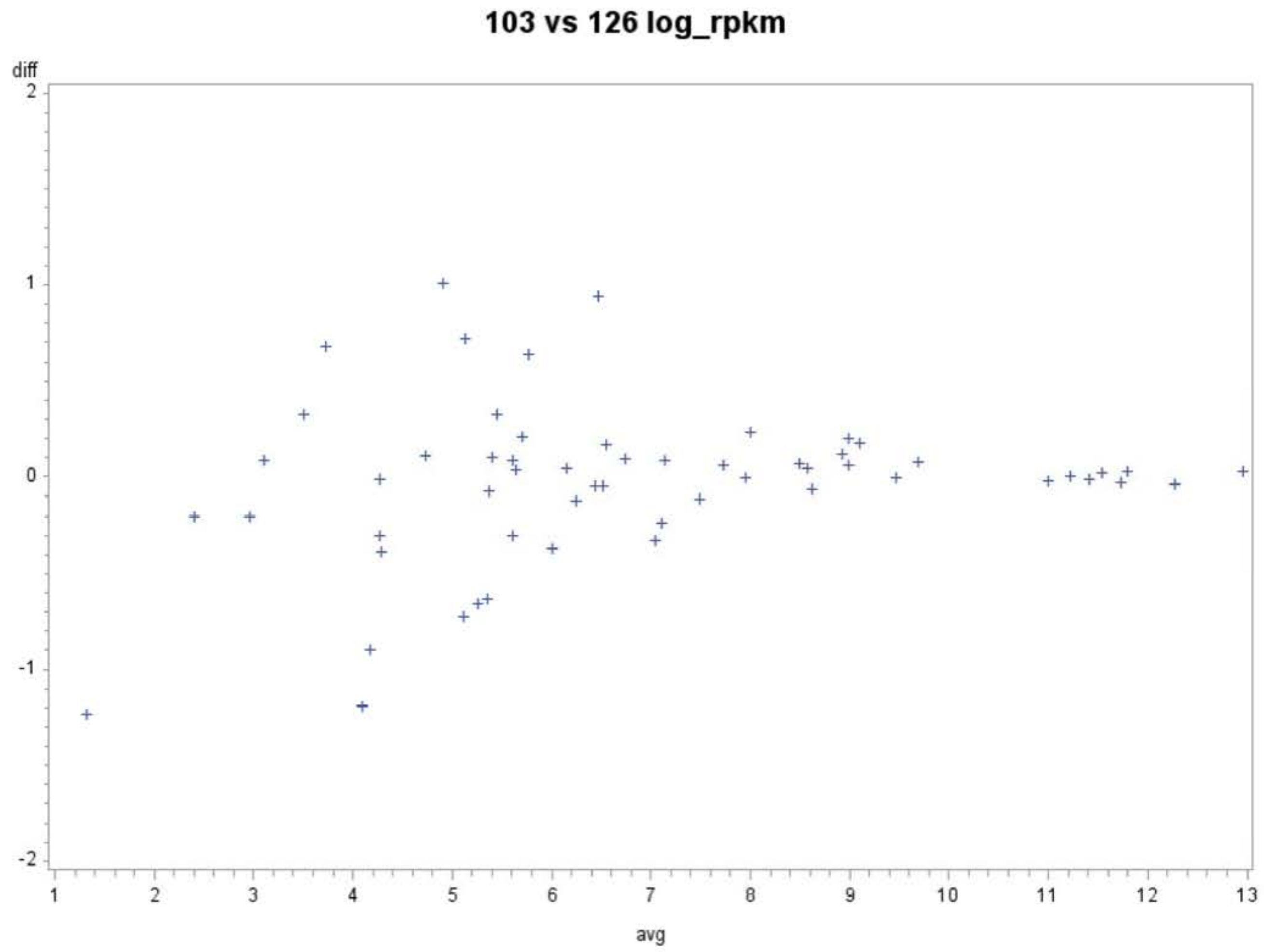
103 vs 126 log_rpkm
diff
2
1
0
-1
-2
1
2
3
4
5
6
7
8
9
10
11
12
13
avg

81 vs 4 log_rpkm

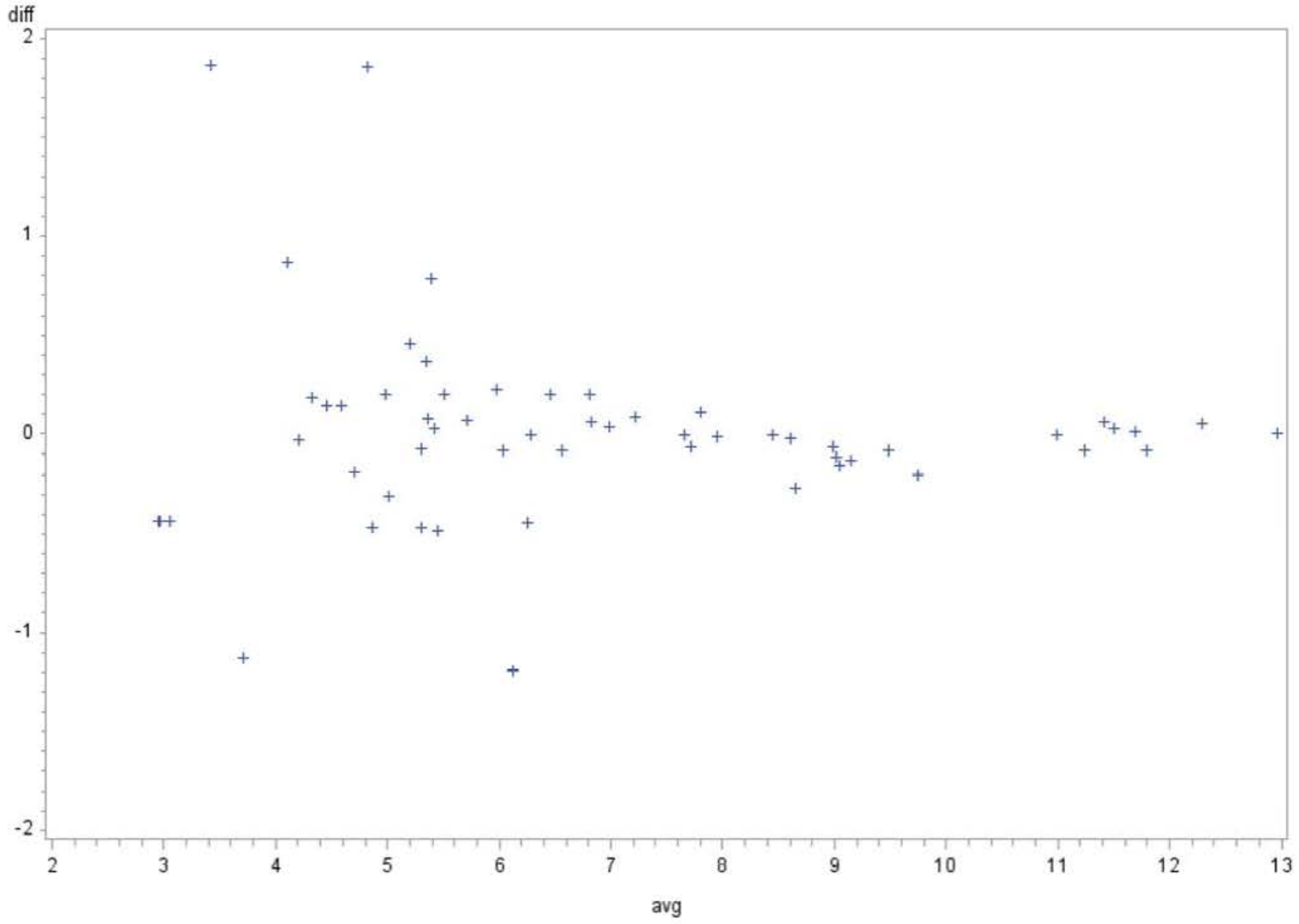

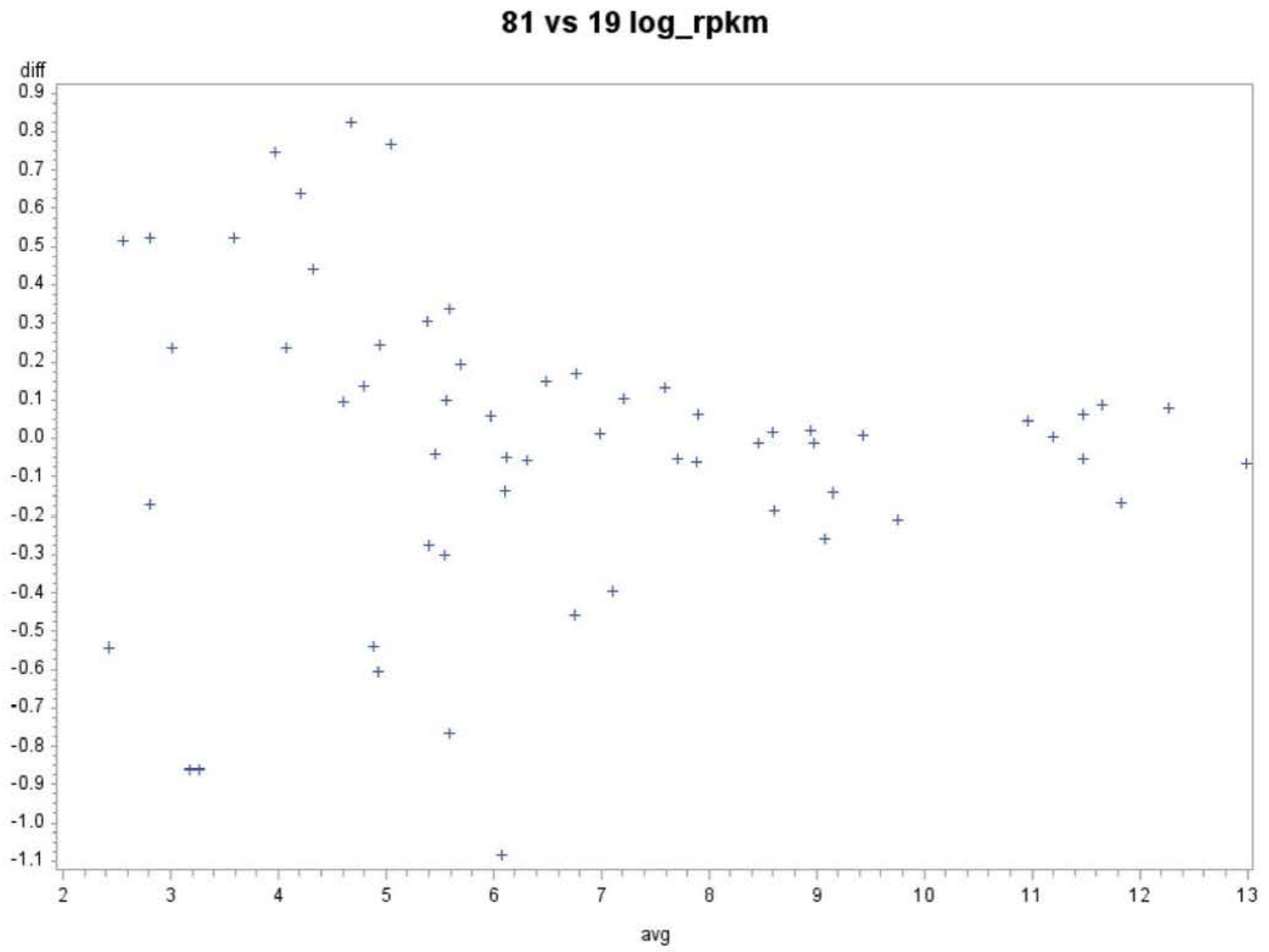
81 vs 19 log_rpkm
diff
0.9
0.8
0.7
0.6
0.5
0.4
0.3
0.2
0.1
0.0
-0.1
-0.2
-0.3
-0.4
-0.5
-0.6
-0.7
-0.8
-0.9
-1.0
-1.1
2
3
4
5
6
7
8
9
10
11
12
13
avg

81 vs 34 log_rpkm

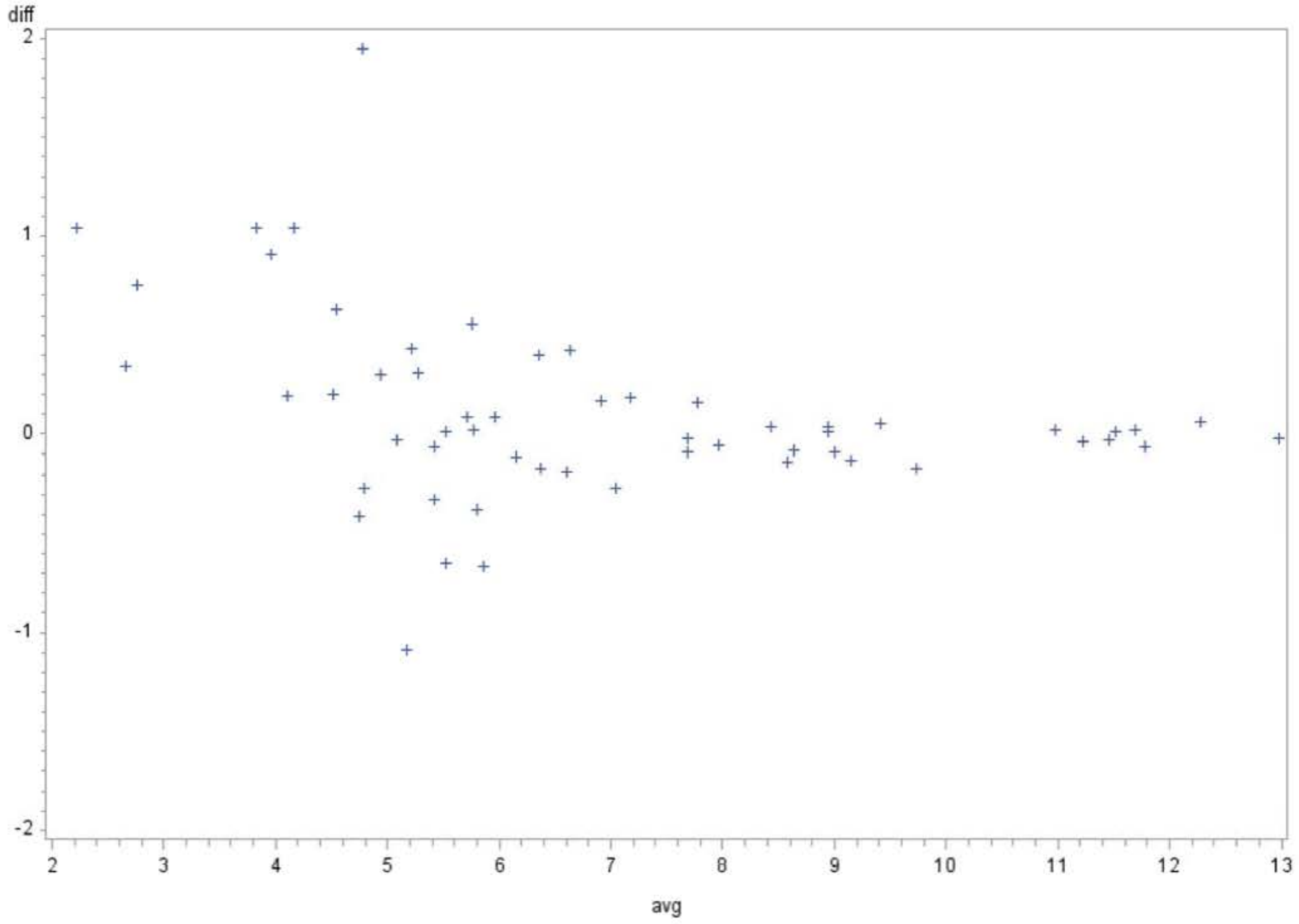

f

81 vs 70 log_rpkm

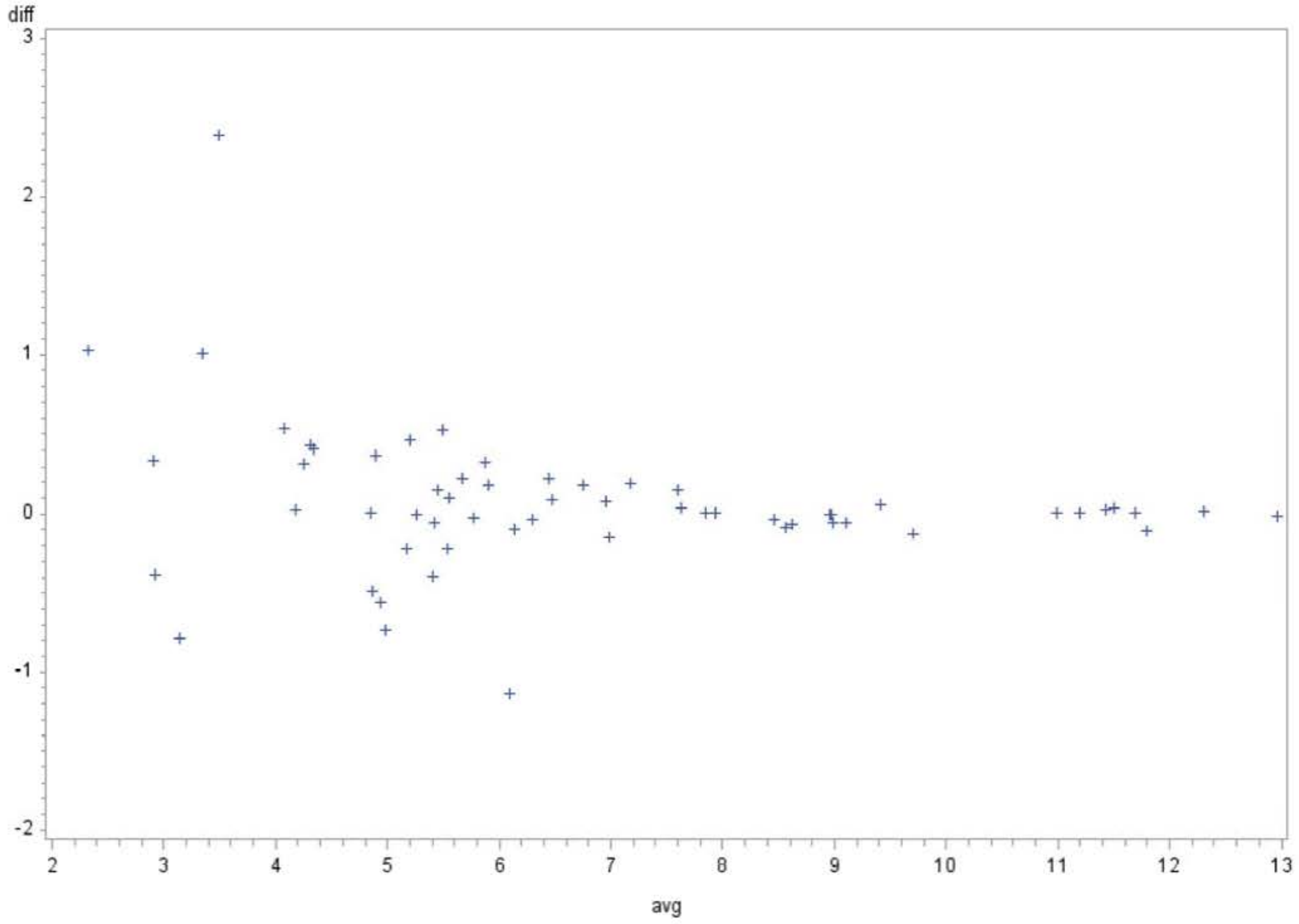

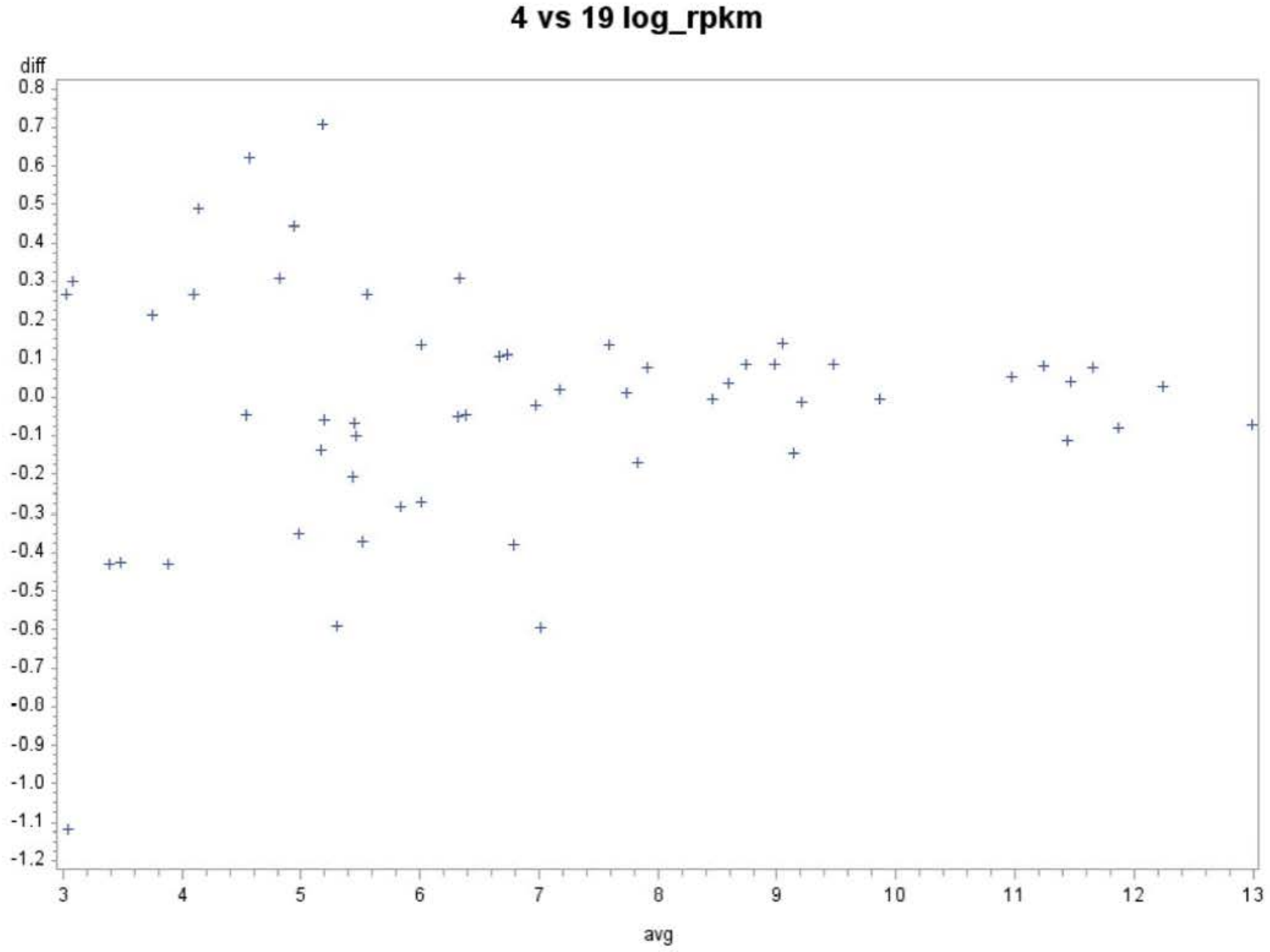
4 vs 19 log_rpkm
diff
0.8
0.7
0.6
0.5
0.4
0.3
0.2
0.1
0.0
-0.1
-0.2
-0.3
-0.4
-0.5
-0.6
-0.7
-0.8
-0.9
-1.0
-1.1
-1.2
3
4
5
6
7
8
9
10
11
12
13
avg

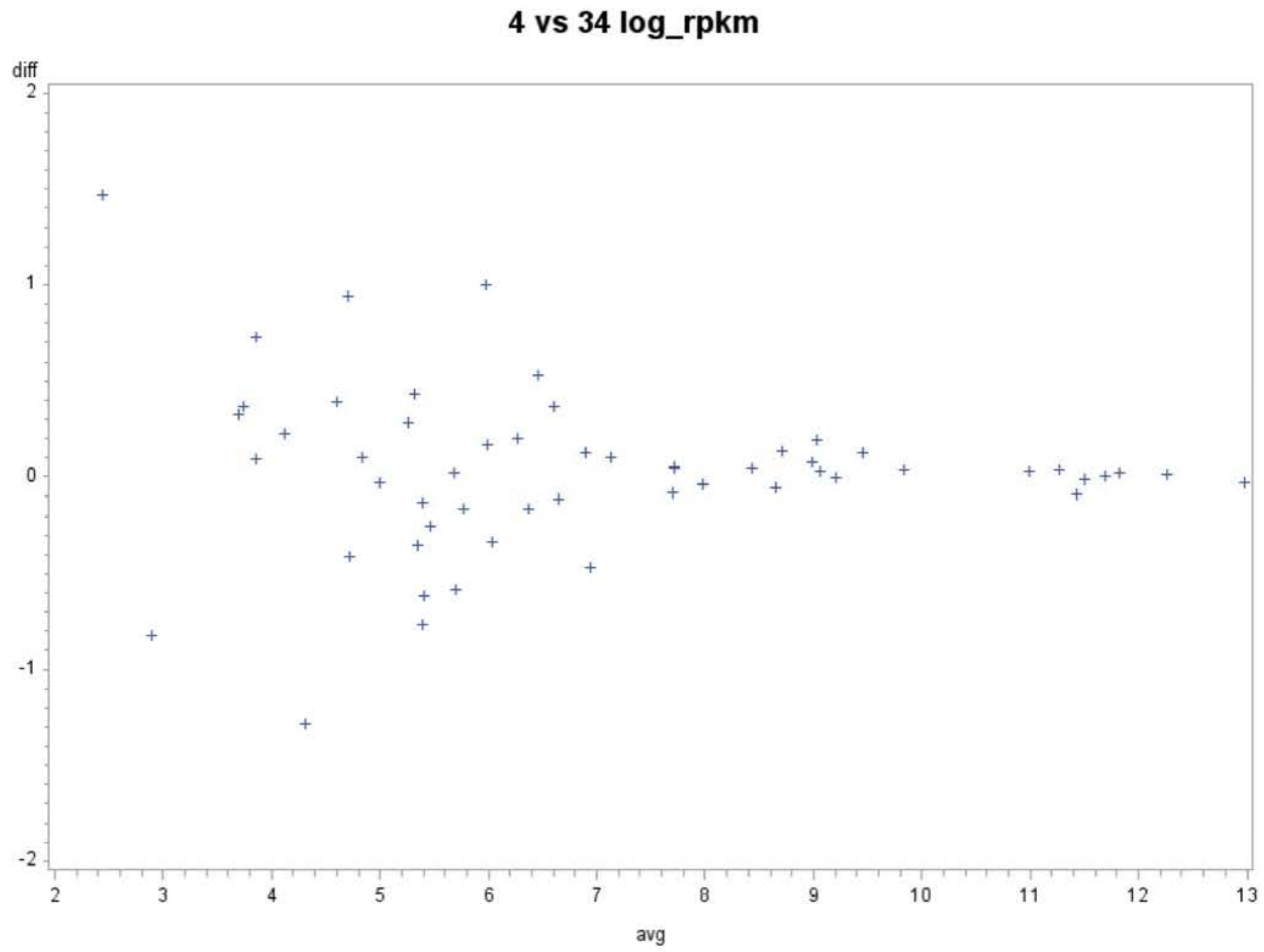

4 vs 34 log_rpkm
diff
2
1
0
-1
-2
2
3
4
5
6
7
8
9
10
11
12
13
avg

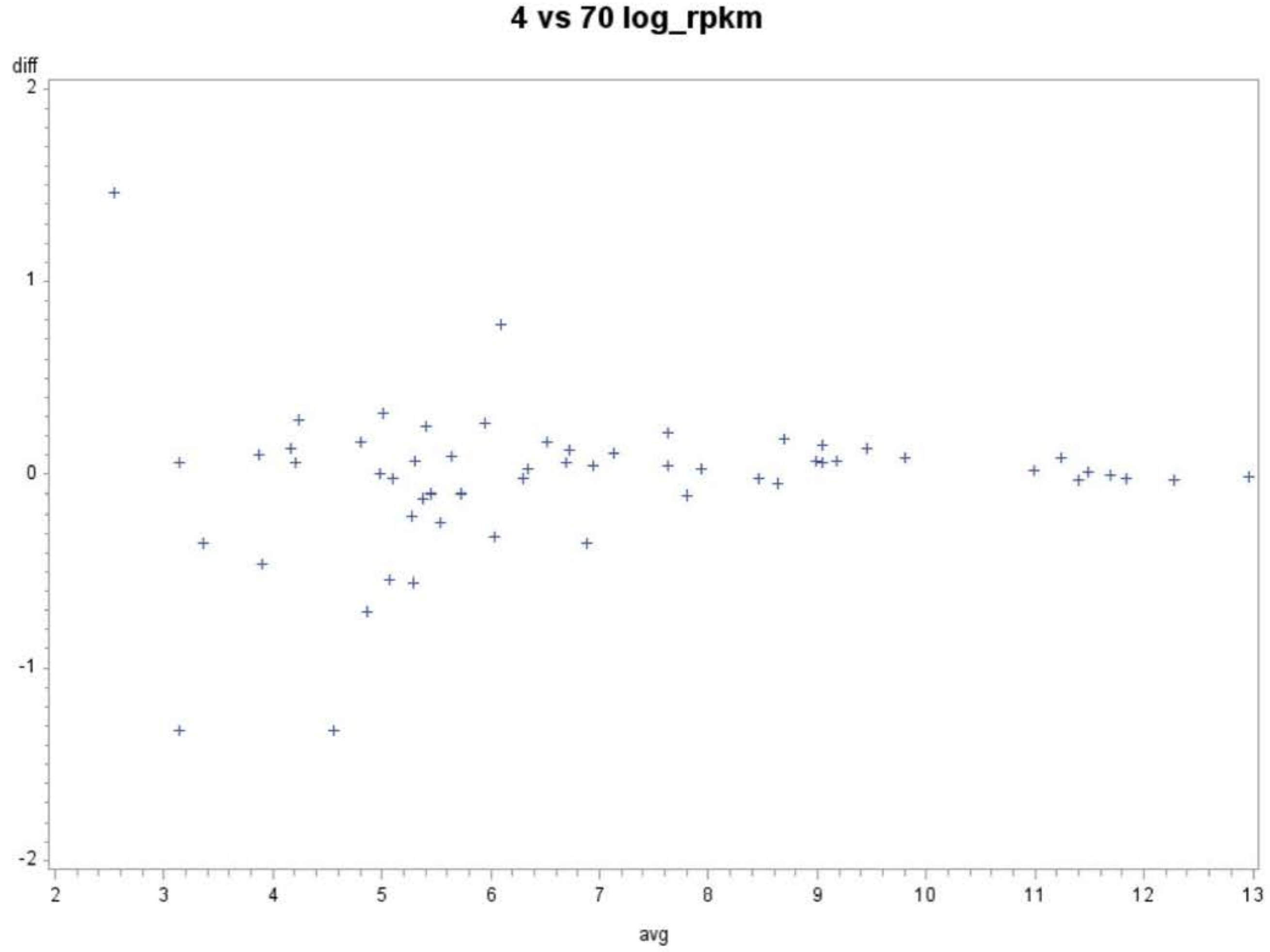
4 vs 70 log_rpkm
diff
2
1
0
-1
-2
2
3
4
5
6
7
8
9
10
11
12
13
avg

19 vs 34 log_rpkm

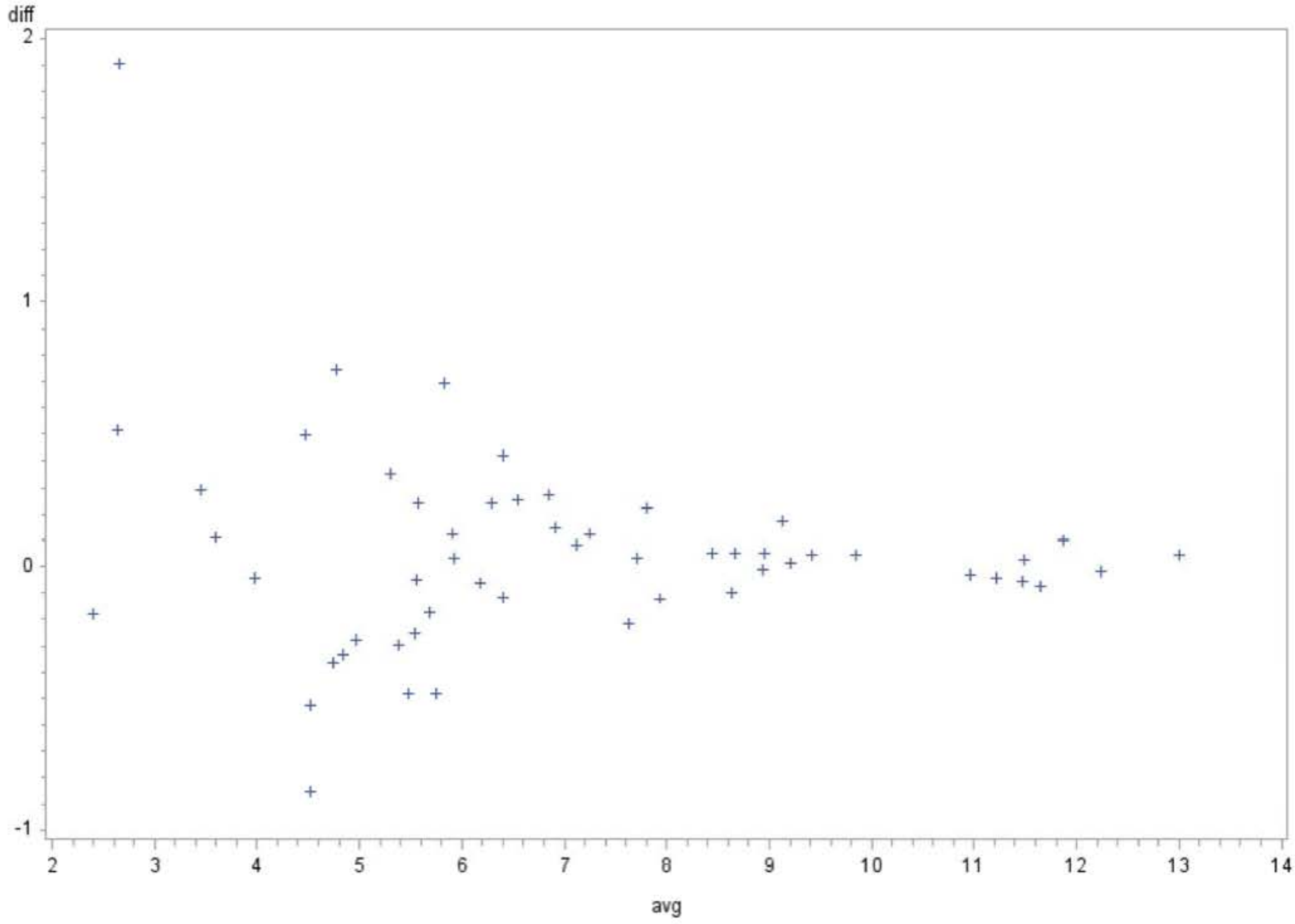

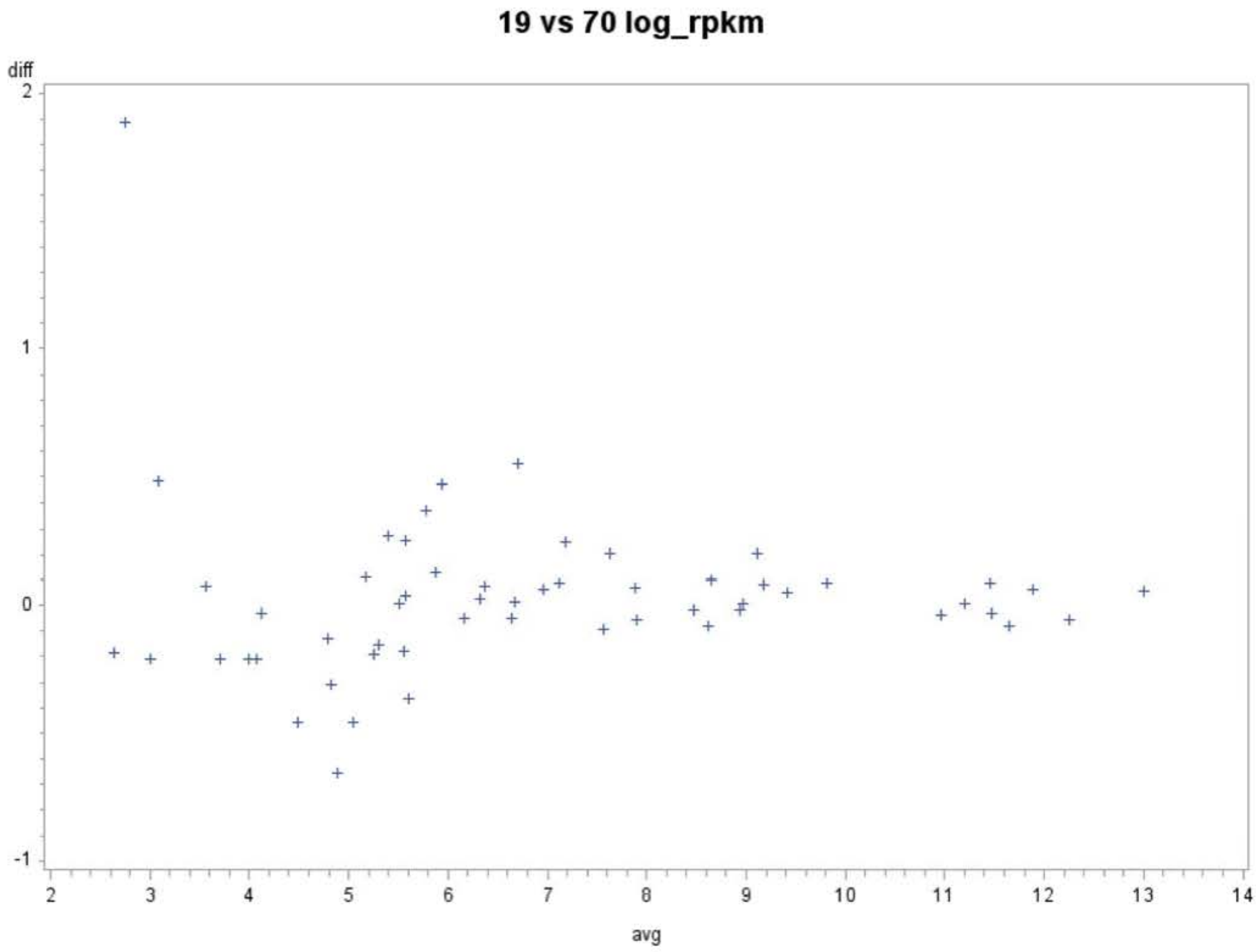
19 vs 70 log_rpkm
diff
2
1
0
-1
2
3
4
5
6
7
8
9
10
11
12
13
14
avg

34 vs 70 log_rpkm

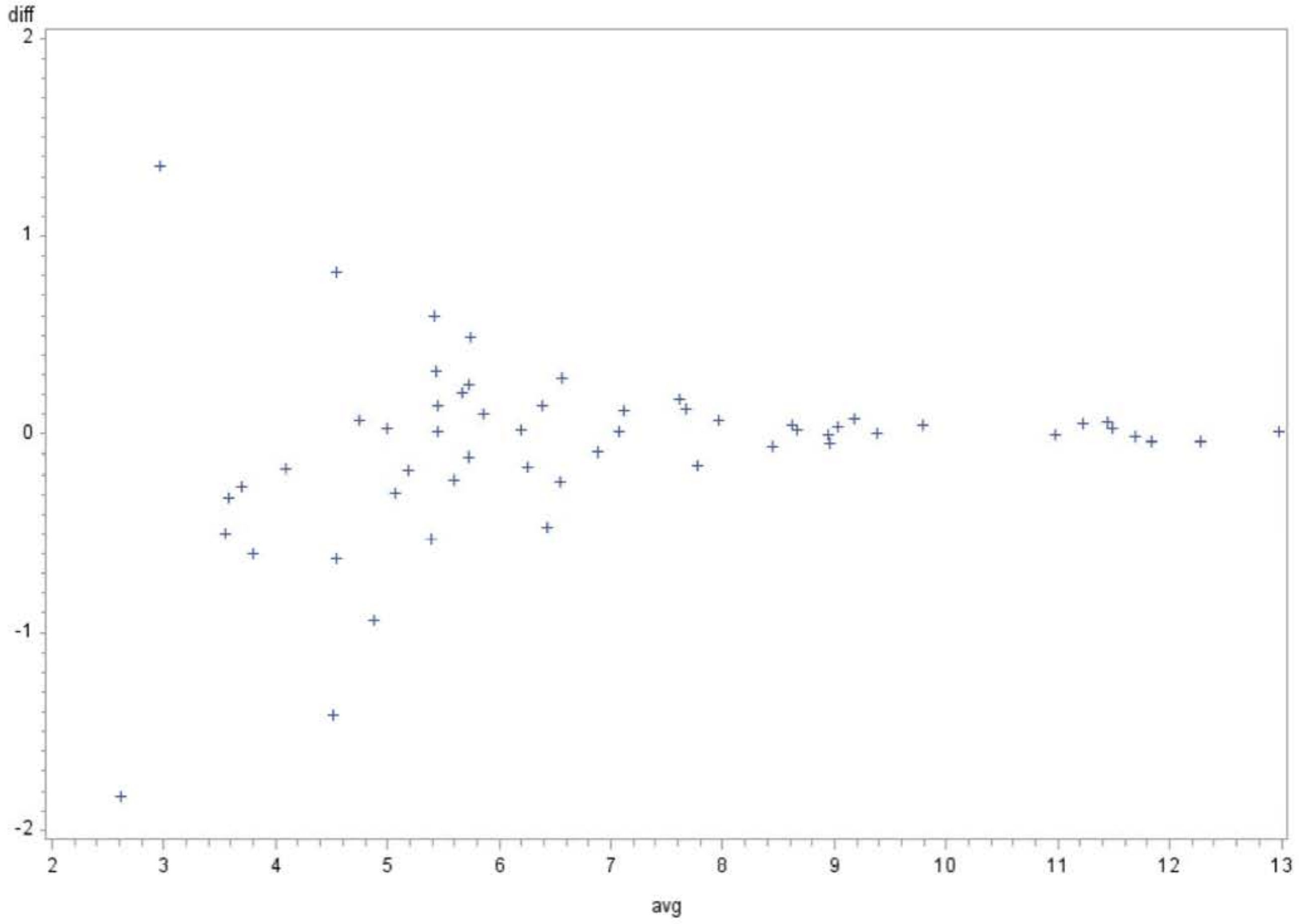

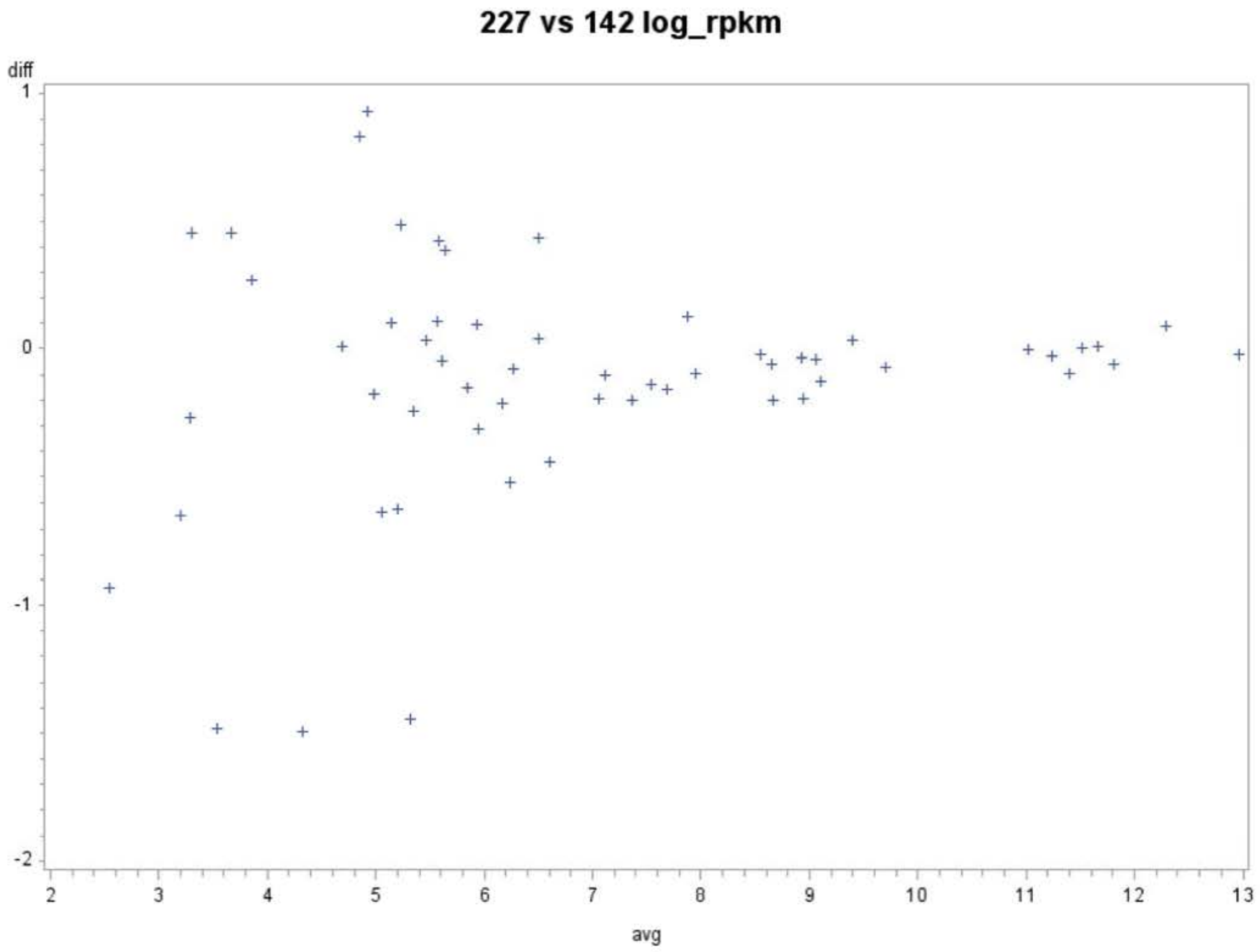

227 vs 146 log_rpkm

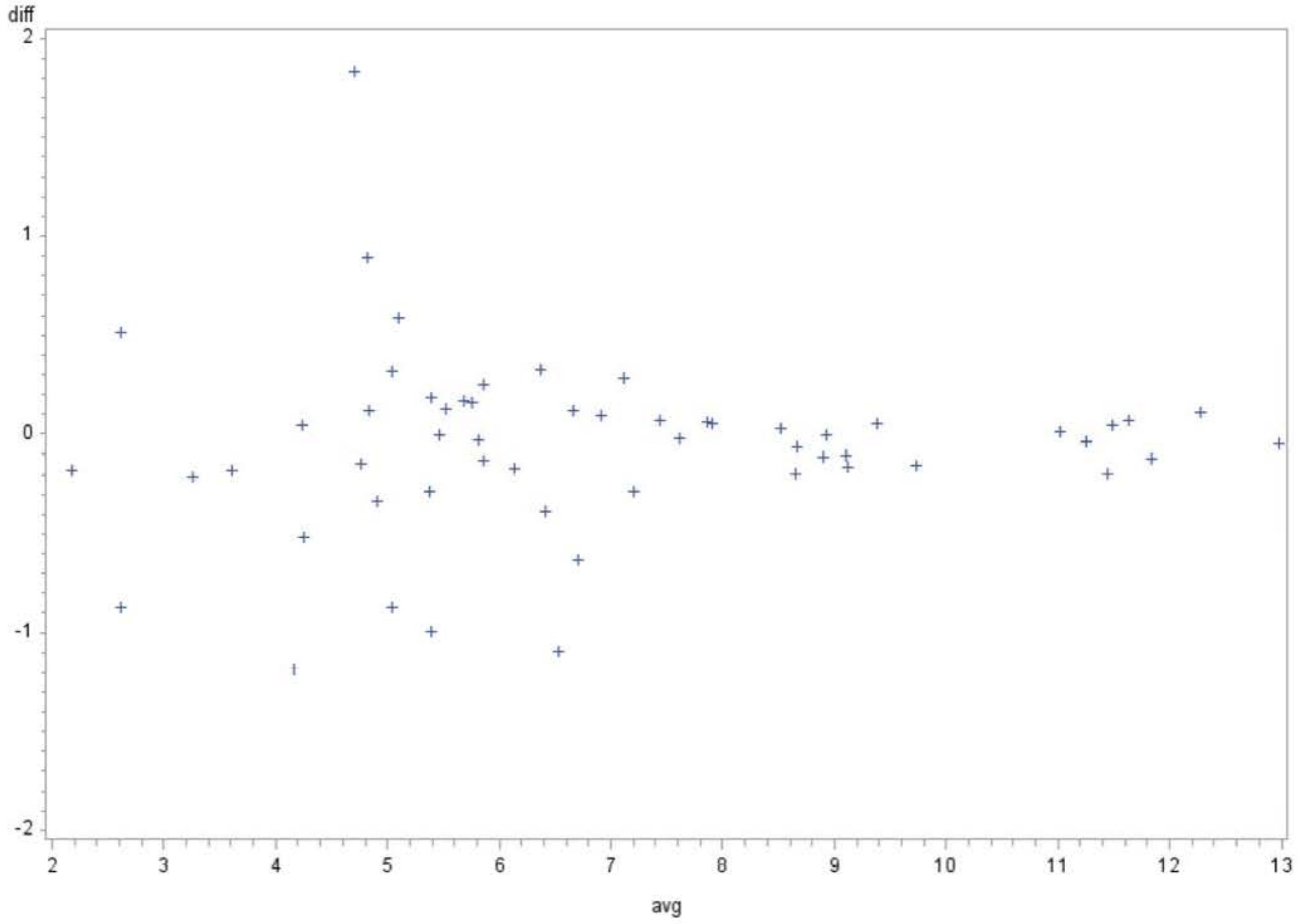

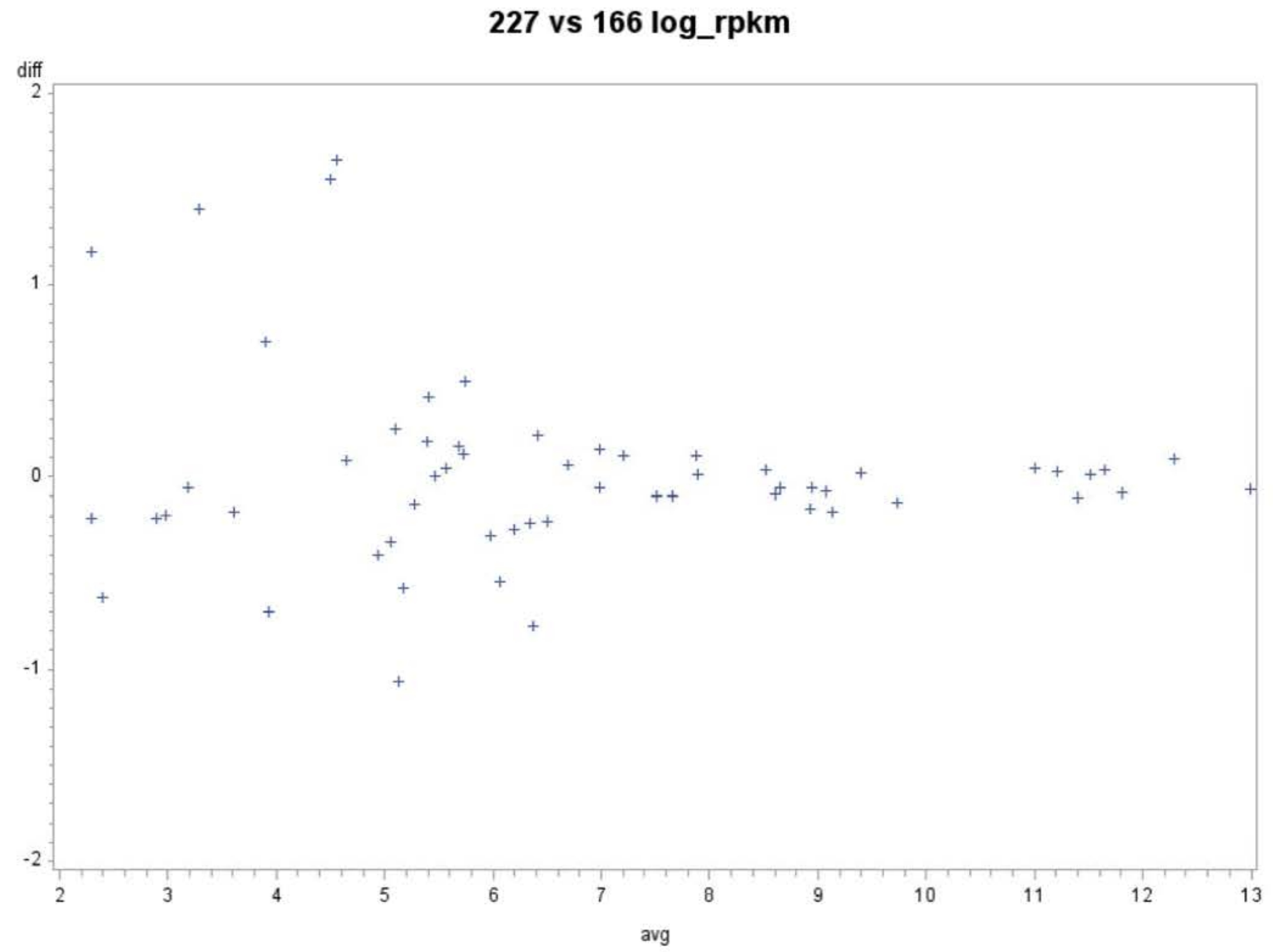
227 vs 166 log_rpkm
diff
2
1
0
-1
-2
2
3
4
5
6
7
8
9
10
11
12
13
avg

227 vs 181 log_rpkm

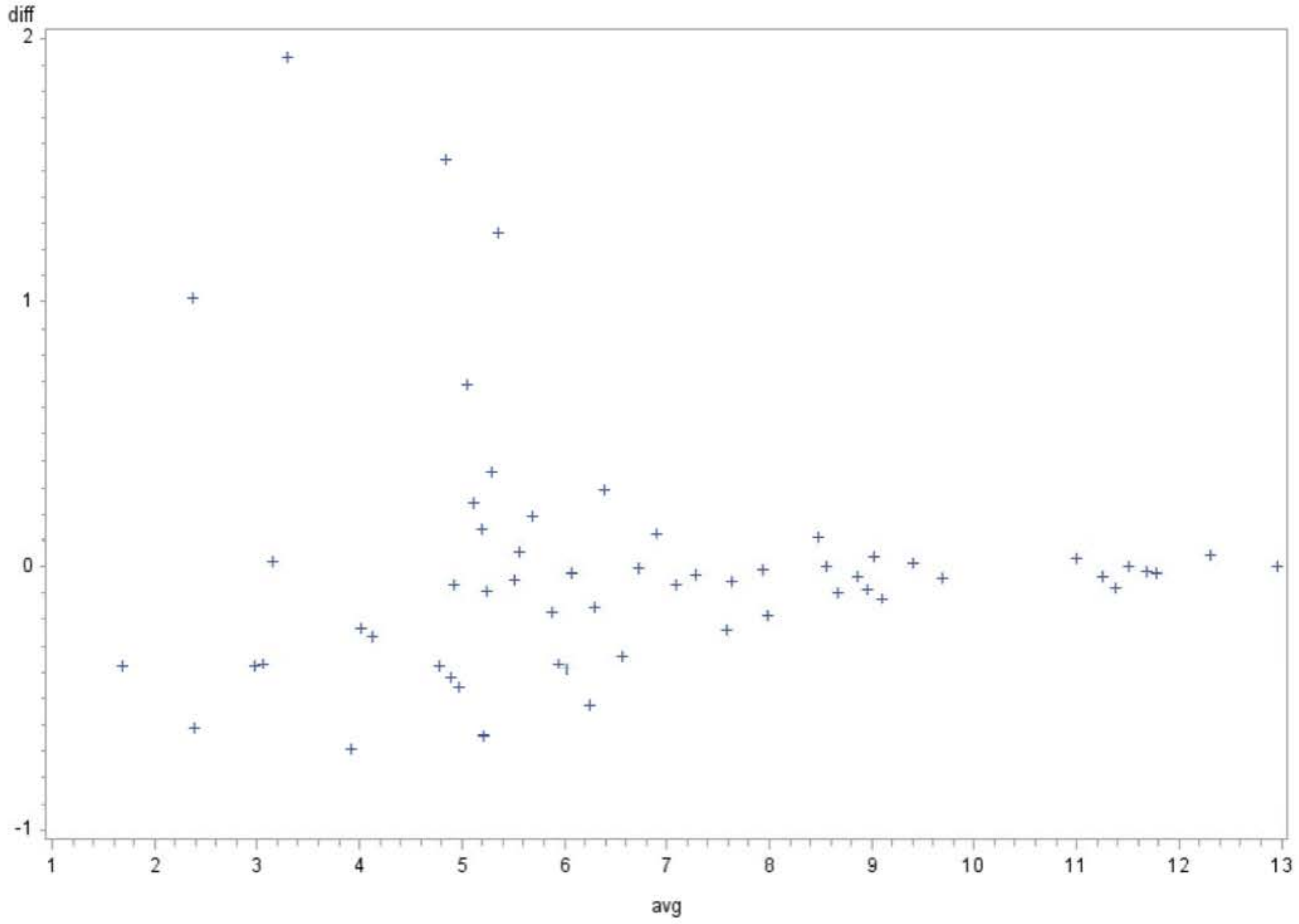

227 vs 217 log_rpkm

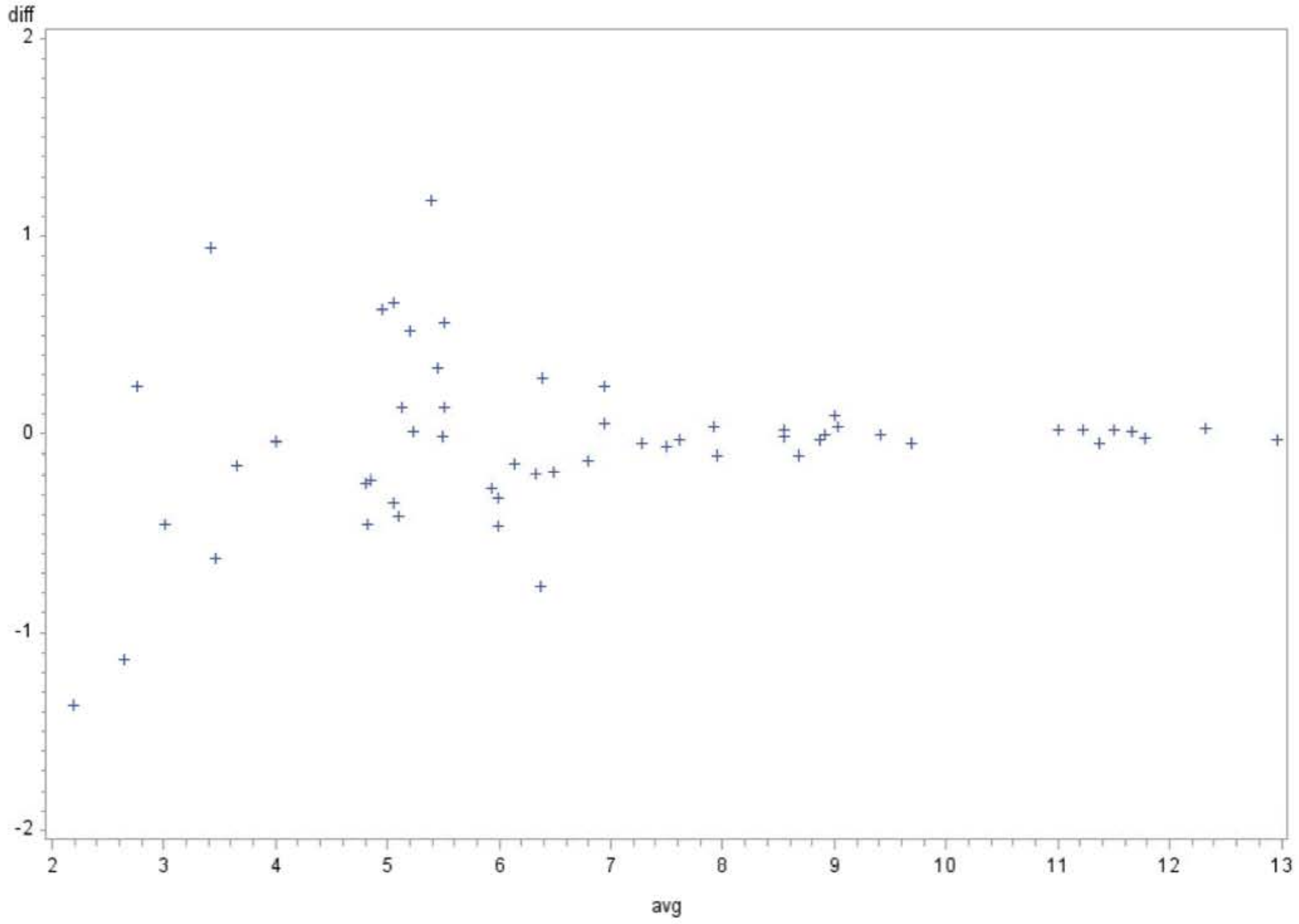

142 vs 146 log_rpkm

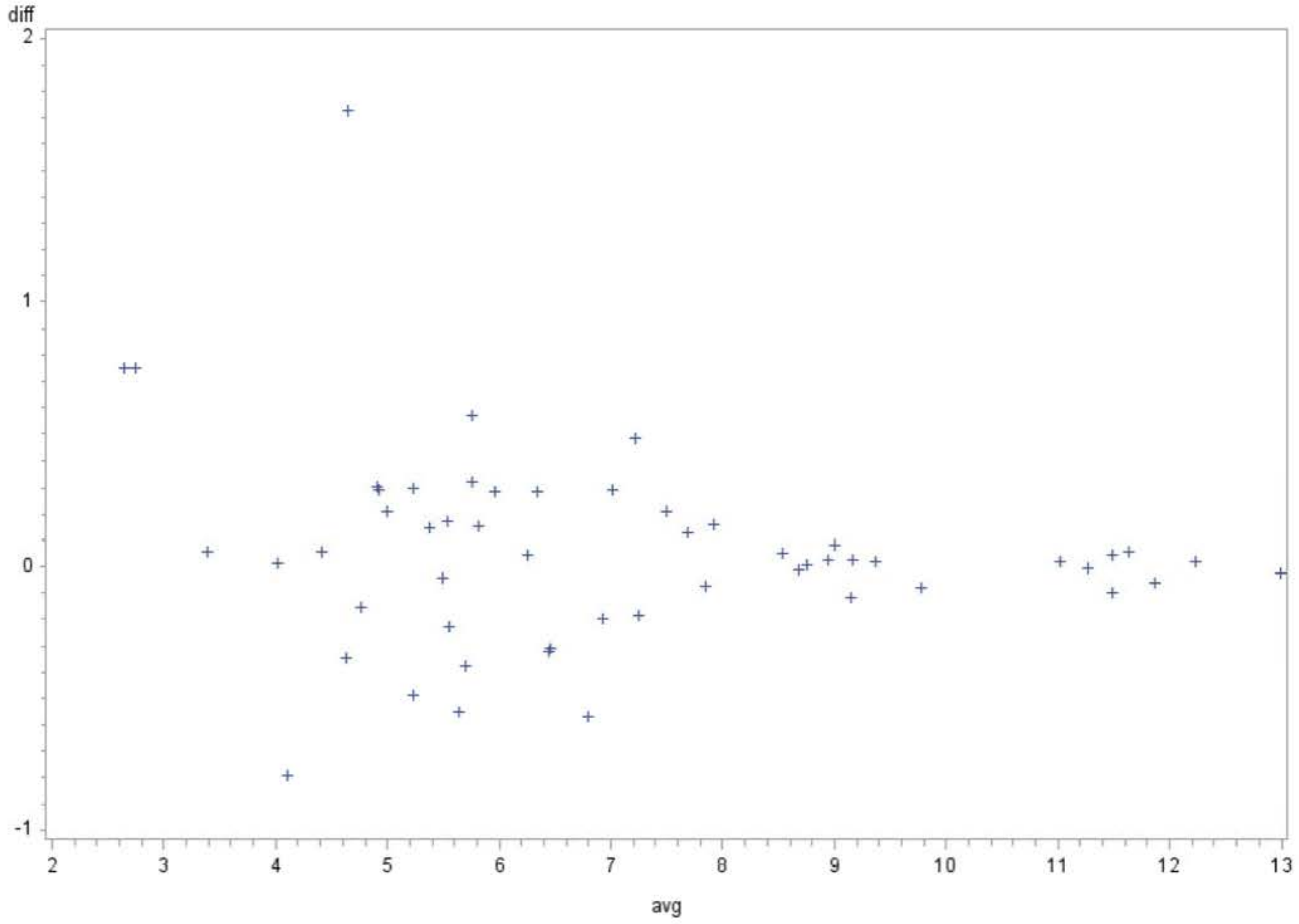

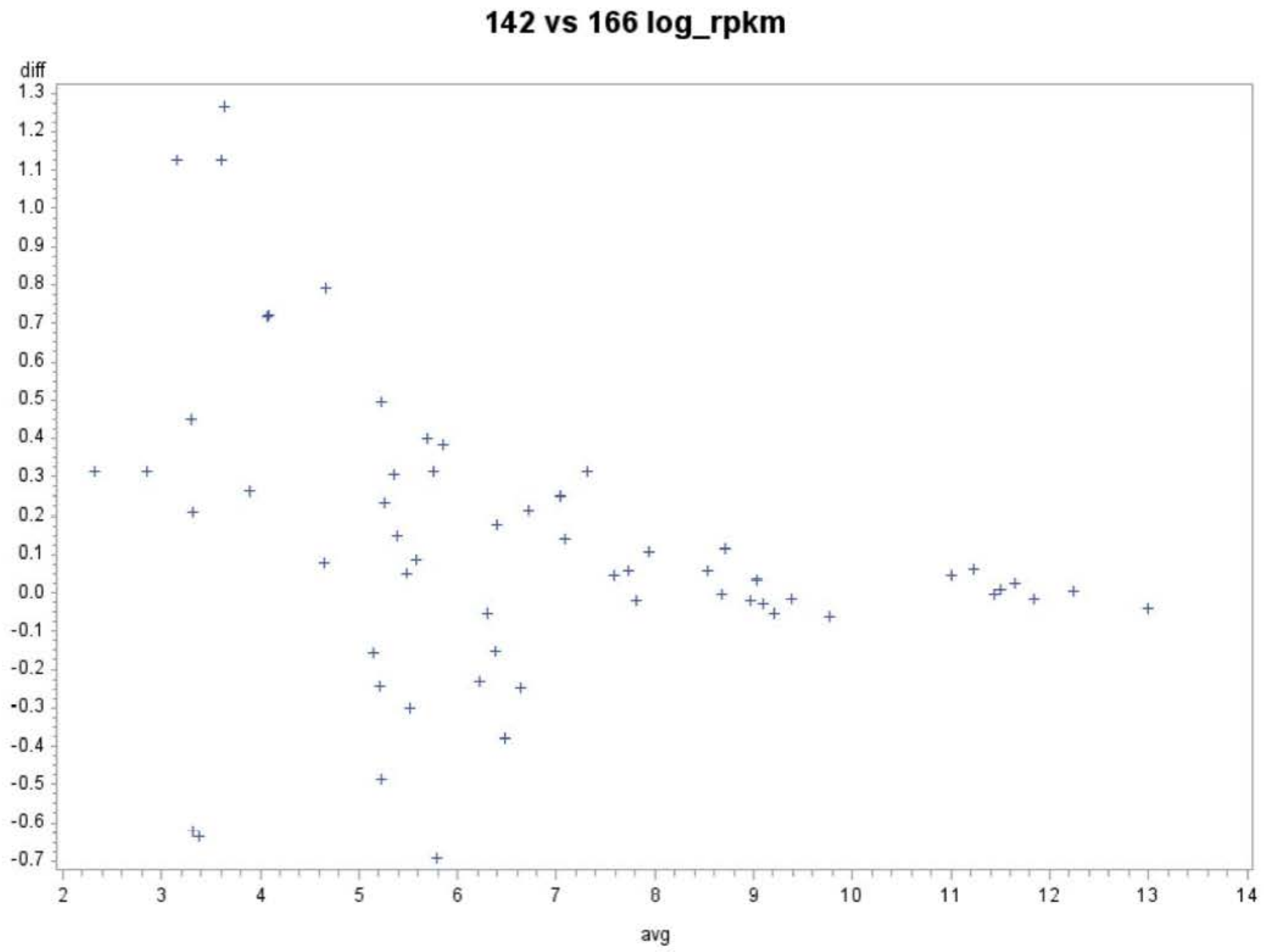

142 vs 166 log_rpkm
diff
1.3
1.2
1.1
1.0
0.9
0.8
0.7
0.6
0.5
0.4
0.3
0.2
0.1
0.0
-0.1
-0.2
-0.3
-0.4
-0.5
-0.6
-0.7
2
3
4
5
6
7
8
9
10
11
12
13
14
avg

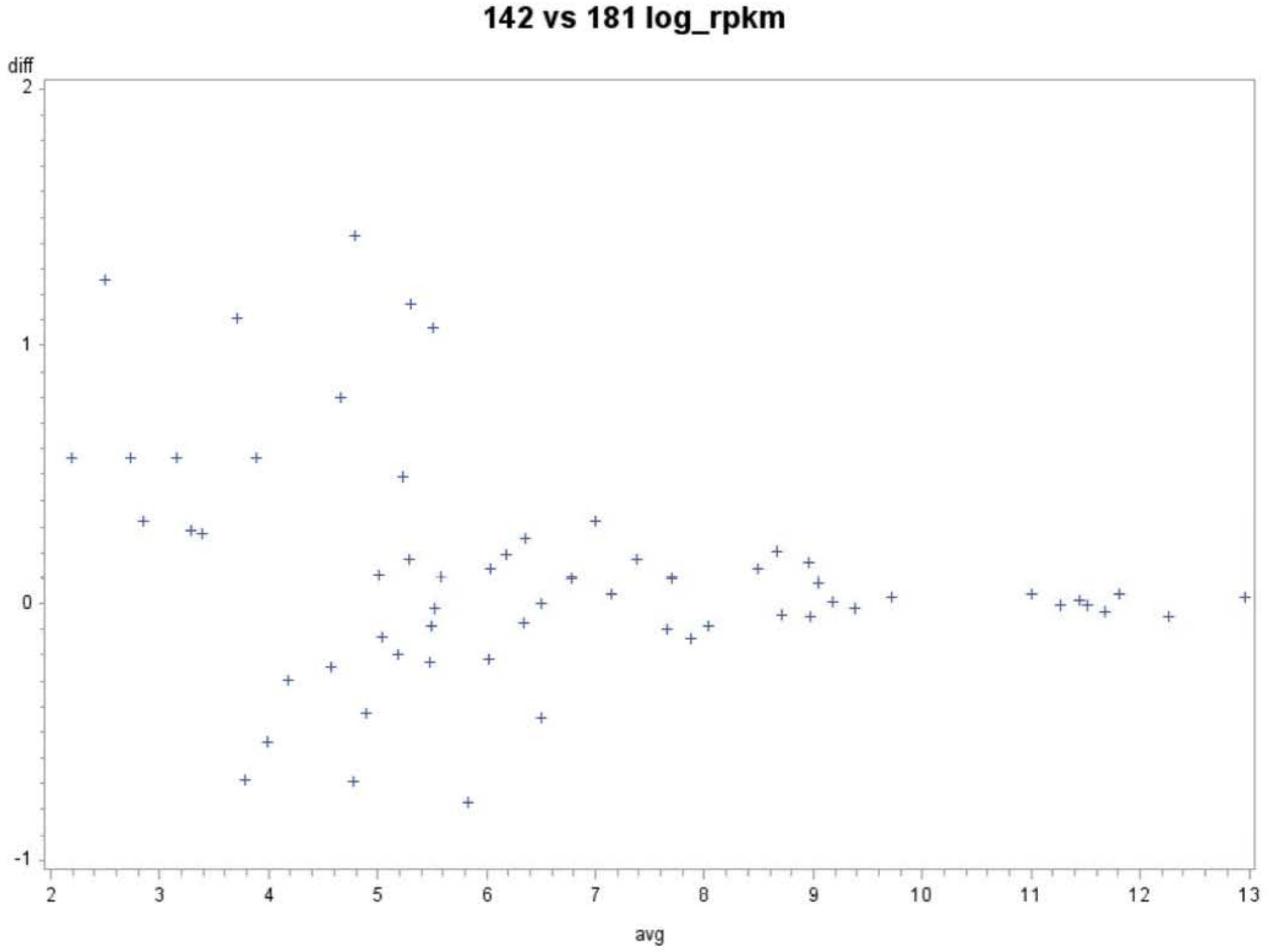

142 vs 181 log_rpkm
diff
2
1
0
-1
2
3
4
5
6
7
8
9
10
11
12
13
avg

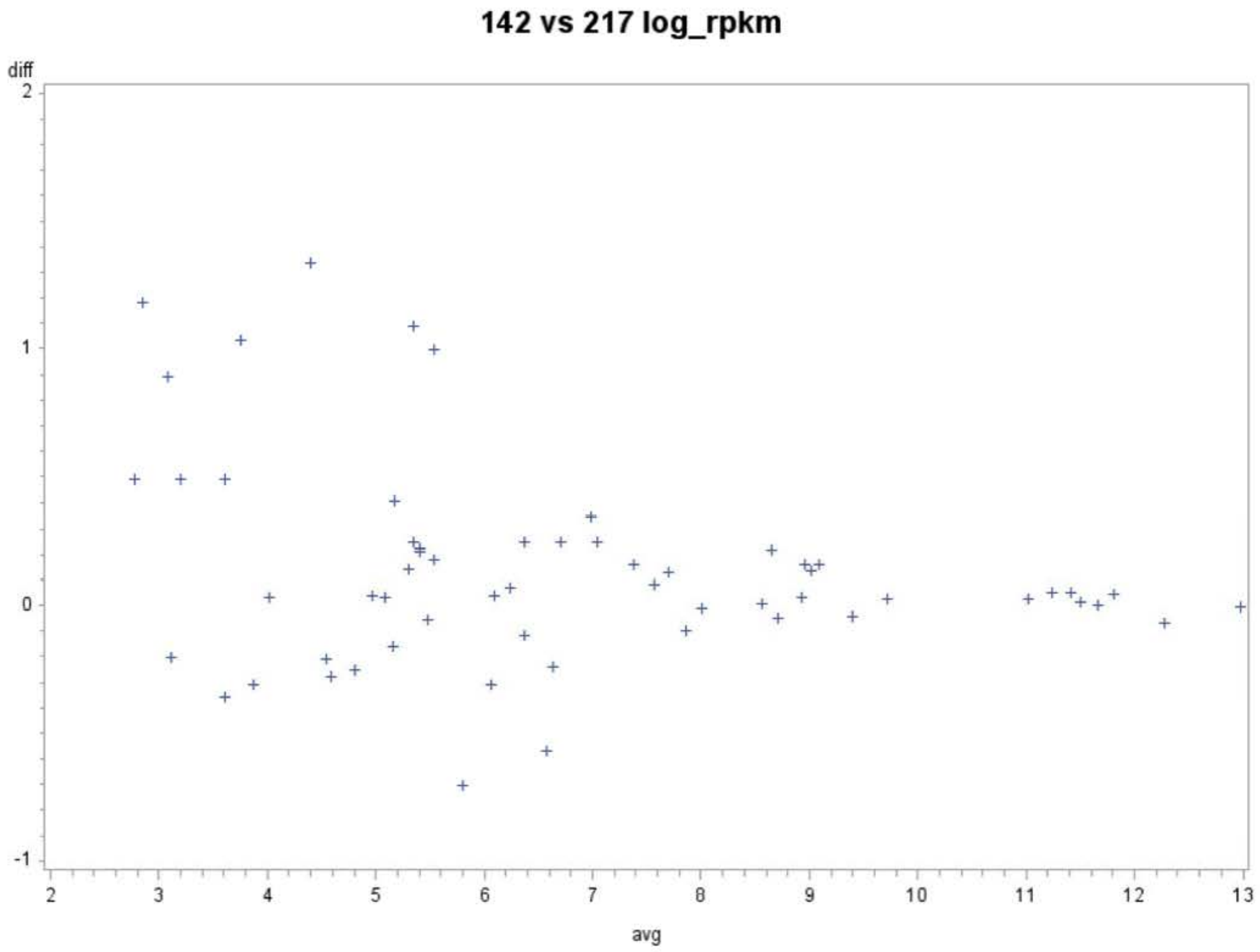
142 vs 217 log_rpkm
diff
2
1
0
-1
2
3
4
5
6
7
8
9
10
11
12
13
avg

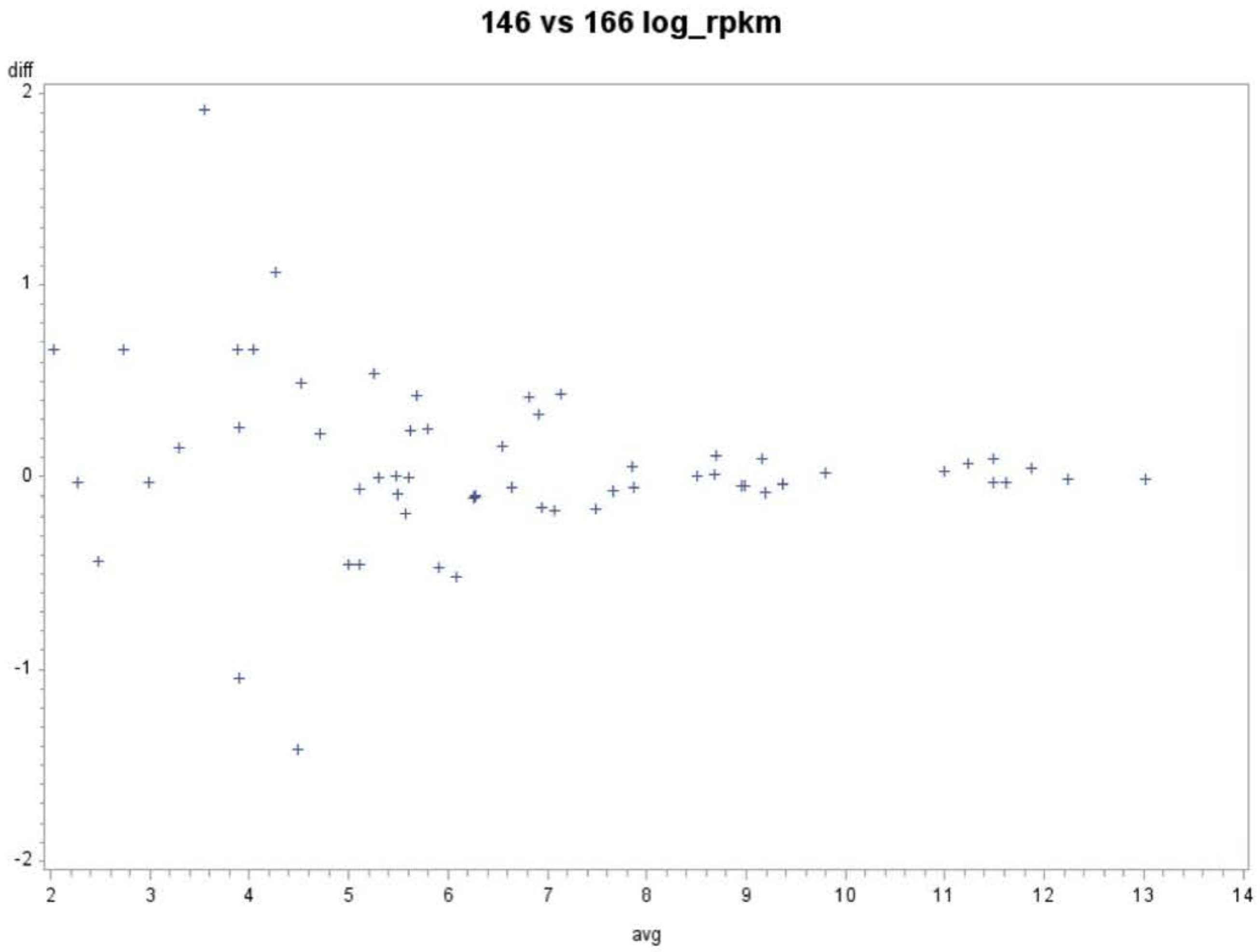

146 vs 166 log_rpkm
diff
2
1
0
-1
-2
2
3
4
5
6
7
8
9
10
11
12
13
14
avg

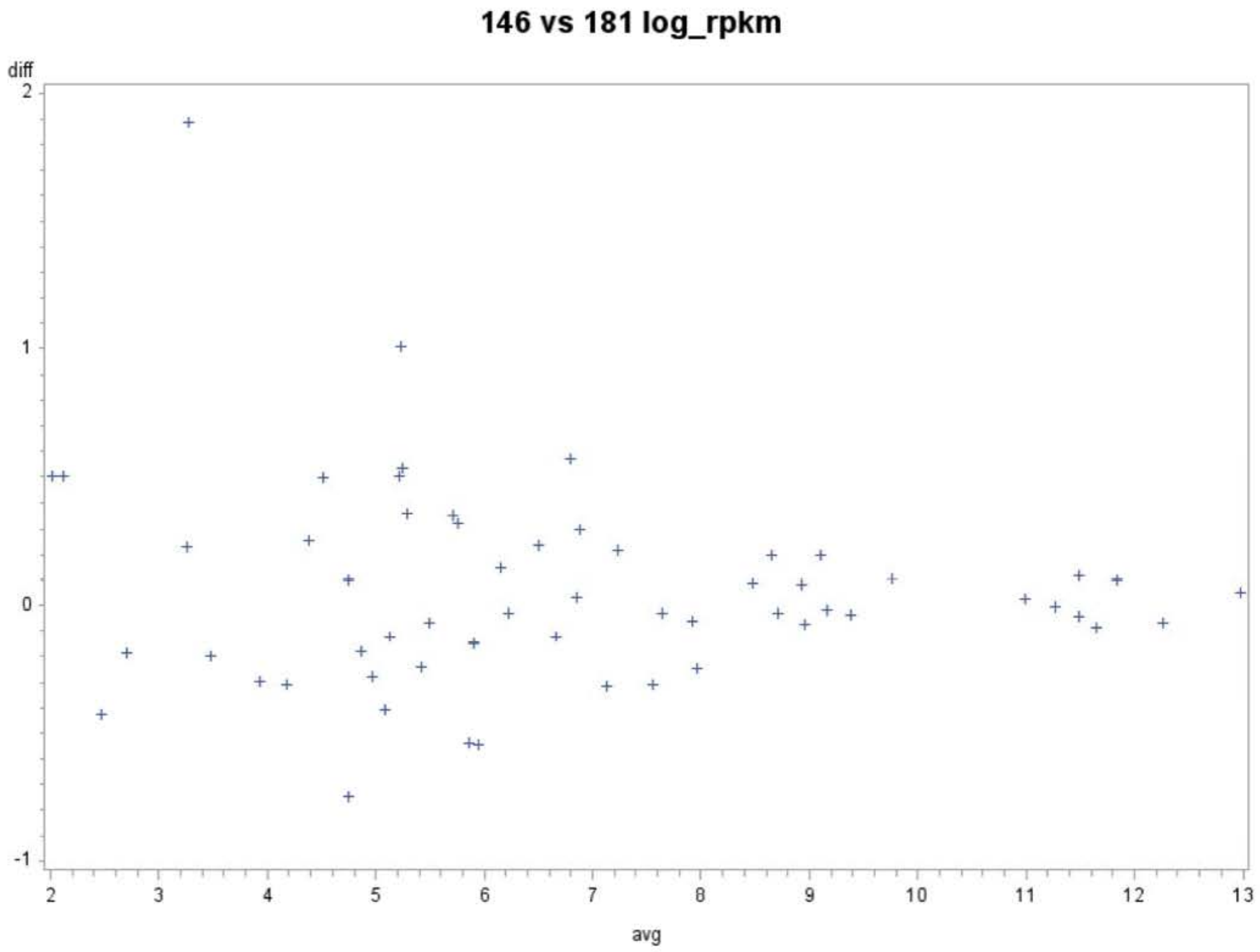
146 vs 181 log_rpkm
diff
2
1
0
-1
2
3
4
5
6
7
8
9
10
11
12
13
avg

146 vs 217 log_rpkm

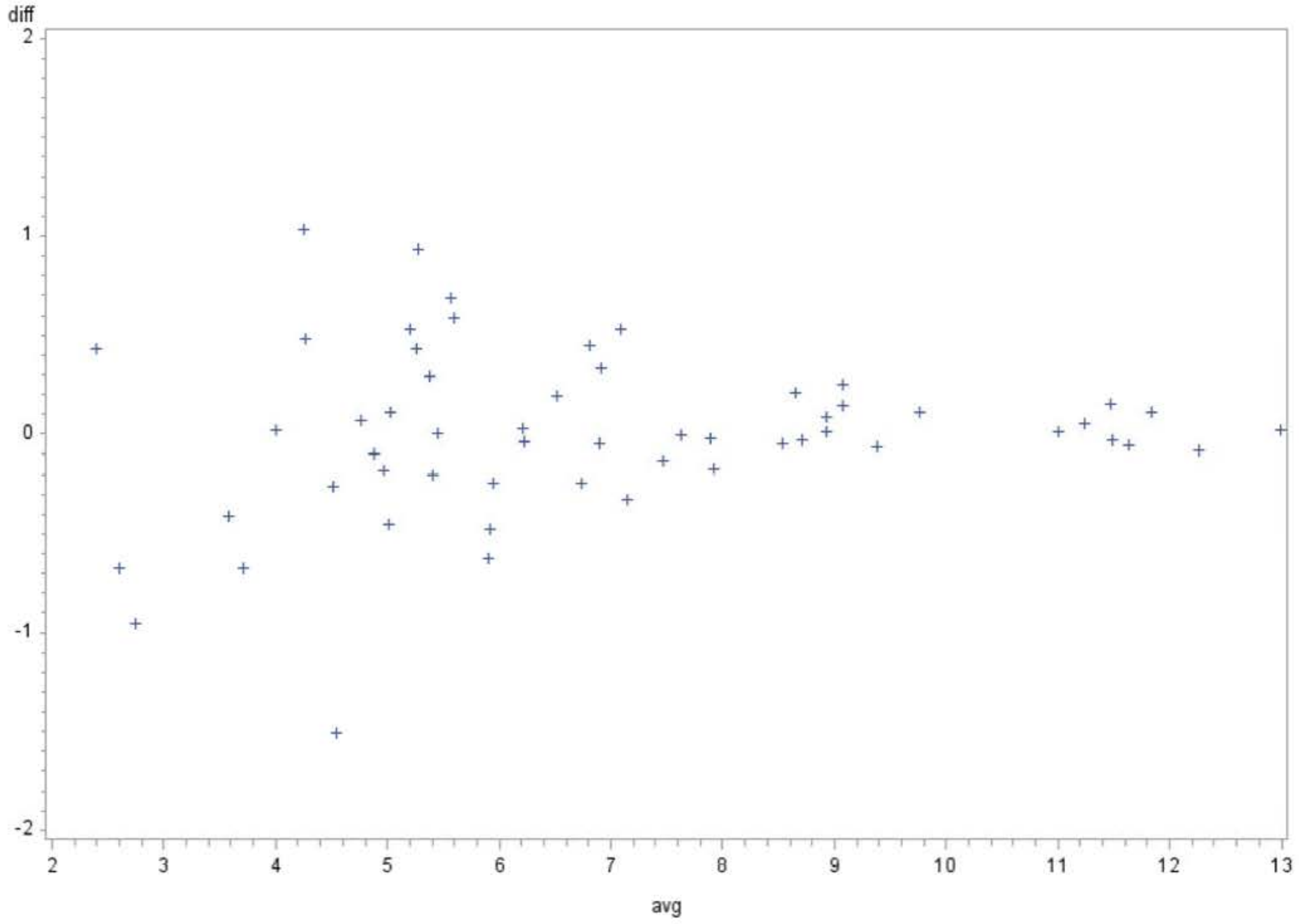

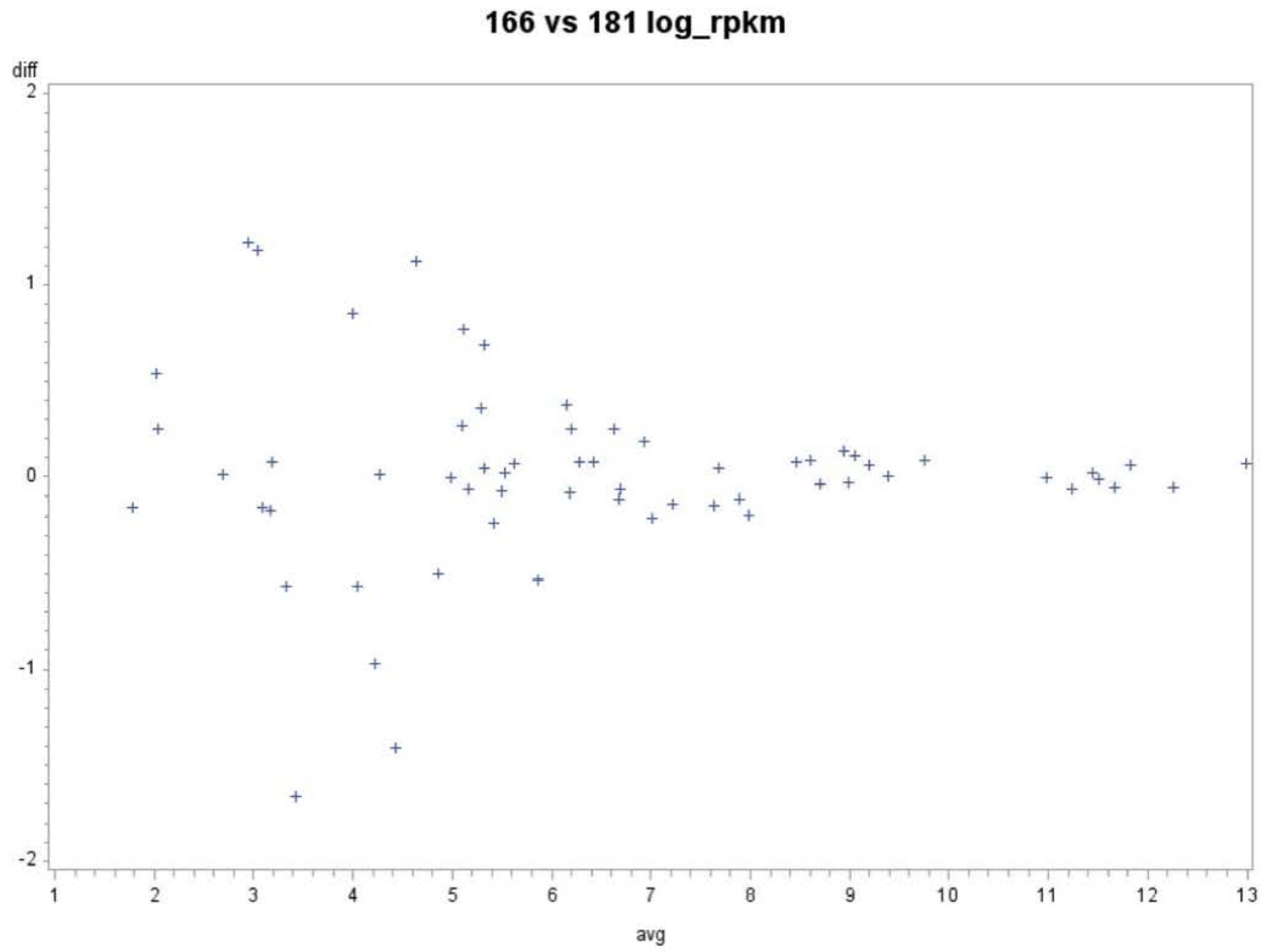

166 vs 181 log_rpkm
diff
2
1
0
-1
-2
1
2
3
4
5
6
7
8
9
10
11
12
13
avg

166 vs 217 log_rpkm

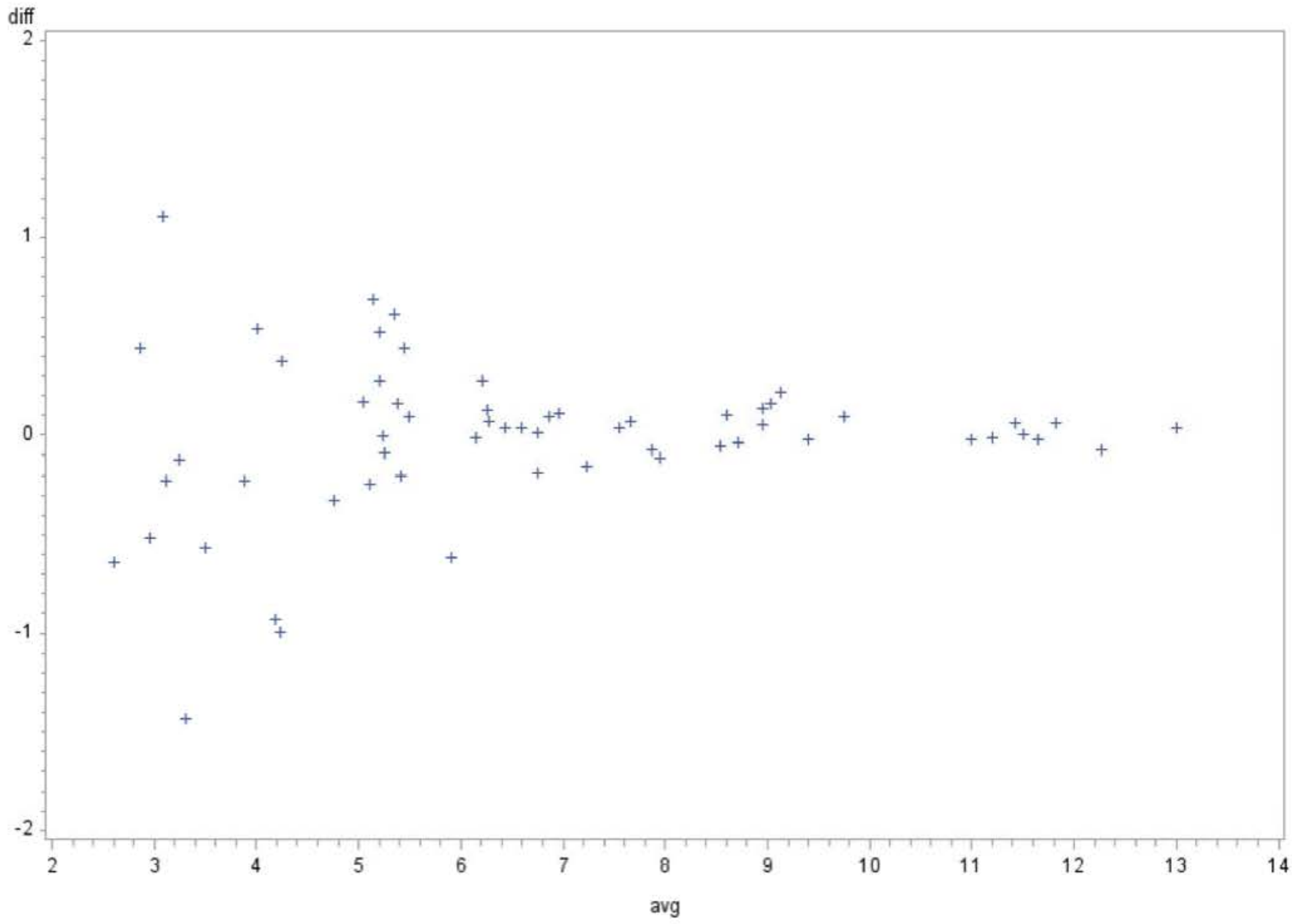

181 vs 217 log_rpkm

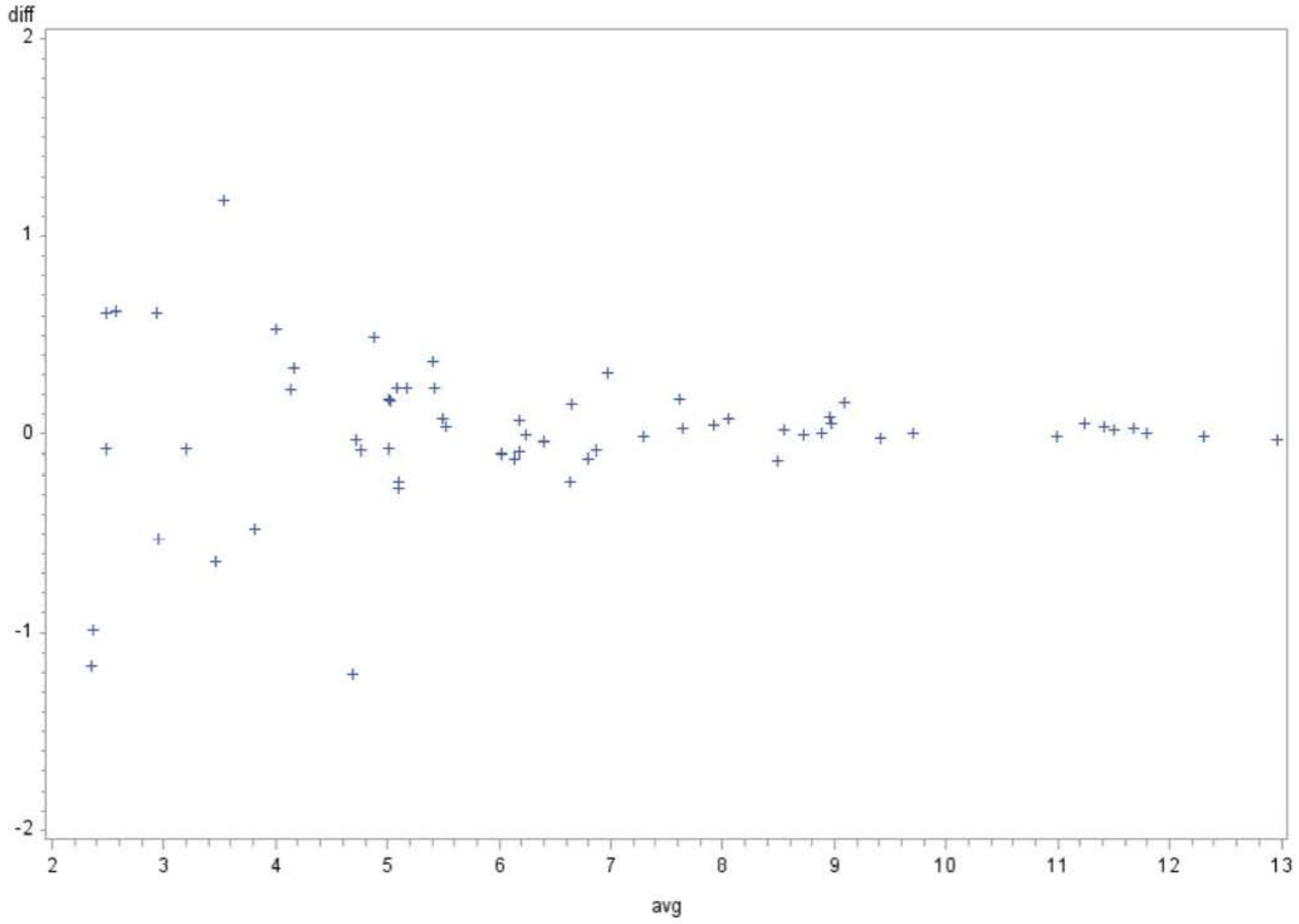

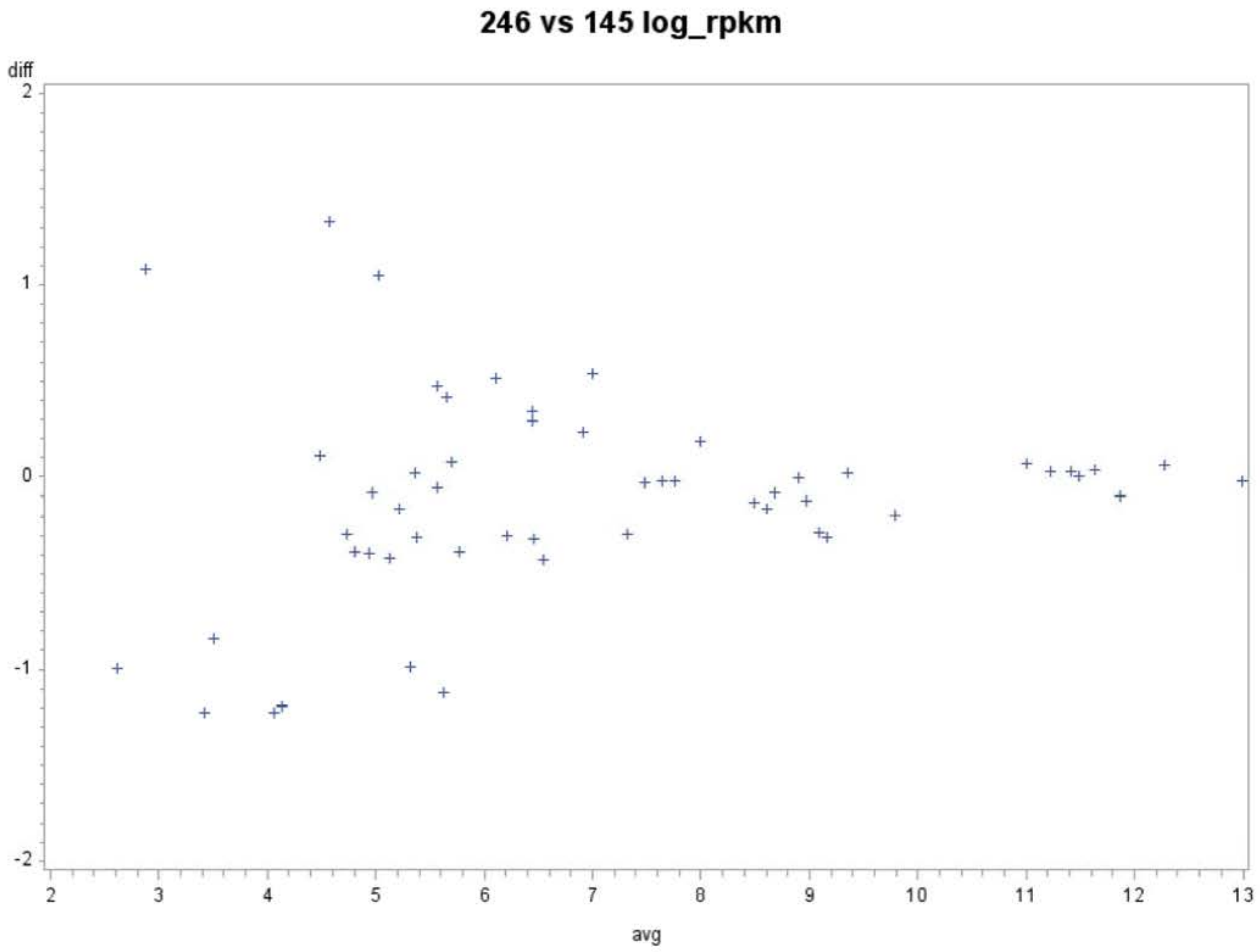
246 vs 145 log_rpkm
diff
2
1
0
-1
-2
2
3
4
5
6
7
8
9
10
11
12
13
avg

246 vs 170 log_rpkm

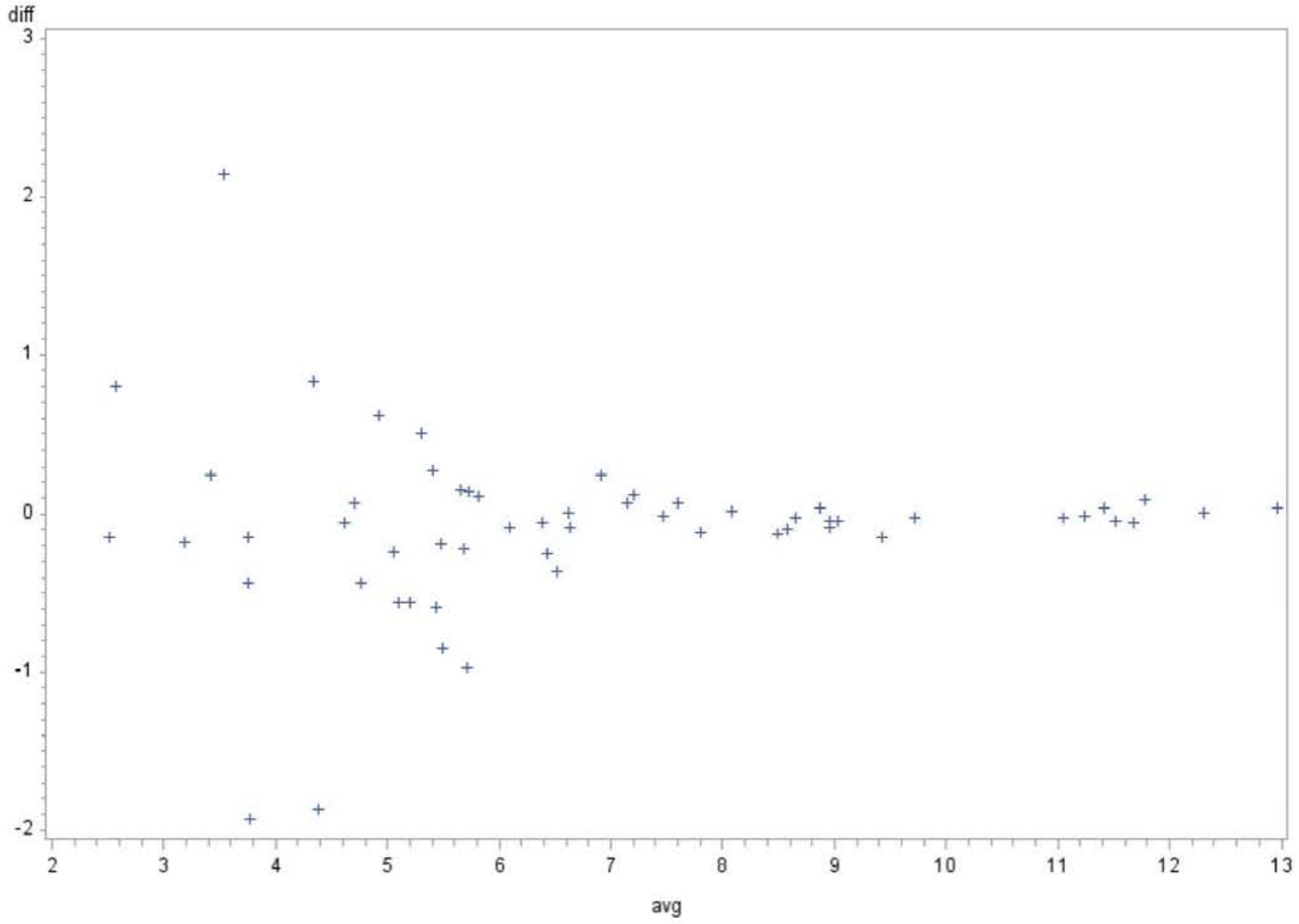

246 vs 188 log_rpkm

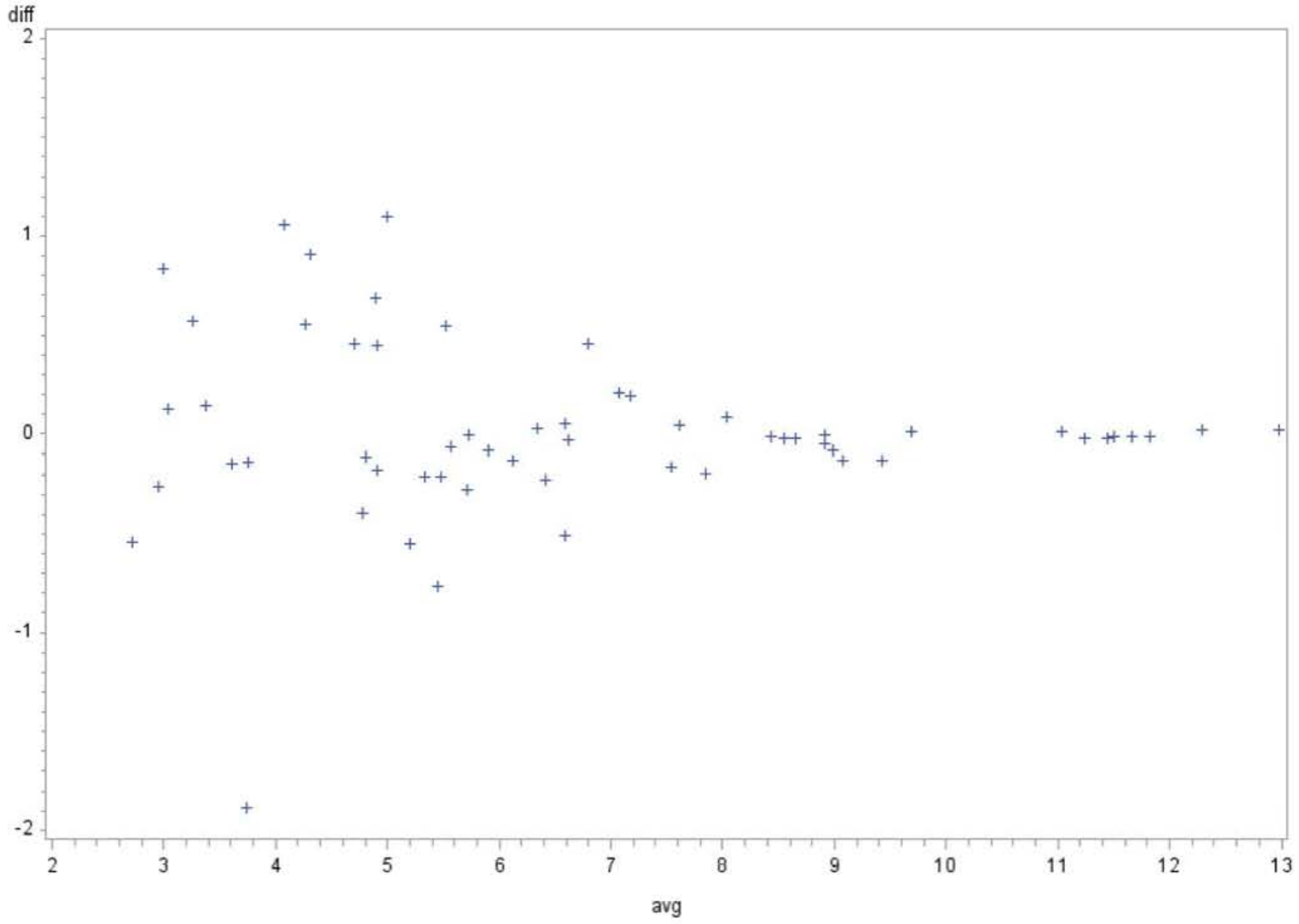

246 vs 204 log_rpkm

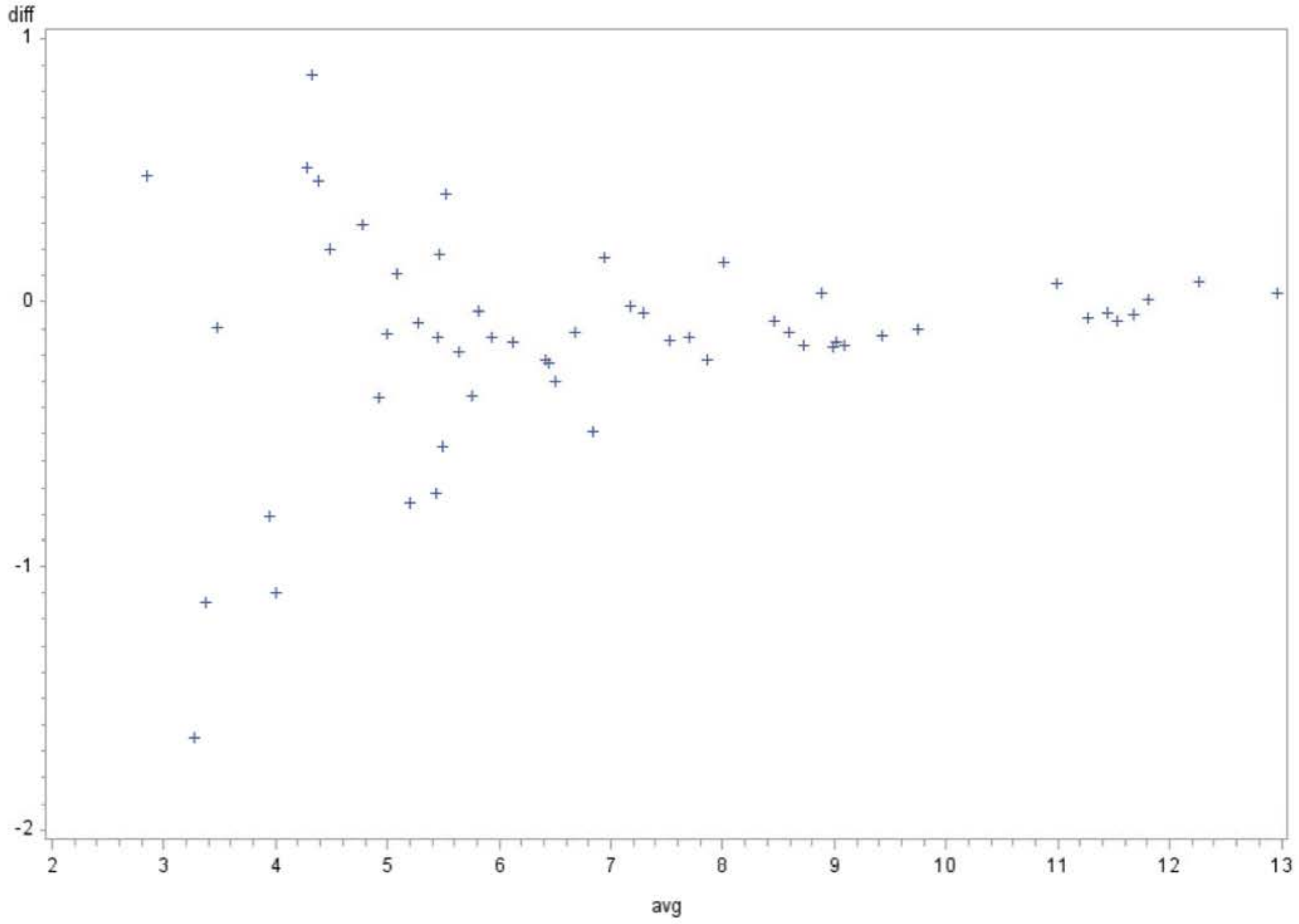

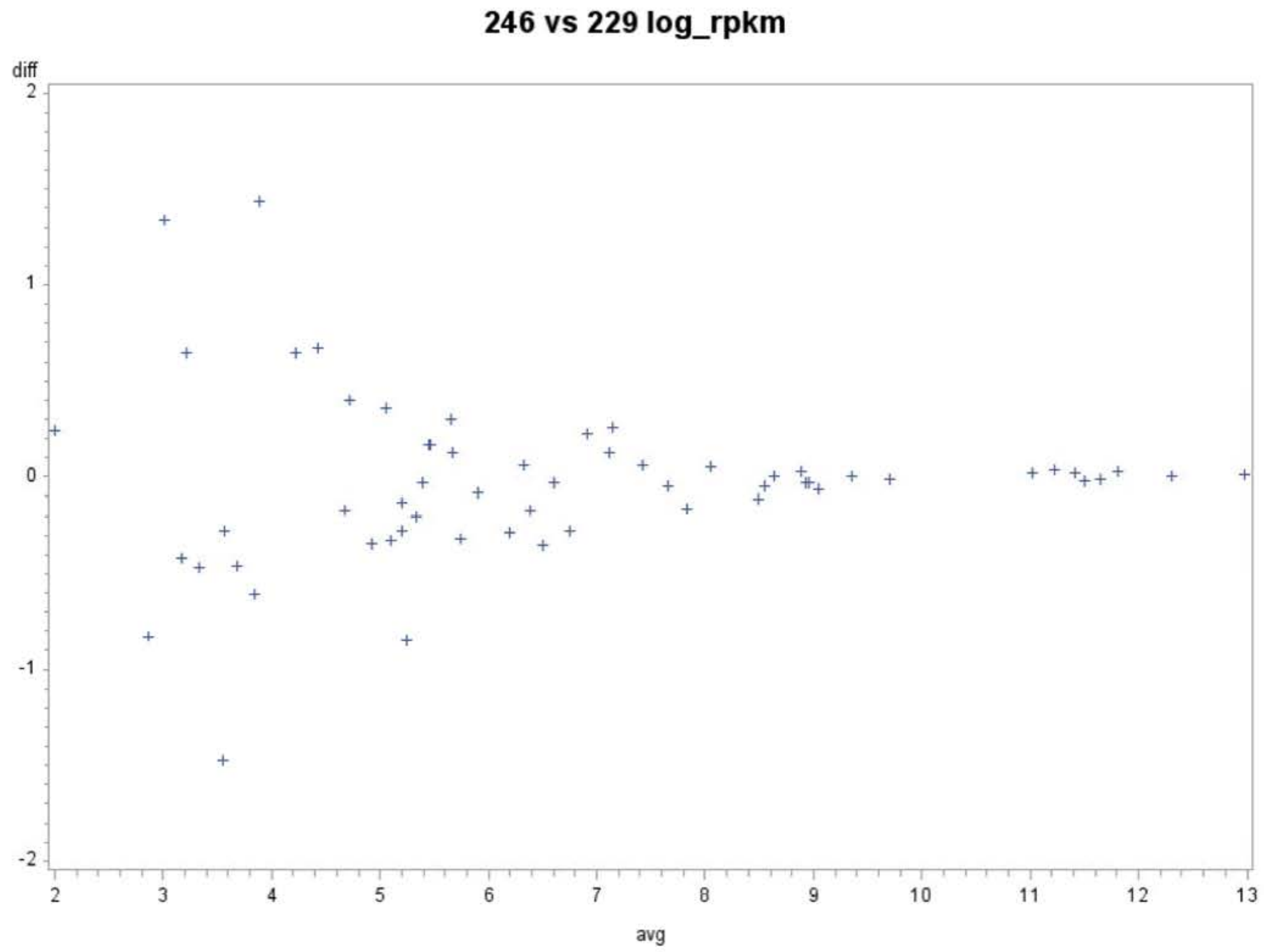
246 vs 229 log_rpkm
diff
2
1
0
-1
-2
2
3
4
5
6
7
8
9
10
11
12
13
avg

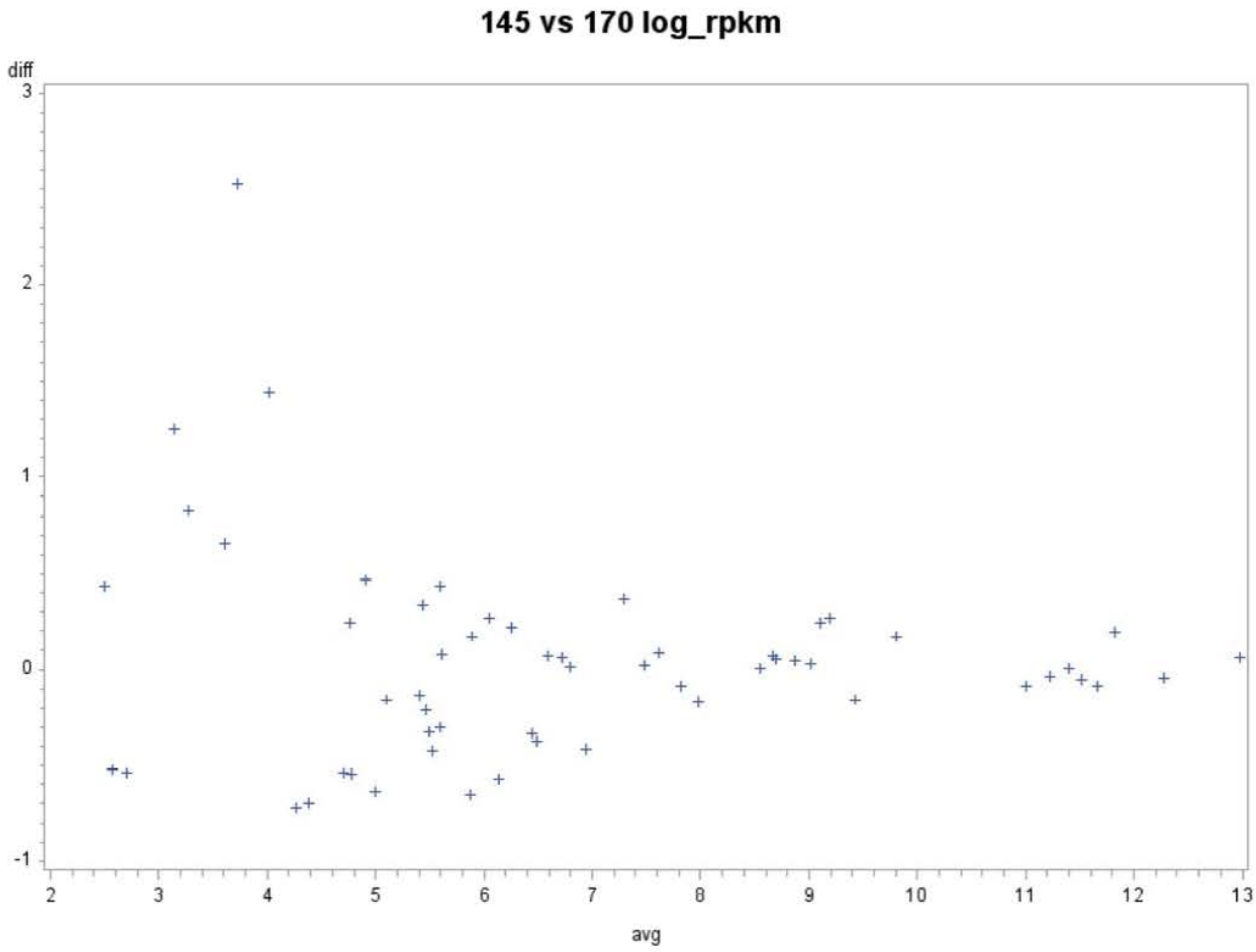

145 vs 170 log_rpkm
diff
3
2
1
0
-1
2
3
4
5
6
7
8
9
10
11
12
13
avg

145 vs 188 log_rpkm

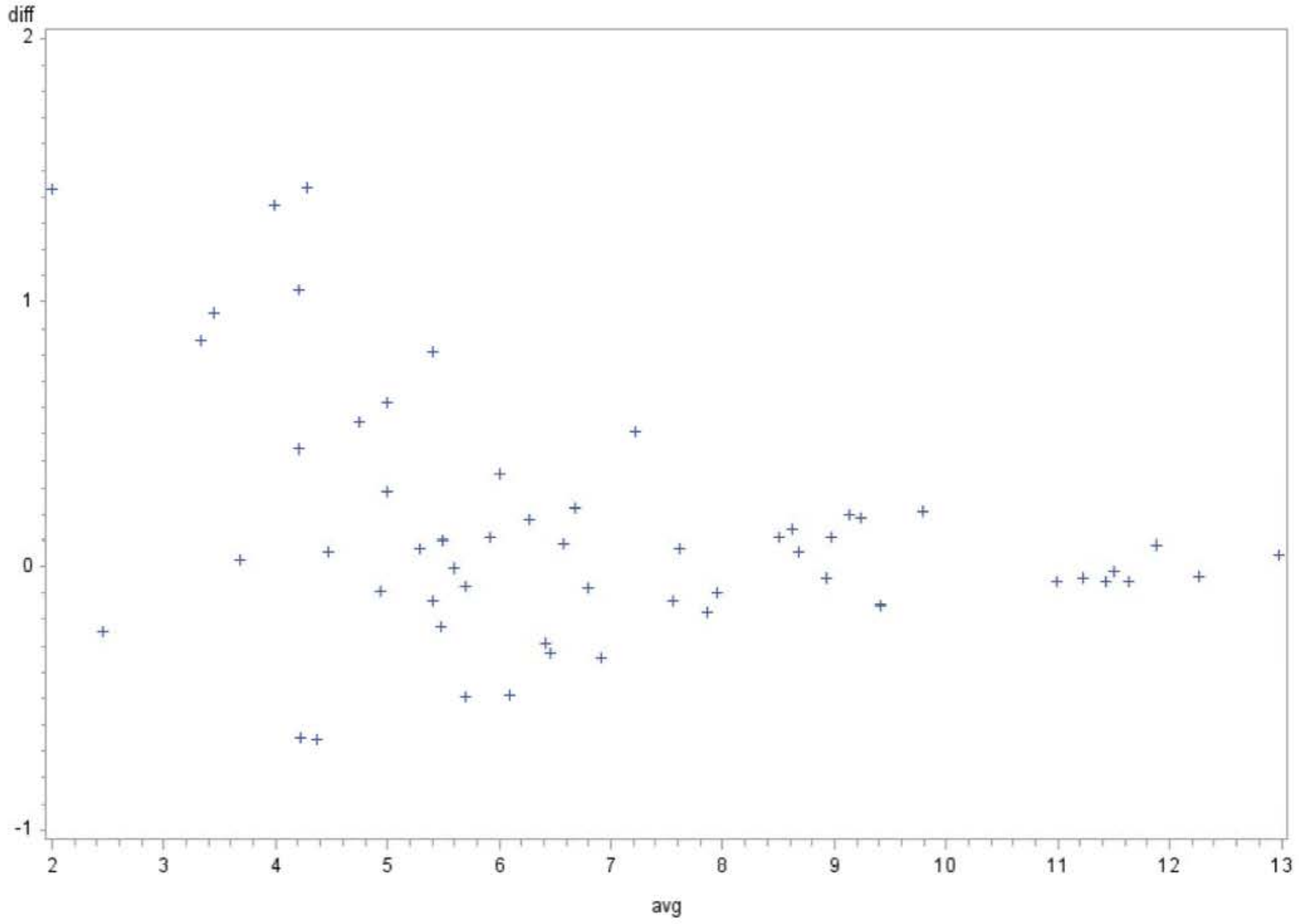

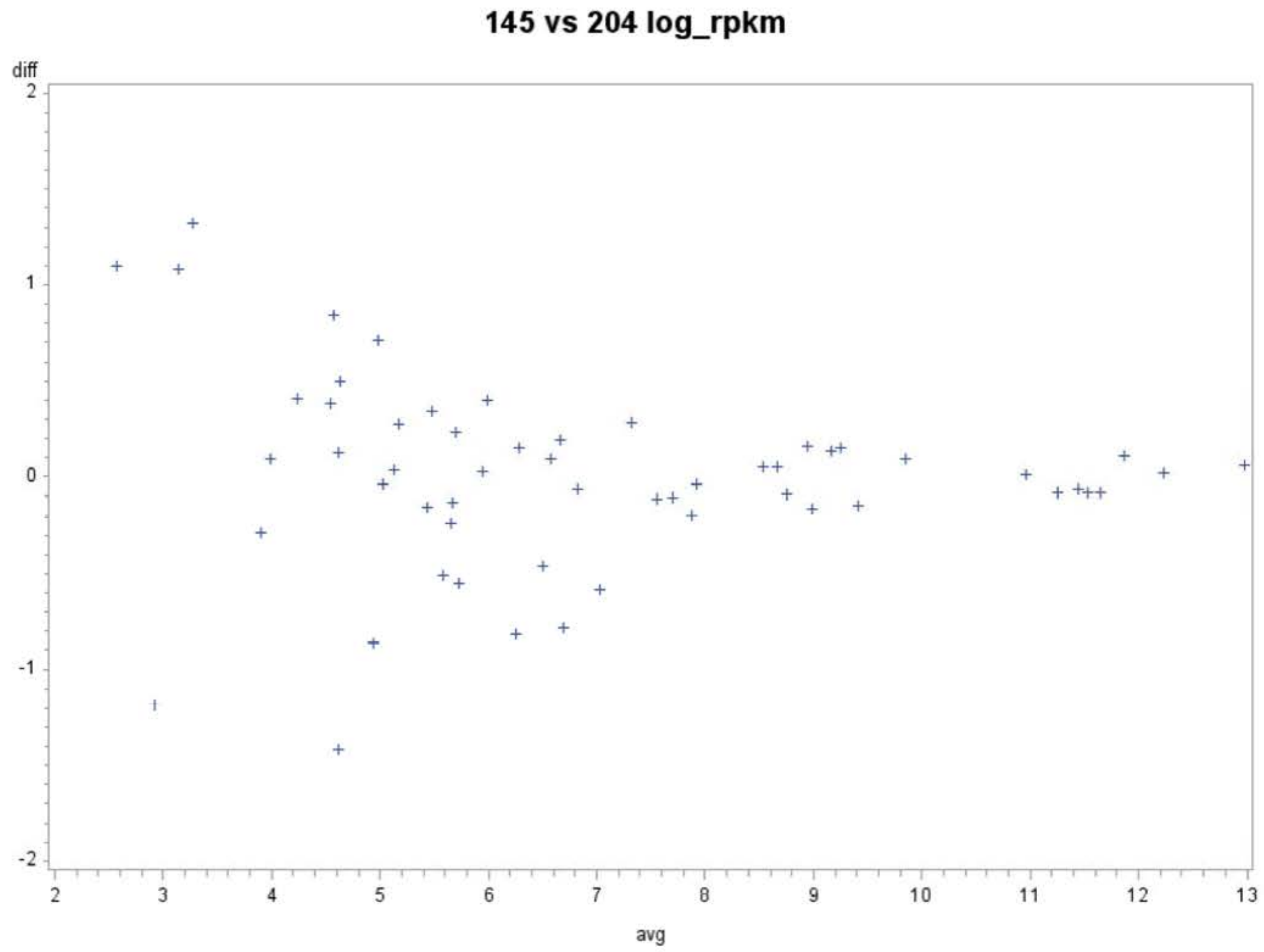
145 vs 204 log_rpkm
diff
2
1
0
-1
-2
2
3
4
5
6
7
8
9
10
11
12
13
avg

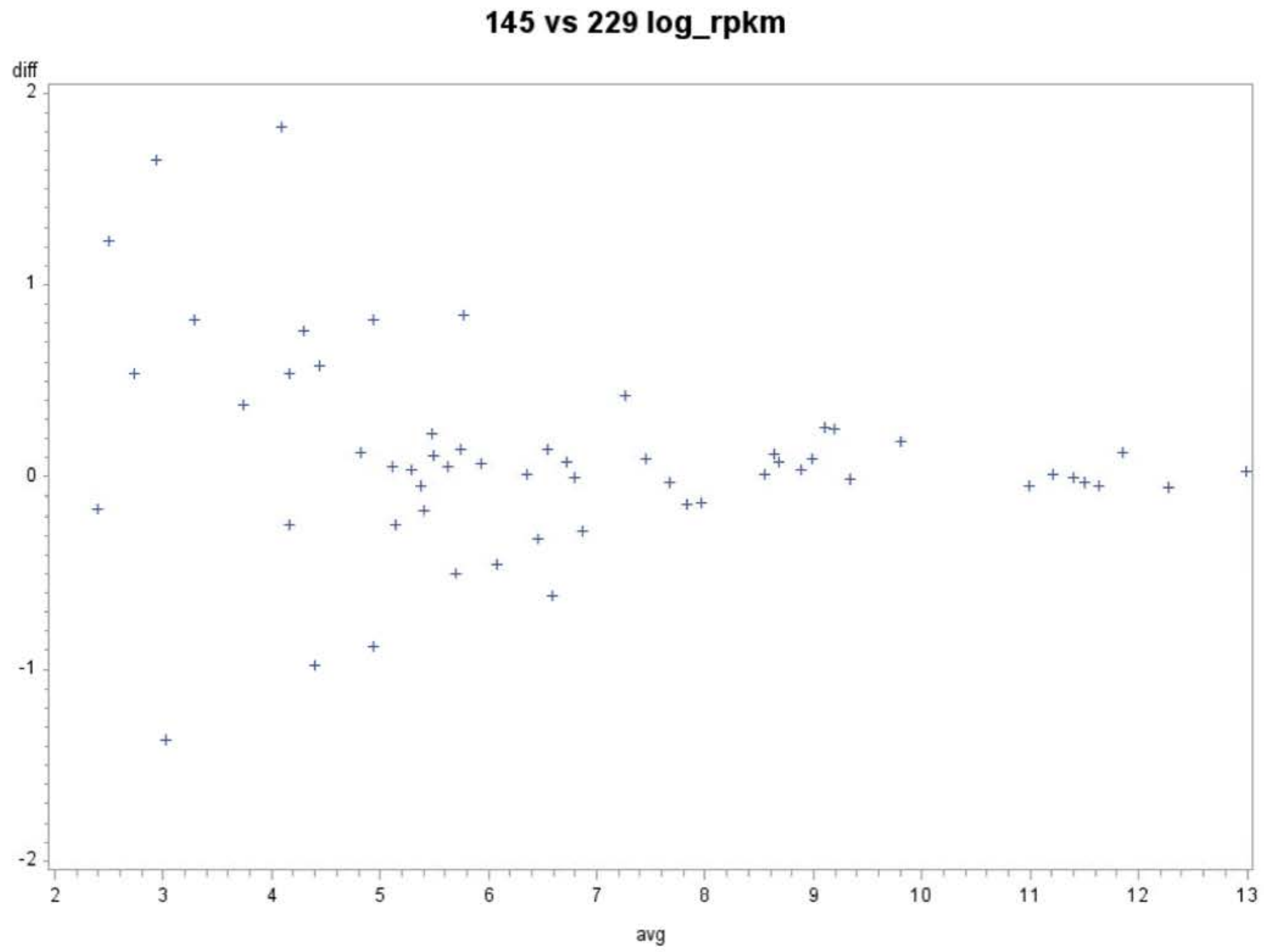
145 vs 229 log_rpkm
diff
2
1
0
-1
-2
2
3
4
5
6
7
8
9
10
11
12
13
avg

170 vs 188 log_rpkm

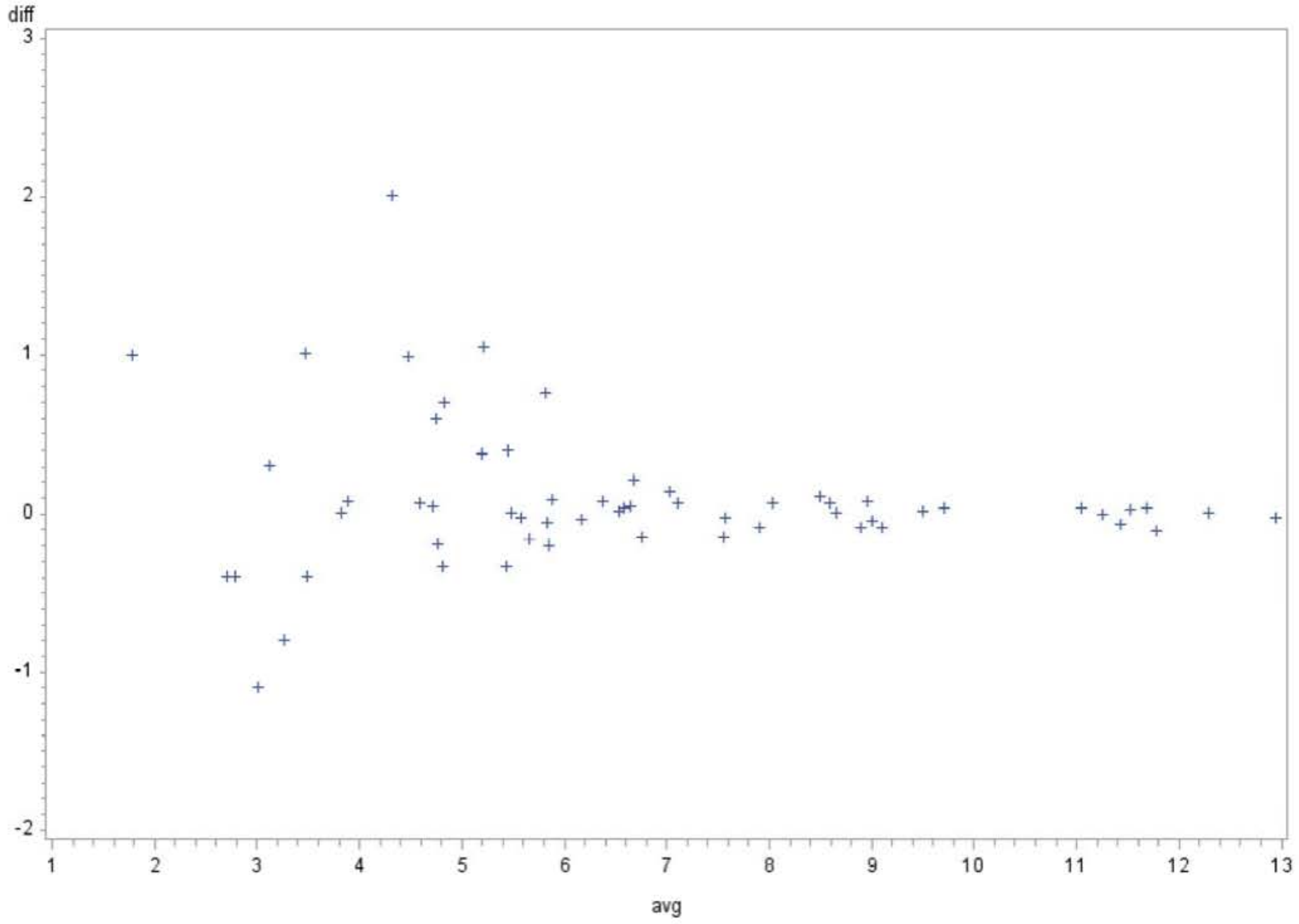

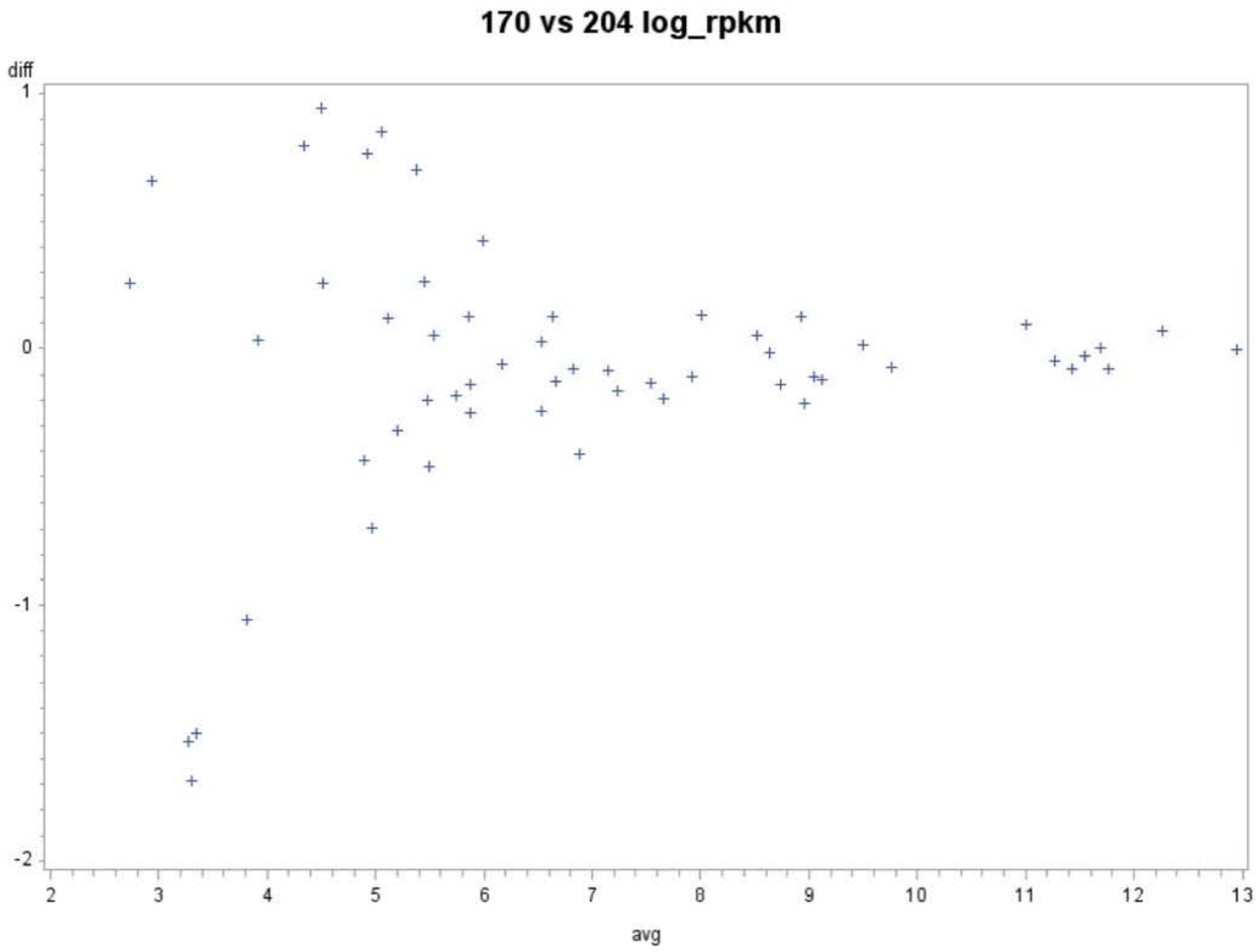
170 vs 204 log_rpkm
diff
1
0
-1
-2
2
3
4
5
6
7
8
9
10
11
12
13
avg

170 vs 229 log_rpkm

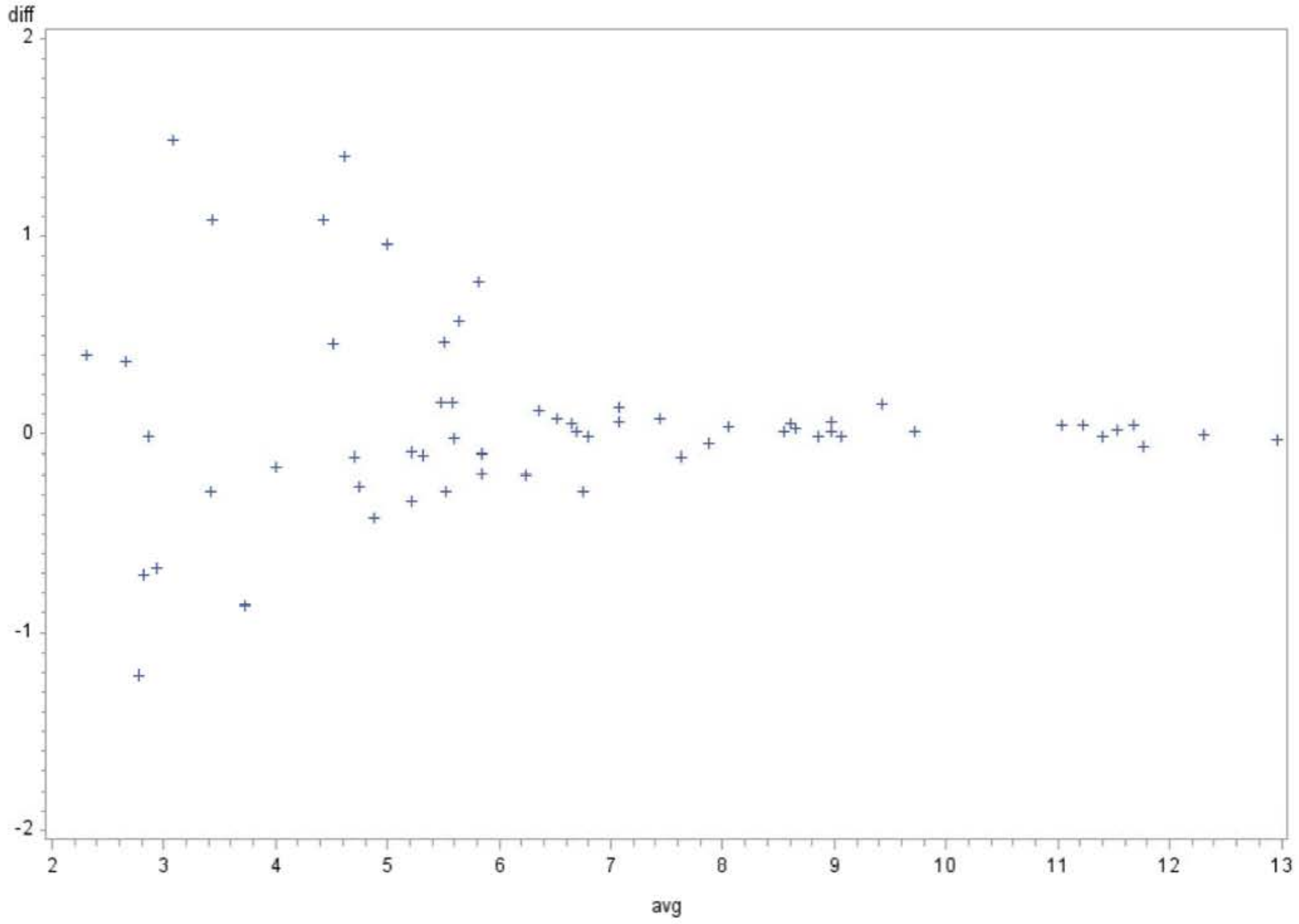

188 vs 204 log_rpkm

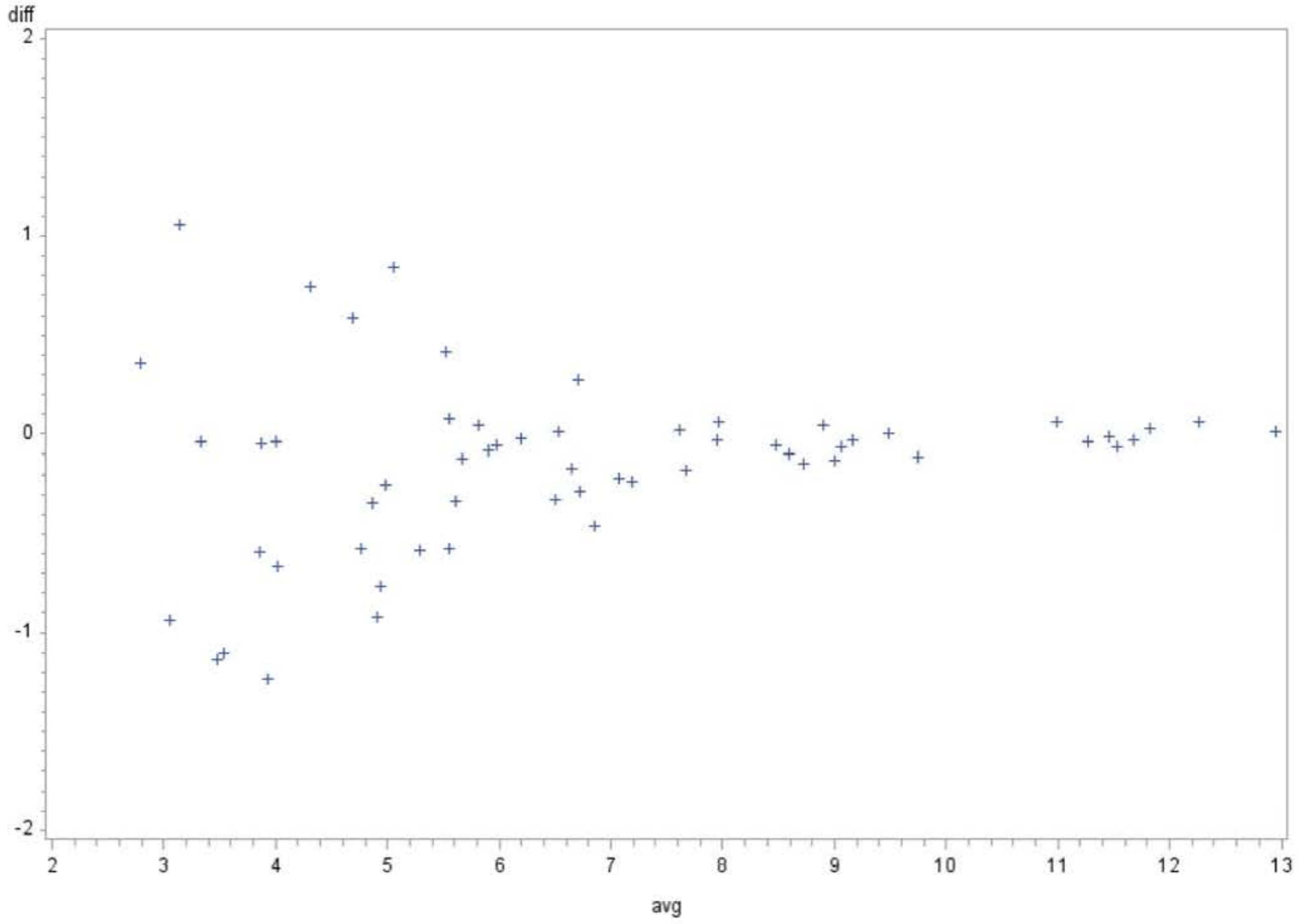

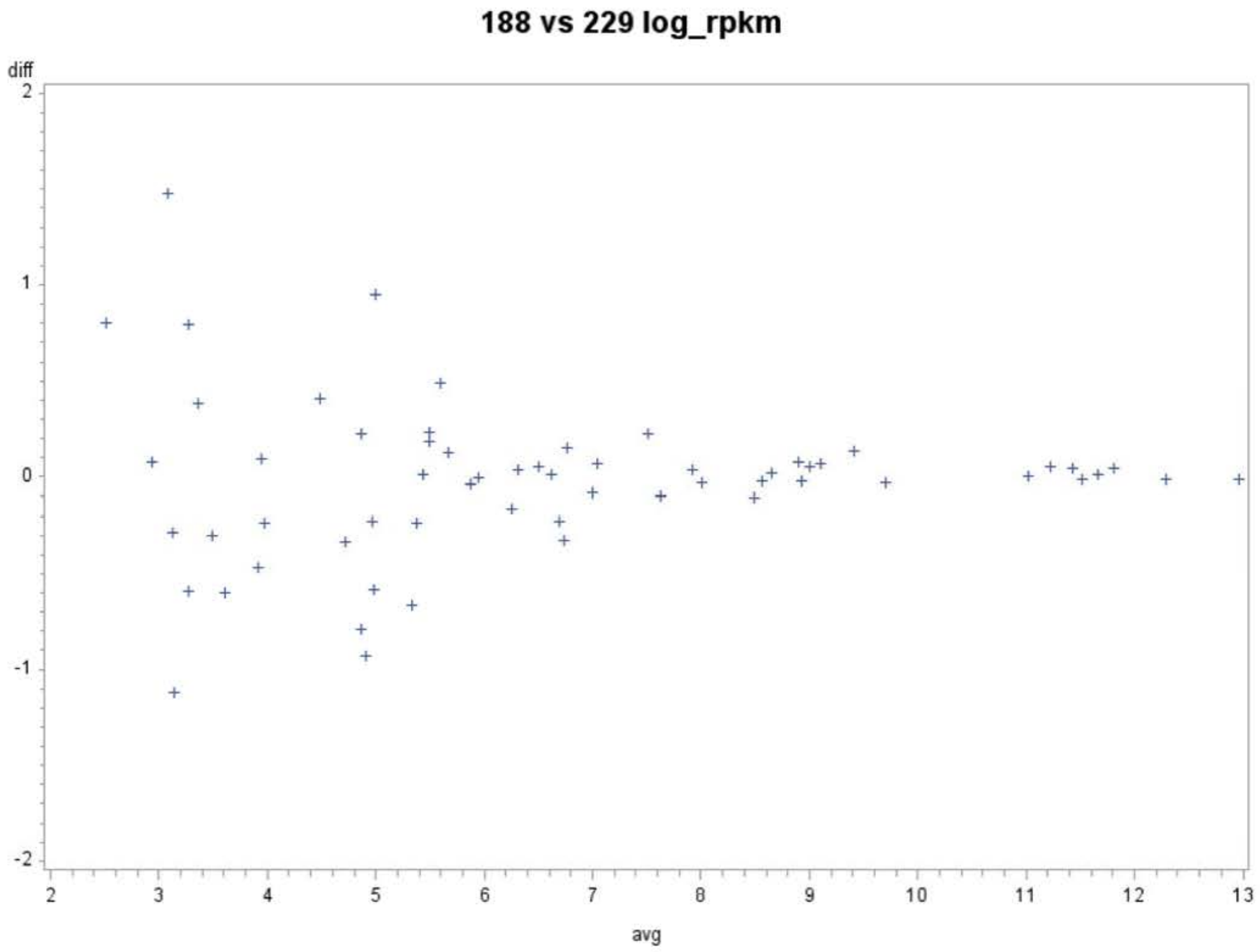
188 vs 229 log_rpkm
diff
2
1
0
-1
-2
2
3
4
5
6
7
8
9
10
11
12
13
avg

204 vs 229 log_rpkm

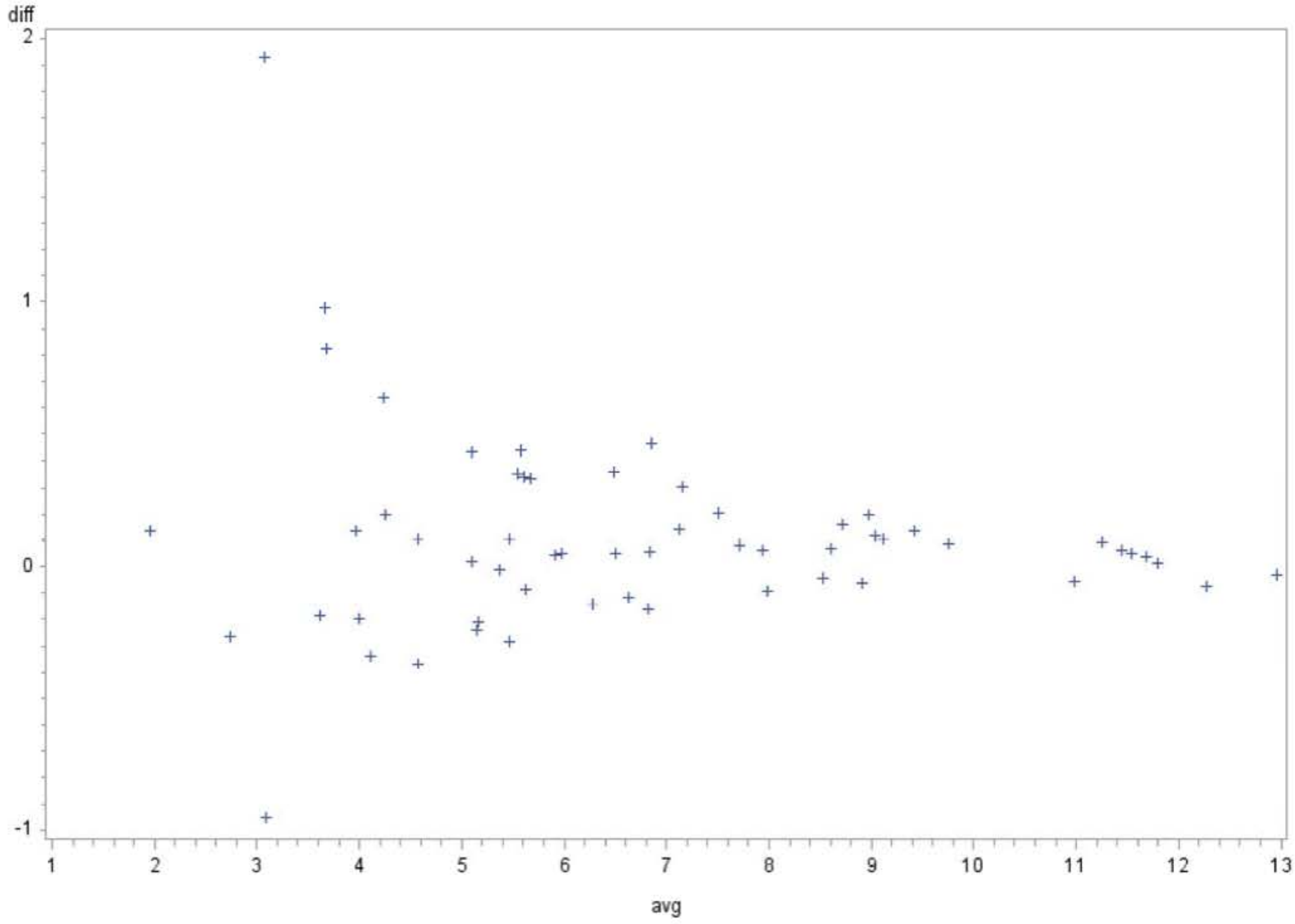

Supplement: Supplementary file 2 — Fig. S2 Bland–Altman plots for within‐group pairwise comparisons based on ERCC controls. [file NPH-217-871-s002.pdf]
